# Changes in Cerebral Hemodynamics during Complex Motor Learning by Character Entry into Touch-Screen Terminals

**DOI:** 10.1371/journal.pone.0140552

**Published:** 2015-10-20

**Authors:** Akira Sagari, Naoki Iso, Takefumi Moriuchi, Kakuya Ogahara, Eiji Kitajima, Koji Tanaka, Takayuki Tabira, Toshio Higashi

**Affiliations:** 1 Unit of Rehabilitation Sciences, Nagasaki University Graduate School of Biomedical Sciences, Nagasaki, Japan; 2 Japanese Red Cross Society Nagasaki Genbaku Hospital, Nagasaki, Japan; 3 Medical Corporation Tojinkai Miharadai Hospital, Nagasaki, Japan; 4 Faculty of Health and Social Work, School of Rehabilitation, Kanagawa University of Human Services, Kanagawa, Japan; 5 Center for Industry, University and Government Cooperation, Nagasaki University, Nagasaki, Japan; 6 Unit of Physical and Occupational Therapy, Nagasaki University Graduate School of Biomedical Sciences, Nagasaki, Japan; 7 Faculty of Rehabilitation Sciences, Nishikyushu University, Saga, Japan; University of Electronic Science and Technology of China, CHINA

## Abstract

**Introduction:**

Studies of cerebral hemodynamics during motor learning have mostly focused on neurorehabilitation interventions and their effectiveness. However, only a few imaging studies of motor learning and the underlying complex cognitive processes have been performed.

**Methods:**

We measured cerebral hemodynamics using near-infrared spectroscopy (NIRS) in relation to acquisition patterns of motor skills in healthy subjects using character entry into a touch-screen terminal. Twenty healthy, right-handed subjects who had no previous experience with character entry using a touch-screen terminal participated in this study. They were asked to enter the characters of a randomly formed Japanese syllabary into the touch-screen terminal. All subjects performed the task with their right thumb for 15 s alternating with 25 s of rest for 30 repetitions. Performance was calculated by subtracting the number of incorrect answers from the number of correct answers, and gains in motor skills were evaluated according to the changes in performance across cycles. Behavioral and oxygenated hemoglobin concentration changes across task cycles were analyzed using Spearman’s rank correlations.

**Results:**

Performance correlated positively with task cycle, thus confirming motor learning. Hemodynamic activation over the left sensorimotor cortex (SMC) showed a positive correlation with task cycle, whereas activations over the right prefrontal cortex (PFC) and supplementary motor area (SMA) showed negative correlations.

**Conclusions:**

We suggest that increases in finger momentum with motor learning are reflected in the activity of the left SMC. We further speculate that the right PFC and SMA were activated during the early phases of motor learning, and that this activity was attenuated with learning progress.

## Introduction

Humans accomplish a wide variety of motor and cognitive tasks in everyday life. Furthermore, we continue acquiring new complex motor skills to overcome various challenges. Therefore, it is important to study the underlying changes in cerebral hemodynamics during motor-cognitive adaptation. In recent years, various functional brain imaging techniques that enable noninvasive visualization of brain activity have advanced greatly [1–8]. Cerebral dynamics during motor-cognitive task adaptations have been investigated by employing various neuroimaging techniques, such as positron emission tomography (PET) or functional magnetic resonance imaging (fMRI), while the subject is in a supine position [9–14]. However, in daily life, motor-cognitive adaptation tasks are usually performed in upright positions such as sitting or standing. To address the issue of limited ecological validity and enable task performance in a more natural setting, we used near-infrared spectroscopy (NIRS) as a less-limiting imaging method that enables measurement of cortical activation during activities of daily life.

Although NIRS is characterized by marked limitations in temporal and spatial resolution, its safety, non-restrictiveness, and portability enable broader and more flexible use than other brain imaging methods [15]. A number of studies have examined cerebral hemodynamics using NIRS during a wide variety of motor activities such as a walking or running [16–20], cycling [21–23], apple peeling [24], and finger tapping [25–28]. Additionally, researchers have examined cerebral hemodynamics during cognitive tasks such as trail making [29–32], the rock-paper-scissors game [33,34], maze navigation [35], and sequential finger touching [36,37].

To our knowledge, NIRS studies that examine motor skill learning with ongoing adaptation processes are rare. Two NIRS studies have reported changes of hemodynamics over time related to motor skill learning using eye-hand coordination in pursuit rotor [38] or target reaching tasks [39]. These NIRS studies evaluated specific sensorimotor tasks. However, these studies did not address changes in hemodynamic activity during motor learning of complex skills with concurrent cognitive processing such as working memory and executive function. Gentili et al. reported changes in cerebral hemodynamics as measured by NIRS during performance of a motor-cognitive adaptation task and demonstrated activation only in the prefrontal cortex (PFC) [40]. However, the NIRS probe in their study only covered the forehead. Therefore, no studies are available that examine the hemodynamic responses of other cortical regions during performance of motor-cognitive adaptation tasks using a NIRS system that also includes the posterior half-head.

Information technology has substantially affected modern society, and researchers concerned with neurorehabilitation should examine interventions relevant to the lifestyle of modern people. In recent years, the number of touch-screen terminal users has increased remarkably and smartphones have spread all over the world. eMarketer, a U.S. research company, speculated that the number of smartphone users will total 1.75 billion in 2014 and further increase to 2.5 billion in 2017 [41]. The operation of a smartphone requires flick inputs to create an email sentence in Japanese, and the entry of the required characters is difficult and will need to be considered in the field of neurorehabilitation in the future. However, there are no studies measuring cerebral hemodynamics related to motor skill learning that address the underlying complex cognitive processing in tasks such as character entry into a touch-screen terminal. Therefore, in this study, we examined cerebral hemodynamics using NIRS associated with acquisition patterns of motor-cognitive skills in healthy subjects using character entry into a touch-screen terminal. Based on previous studies, we hypothesized that the behavioral improvements resulting from adaptation would be accompanied by distinct patterns of activation over the various cortical regions.

## Methods

### Subjects

Twenty healthy subjects (9 men and 11 women; mean age, 27.5±5.5 years) participated in this study. All subjects were self-reported as right-handed (S1 Dataset). Exclusionary criteria included any medical illness affecting central nervous system function, psychiatric or neurological disorders, history of head trauma, or current substance abuse. None of the subjects had previous experience with character entry using a touch-screen terminal. Written informed consent was obtained from each subject. The study was approved by the local ethics committee of Nagasaki University Graduate School of Biomedical and Health Sciences. All of the experimental procedures were conducted in accordance with the Declaration of Helsinki.

### Tasks and procedures

**Fig 1:**
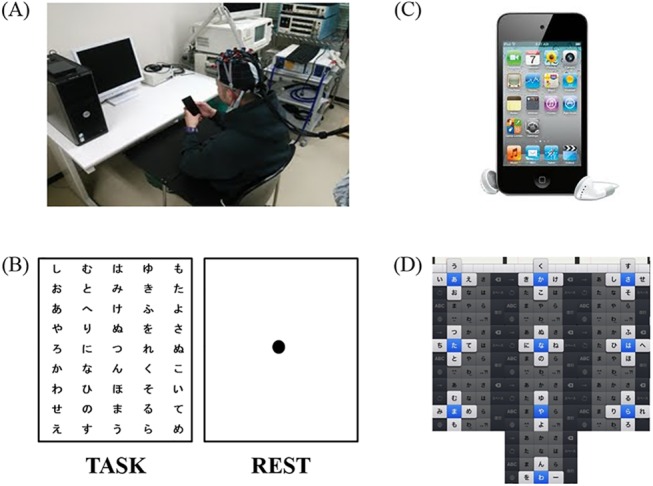
Experimental setup of touch-screen terminal task. (A) Experimental setup of character entry into touch-screen terminal showing a subject with the custom-made cap with the NIRS device. (B) Display of task and rest conditions. The display showing the task condition changed with each cycle. (C) The iPod Touch 4. The character entry into screen mode was used to measure the number of character entries. All subjects performed the task with their right thumb for 15 s interleaved with rest periods of 25 s for 30 repetitions. (D) Methods of character entry into touch-screen terminal. All subjects were asked to enter the characters of a randomly formed Japanese syllabary into the touch-screen terminal.

The subjects sat on a chair 80 cm away from a PC monitor (Epson LD1957S, Japan, 19-inch, resolution: 1024 × 768 pixels) (Fig 1A). All subjects performed the task with their right thumb for 15 s alternating with 25 s of rest for 30 repetitions (cycles 1 to 30) (Fig 1B). Gains in motor skills were evaluated according to the number of characters entered into the touch-screen terminal (Apple iPod Touch 4, Japan, 3.5-inch, resolution: 960×640 pixels) (Fig 1C). All subjects were asked to enter the characters of a randomly formed Japanese syllabary presented on a PC monitor, starting with the character from the upper left, into the touch-screen terminal as quickly as possible (Fig 1D). The character string in the monitor was constructed in five lines and nine rows, resulting in 45 characters. The characters of the randomly formed Japanese syllabary were changed every cycle (S1 Supporting Information). All subjects were asked to fixate on a single point at the center of the screen during rest and to stay relaxed. In English, the letter combinations “P Q R S” and “W X Y Z” involve a three-way operation in the upper, right, and left direction, whereas the input otherwise involves operation in two ways. In Japanese, the combinations “ya yu yo” and “wa wo n” are two-way operations in the right and left directions, whereas the input otherwise operates in four ways in the upper, lower, right, and left directions. Therefore, operation of these devices using Japanese input is more complicated than that using English input. The task of the present study was not simply a matter of entering as many characters as possible; the only characters that were meant to be entered were those displayed. There was thus a trade-off between the number of characters and the number of incorrect entries. Thus, the task performance of each subject was calculated by subtracting the number of errors from the number of correct answers in each cycle.

**Fig 2:**
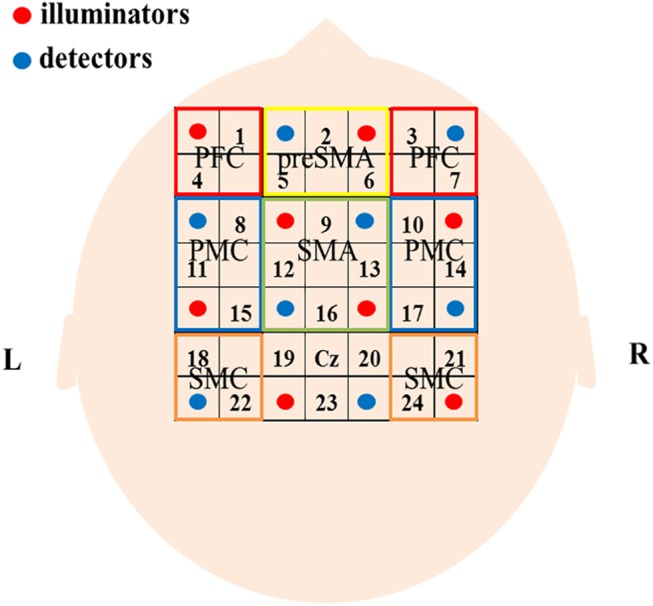
Location of the optodes and brain areas. Sixteen optodes, comprising 8 light source fibers (red) and 8 detectors (blue) that enabled 24-channel measurements, were arranged on the scalp. The channels covering SMC, SMA, preSMA, PMC, and PFC are shown. See text for details.

NIRS measurements were performed using a continuous wave system (ETG-4000, Hitachi Medical Corp., Tokyo, Japan) equipped with 4 × 4 optode probe sets (8 incident light and 8 detector fibers), resulting in a total of 24 channels with an inter-optode distance of 3.0 cm. The continuous-wave NIRS system utilizes two different wavelengths (~625 and 830 nm). Relative changes in the absorption of near-infrared light were sampled at 10 Hz, and these measures were converted into related concentration changes for oxygenated hemoglobin (oxy-Hb) and deoxygenated hemoglobin (deoxy-Hb), based on the modified Beer-Lambert approach [42]. The moving average method (window: 5 s) was used to exclude short-term motion artifacts in the analyzed data. The obtained data were analyzed in the integral mode, which calculates average waveform. Pre-task baseline was defined as the 5-s period immediately prior to task onset. In this study, we used changes in oxy-Hb concentration as an indicator of changes in regional cerebral blood volume, as an earlier NIRS signal study using a perfused rat brain model proposed that oxy-Hb, rather than deoxy-Hb, is the more sensitive parameter for the study of activation [43]. The NIRS channels were placed according to the international 10–20 system [44]. Regarding the positions of the optodes, we followed previous NIRS studies of motor-related areas [37,45]. The optodes were positioned using a custom-made cap that covered the right and left PFC, presupplementary motor area (preSMA), supplementary motor area (SMA), dorsal premotor cortex (PMC), and sensorimotor cortex (SMC). The areas and optodes were as follows: left SMC, channels 18 and 22; right SMC, channels 21 and 24; motor area, channels 19, 20, and 23; SMA, channels 9, 12, 13, and 16; preSMA, channels 2, 5, and 6; left PMC, channels 8, 11, and 15; right PMC, channels, 10, 14, and 17; left PFC, channels 1 and 4; and right PFC, channels 3 and 7. The Cz position in the international 10–20 system was used as a marker for ensuring replicable placement of the optodes (Fig 2).

## Data analysis

Changes in performance across task cycles were analyzed by calculating Spearman’s rank correlation coefficients; serial changes (1–30 cycles) in the level of oxy-Hb associated with cycle repetition in the various regions were also evaluated using Spearman’s rank correlation coefficients. These correlation coefficients tested for associations between the number of task cycles and changes in oxy-Hb level for each region.

## Results

**Fig 3:**
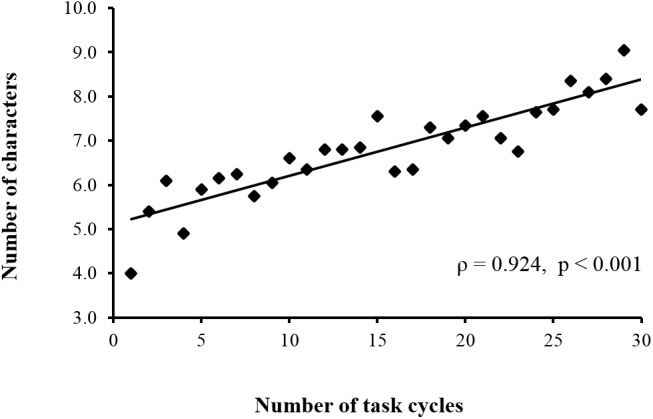
Correlation between performance (number of correctly entered characters minus incorrectly entered characters) and number of task cycles. Performance significantly increased with cycle repetitions.

**Fig 4:**
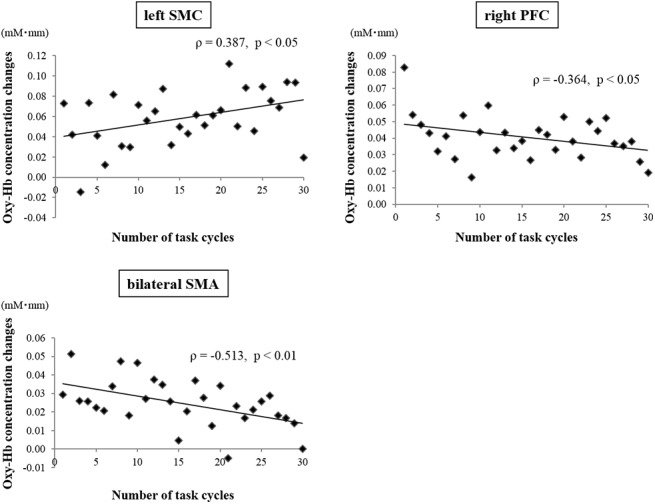
Correlations between oxy-Hb changes over three regions and number of task cycles. Vertical axis represents mean oxy-Hb concentration changes (in mM*mm). Left SMC activation significantly increased, whereas SMA and right PFC activation significantly decreased with cycle repetition.

Fig 3 shows the average performance (i.e., number of correct entries minus incorrect entries into the touch-screen terminal) during the touch-screen task over 30 cycles. There was a highly significant positive correlation between task cycle and performance (ρ = 0.924, p < 0.001) (S2 Dataset). Fig 4 shows the mean changes in oxy-Hb concentration for three cortical regions over the 30 cycles of the touch-screen task. A significant positive correlation between task cycle and oxy-Hb concentration for the channels covering the area of the left SMC (ρ = 0.387, p < 0.05) was observed (S3 Dataset). In contrast, significant negative correlations between task cycle and oxy-Hb concentration for the channels covering the SMA (ρ = -0.513, p < 0.01) (S4 Dataset) and right PFC (ρ = -0.364, p < 0.05) (S5 Dataset) were obtained. There were no significant correlations between task cycle and oxy-Hb changes for the channels covering the other areas (left PFC, preSMA, bilateral PMC, and right SMC) (S6 Dataset).

## Discussion

Flick input is one of the entry methods for Japanese characters in a touch-screen terminal. However, in general, flick input operation is difficult. The user of the touch-screen terminal has to learn to input a letter quickly to effectively convey information. Because the subjects of this study were inexperienced in touch-screen terminal operation, this task was equivalent to motor learning combined with complex cognitive processing. In recent years, activities involving complex cognitive processing during operation of touch-screen terminals have become abundant in everyday life. However, the cerebral blood flow dynamics during motor learning with complex cognitive processing in humans are not well understood. Therefore, we used NIRS to examine the cerebral blood flow dynamics of various cortical regions during motor learning of flick input operation of a touch-screen terminal.

### Role of SMC in motor learning

The left SMC was activated with an increasing number of task cycles, whereas the right SMC did not show such an activation pattern. The left SMC is equivalent to a region including primary motor and primary sensory areas primarily controlling operation of the hand [46]. PET and NIRS studies have reported that the cerebral blood flow volume of the contralateral primary motor area of the operating hand increased with the frequency of finger tapping [26,47,48]. In addition, the primary motor and primary sensory areas contralateral to the operating hand became activated during motor learning of the finger. In the present study, the number of character entries significantly increased with the task cycle. During the course of one experiment, motor learning of the finger occurred in all subjects, as confirmed by the improvement of the flick input operation. Therefore, we speculate that increases in the momentum of the finger reflect motor learning.

### Role of SMA in motor learning

The SMA extracts motor programs that depend on memory information and the initiation of spontaneous movements. Thus, the SMA plays an important role in situations where a compound movement is controlled [49–53]. The examination of SMA activity during motor learning can be accomplished by various neuroimaging techniques. However, there is little consensus on the activity of the SMA during motor learning [54–60]. In a previous NIRS study, SMA activation gradually increased with motor learning [38]. That study used a pursuit rotor task requiring simple motor learning and therefore differs from the motor learning task of the current study that involved complex cognitive processing. Although the SMA showed greatly increased activity immediately after experiment initiation, its activity was gradually attenuated with increasing task cycles.

We suggest that these changes in cerebral blood flow were caused by motor inhibition. All subjects of this study owned a feature phone and inputted characters using ten keys that operated in a toggle manner. During the experiment, all subjects were required to input letters using a method that differed from the operation they had mastered previously. We speculate that the two divergent character input methods competed, leading to motor inhibition that occurred in all subjects during this task. Preliminary research has suggested that the SMA is activated by motor inhibition [61,62]. We further suggest that the SMA is activated initially by motor inhibition; as learning progresses, SMA activity is attenuated. Although our findings demonstrate activation of SMA by motor inhibition, we could not find similar reports that examined changes of cerebral blood flow showing motor inhibition with cognitive learning over time. Therefore, this study may be the first to report cerebral blood flow dynamics in SMA with cognitive learning involving motor inhibition. However, the task required complex cognitive processes including visuomotor adaptation and working memory. Therefore, the current task was not specifically designed to test motor inhibition. In future experiments, we thus need to examine the dynamics of cerebral blood flow over the SMA during more specific tasks requiring motor inhibition.

This may be the first study to report the activity of the SMA during a motor learning task with complex cognitive processing using character entry into a touch-screen terminal over time. Therefore, further research is needed to examine the role of SMA during motor learning using other complex tasks (e.g., character input on a personal computer, trail making, serial reaction time, or other visuomotor adaptation and motor sequence learning tasks that involve complex cognitive processing).

### Role of the PFC in motor learning

The PFC is an important neural substrate for visual working memory [63]. As the task of this study consisted of entering a letter presented on a PC screen into a hand-held touch-screen terminal, working memory was required to successfully accomplish this task. Memory encoding has been associated with lateral PFC activation across a variety of experimental paradigms in functional neuroimaging [64,65]. In the current study, the right PFC activation was gradually attenuated with increasing task cycle. However, activation of the left PFC continued without attenuation as the task cycles progressed. Activity of PFC regions has been reported to gradually decrease with learning [66–71]. Therefore, acquisition of the cognitive skill associated with working memory in our subjects may have attenuated the activity of the right PFC. As all subjects in our study were right-handed, we assume that their left hemisphere was language-dominant. Thus, the left PFC was likely involved in linguistic information manipulation [63,72,73] and continued to be recruited during all task cycles, thereby maintaining the activity of the left PFC. Previous studies have reported PFC asymmetry for memory encoding of verbal and nonverbal code [74,75]. The right PFC is activated during a nonverbal task, whereas the left PFC is activated during a verbal task, supporting the validity of our hypothesis.

### Role of other regions

The PMC is involved in choice and control of movements that depend on sensory information [76,77]. During the 1–30 cycles, the PMC continued to be activated bilaterally. As the task of this study consisted of a visuomotor problem, we speculate that the activity of PMC was maintained because the subjects depended on visual information throughout all task cycles. The preSMA controls the aspect of the task that uses the rich entry from the premotor area, including order decision of the complex movements during preparation and movement choice; this also includes motor learning of changes in movement style and timing of the movement initiation. Furthermore, the preSMA is activated by cognitive control in the absence of movement control [78–82]. The activity of the preSMA was previously reported to be attenuated with motor learning [38]. However, in this study, the activity of preSMA continued during all task cycles.

The subjects performed a movement control problem with complex cognitive information processing by translating the letter presented on a monitor to the movement direction of their finger. We suggest that this step represents early stages of cognitive-motor learning. In the current task, sustained attentional engagement from the subjects was required during cognitive processing, and we speculate that this led to the continued activity of preSMA without attenuation.

### Limitations of this study

First, as our measurements included only the initial learning period of 30 cycles, it was not possible to determine how cerebral blood flow dynamics might have changed after completion of the 30-cycle learning stage. Therefore, further studies are needed to investigate cerebral blood flow dynamics after task training.

Second, we used the number of characters entered minus the number of incorrect entries as the performance index in this study, and investigated its relationship with Oxy-Hb concentration changes in each region in this study,. However, it is also possible that the Oxy-Hb concentration changes for each region would show a different course if the speed of entering the characters and reaction time (i.e. the time to enter a character appearing on a monitor upon confirmation) were investigated separately as a performance index. Further studies should be performed to address this issue.

Third, activation in deeper structures such as the basal ganglia, which are closely linked with the frontal cortices, cannot be detected because of the technical limitations of NIRS. We thus could not address functional connectivity between brain areas, but it would be interesting to investigate functional connectivity between the right PFC and SMA during complex motor learning tasks. In future research, it will be necessary to consider other neuroimaging techniques that measure cerebral blood flow dynamics in humans during motor learning of flick input operation of a touch-screen terminal.

## Conclusions

We examined cerebral blood flow dynamics during motor learning of flick input into a touch-screen terminal using NIRS. The number of character inputs significantly increased with repetition of task cycles. These results show that motor learning occurred in all subjects during the course of one experiment. In the left SMC, SMA, and right PFC, there was a significant change of cerebral blood flow dynamics as the task cycles progressed, indicating motor learning over time. Changes in activity over the left SMC, SMA, and right PFC likely reflect distinct aspects of acquisition of the motor task such as increase in finger momentum, motor inhibition, and visual working memory, respectively.

## Supporting Information

S1 Dataset Subjects information. (XLSX)

S2 Dataset Correlations between task performance and task cycles. (XLSX)

S3 Dataset Correlations between left SMC Oxy-Hb concentration changes and number of task cycles. (XLSX)

S4 Dataset Correlations between SMA Oxy-Hb concentration changes and number of task cycles. (XLSX)

S5 Dataset Correlations between right PFC Oxy-Hb concentration changes and number of task cycles. (XLSX)

S6 Dataset Correlations between other regions Oxy-Hb concentration changes and number of task cycles. (XLSX)

S1 Supporting Information Presentation for the task. (PPTX)

## Abbreviations

NIRS: near-infrared spectroscopy
PFC: prefrontal cortex
PFC: left dorsolateral prefrontal cortex
preSMA: presupplementary motor area
SMA: supplementary motor area
PMC: dorsal premotor cortex
SMC: sensorimotor cortex
