# Supplementary material for: Changes in Cerebral Hemodynamics during Complex Motor Learning by Character Entry into Touch-Screen Terminals
Source: PLoS One. 2015 Oct 20;10(10):e0140552. doi: 10.1371/journal.pone.0140552 (PMC4618511; doi:10.1371/journal.pone.0140552)
Supplement: S1 Supporting Information — (PPTX) [file pone.0140552.s007.pptx]

## Slide 1
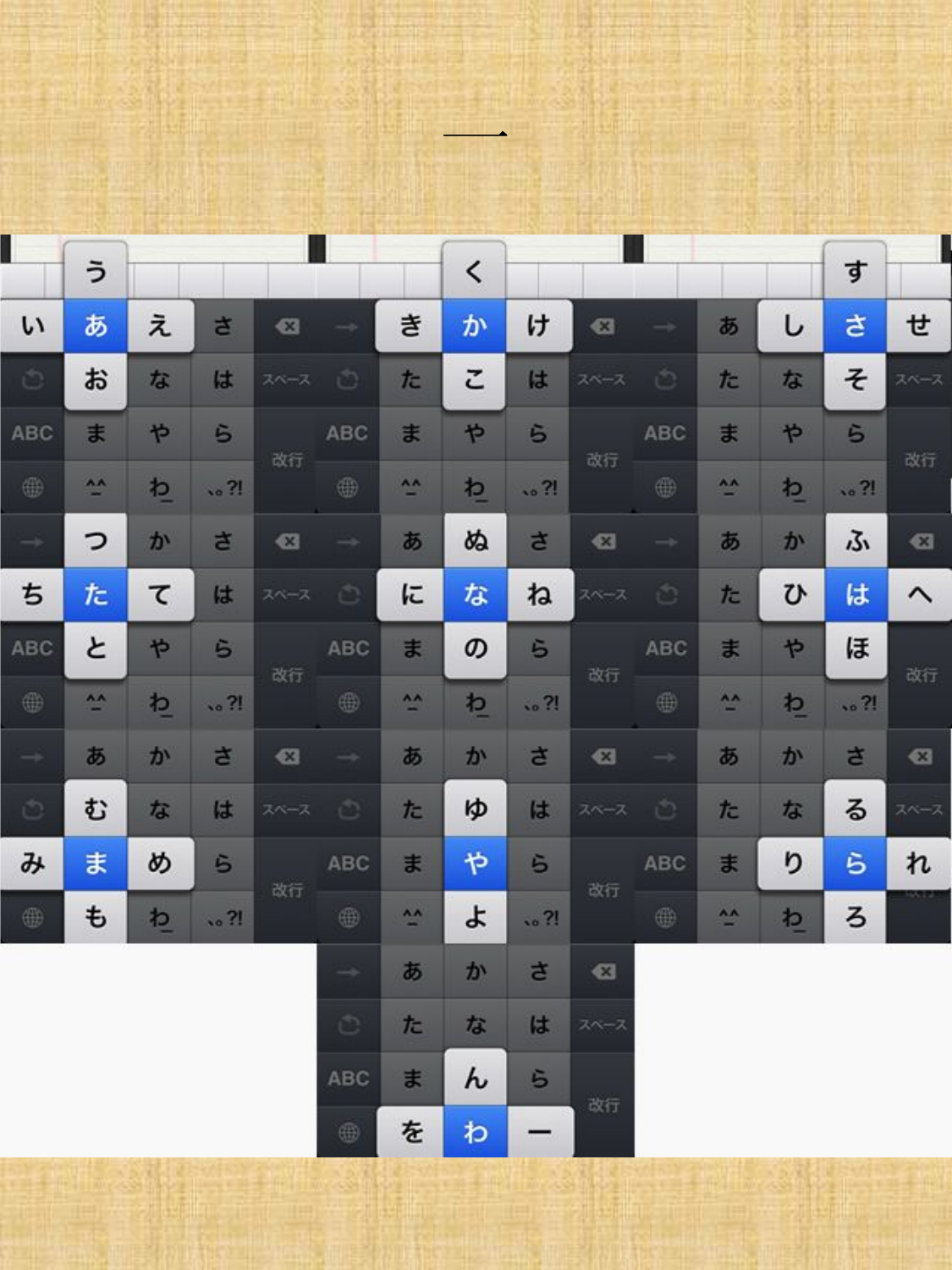

フリック入力一覧表

## Slide 2
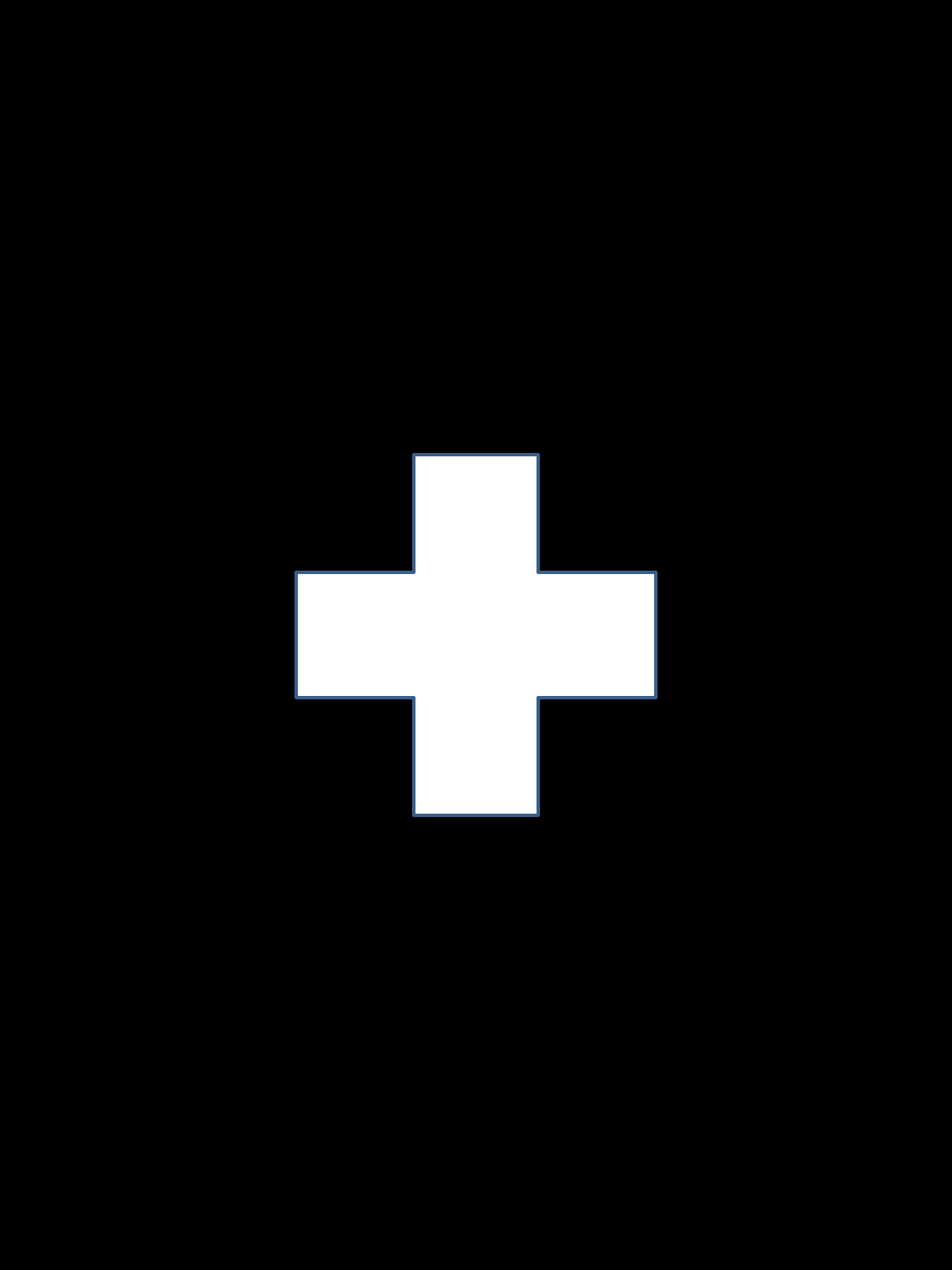

## Slide 3
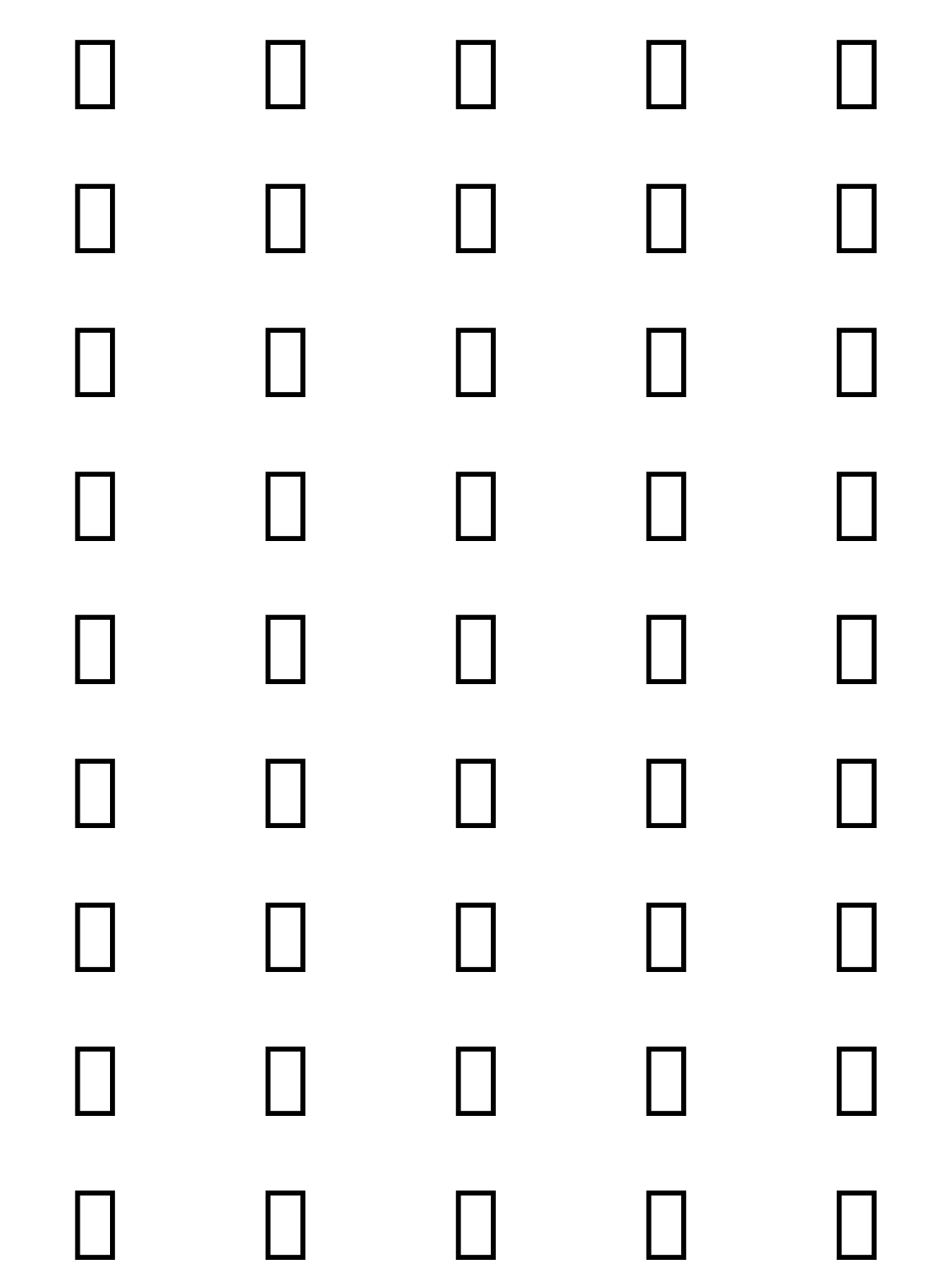

| も | き | こ | ふ | は |
| --- | --- | --- | --- | --- |
| し | み | く | れ | つ |
| え | た | と | を | ぬ |
| け | さ | ち | へ | ね |
| ほ | ま | い | わ | さ |
| て | る | の | め | り |
| お | あ | す | ろ | ゆ |
| や | ら | そ | ひ | む |
| よ | う | な | ん | に |

## Slide 4
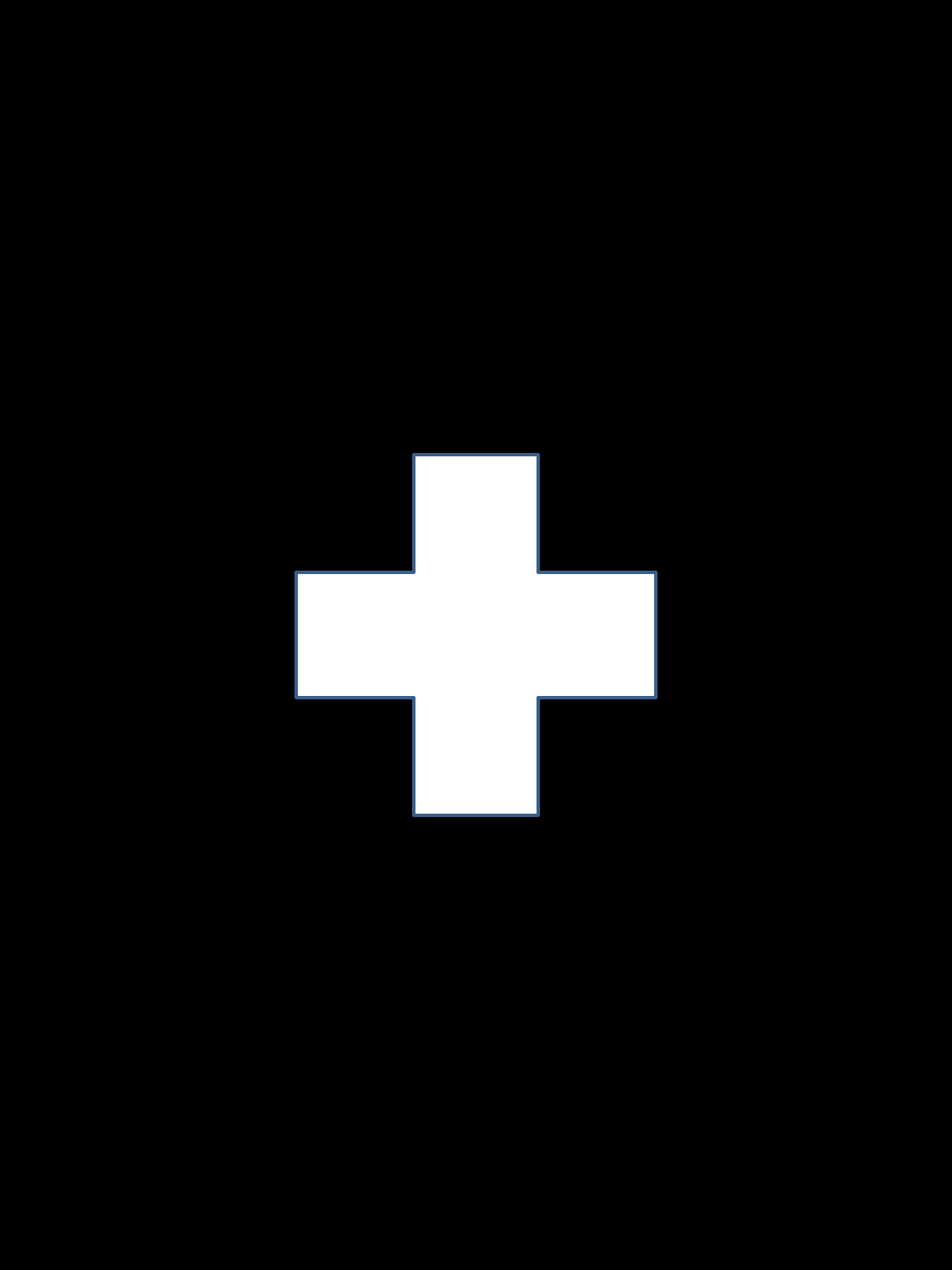

## Slide 5
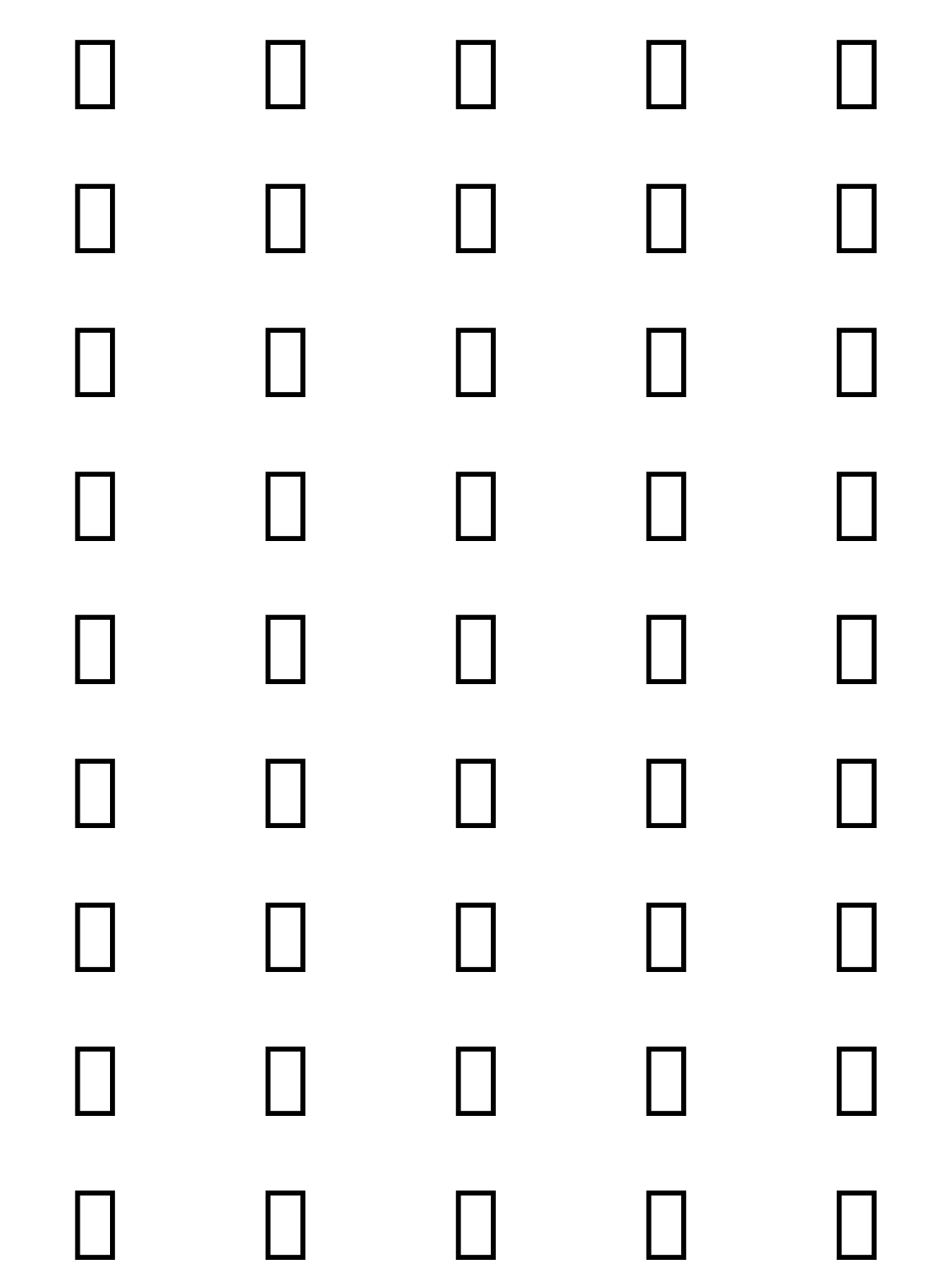

| し | む | は | ゆ | も |
| --- | --- | --- | --- | --- |
| お | と | み | き | た |
| あ | へ | け | ふ | よ |
| や | り | ぬ | を | さ |
| ろ | に | つ | れ | ぬ |
| か | な | ん | く | こ |
| わ | ひ | ほ | そ | い |
| せ | の | ま | る | て |
| え | す | う | ら | め |

## Slide 6
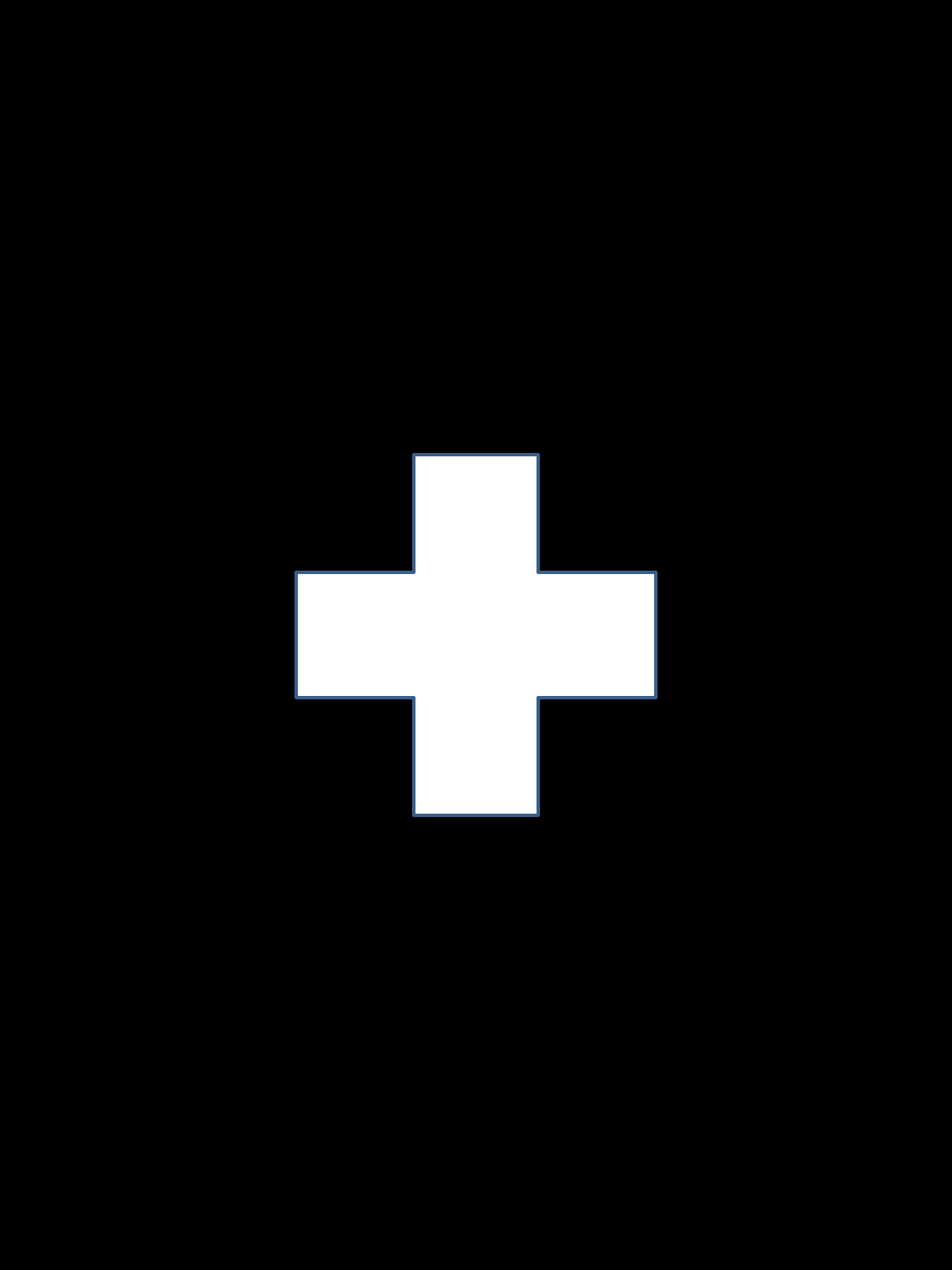

## Slide 7
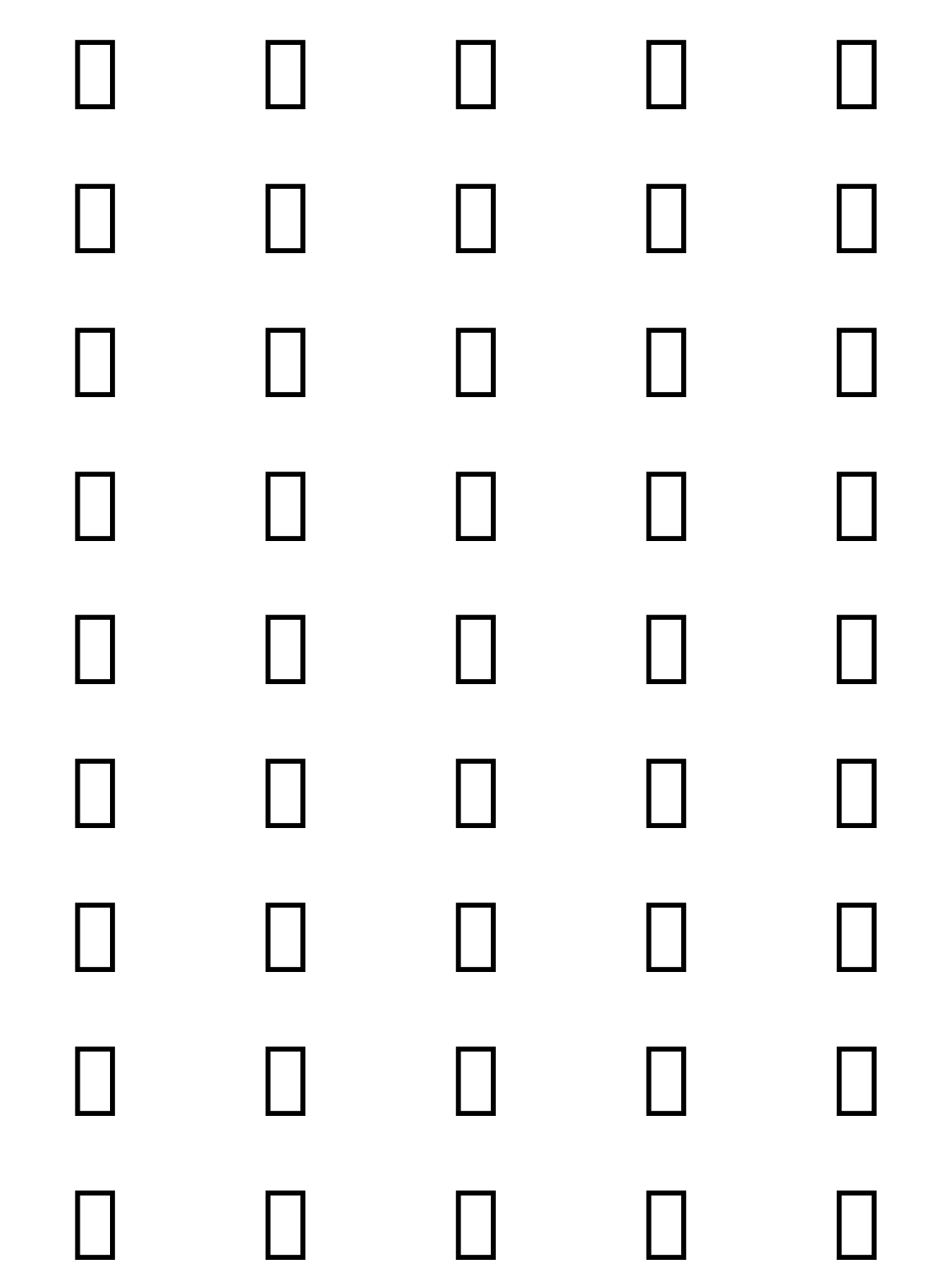

| も | た | よ | さ | ね |
| --- | --- | --- | --- | --- |
| こ | い | て | め | ら |
| る | そ | く | れ | を |
| ふ | き | ゆ | は | み |
| け | ぬ | つ | ん | ほ |
| ま | う | す | の | ひ |
| な | に | り | へ | と |
| む | し | お | あ | や |
| ろ | か | わ | せ | え |

## Slide 8
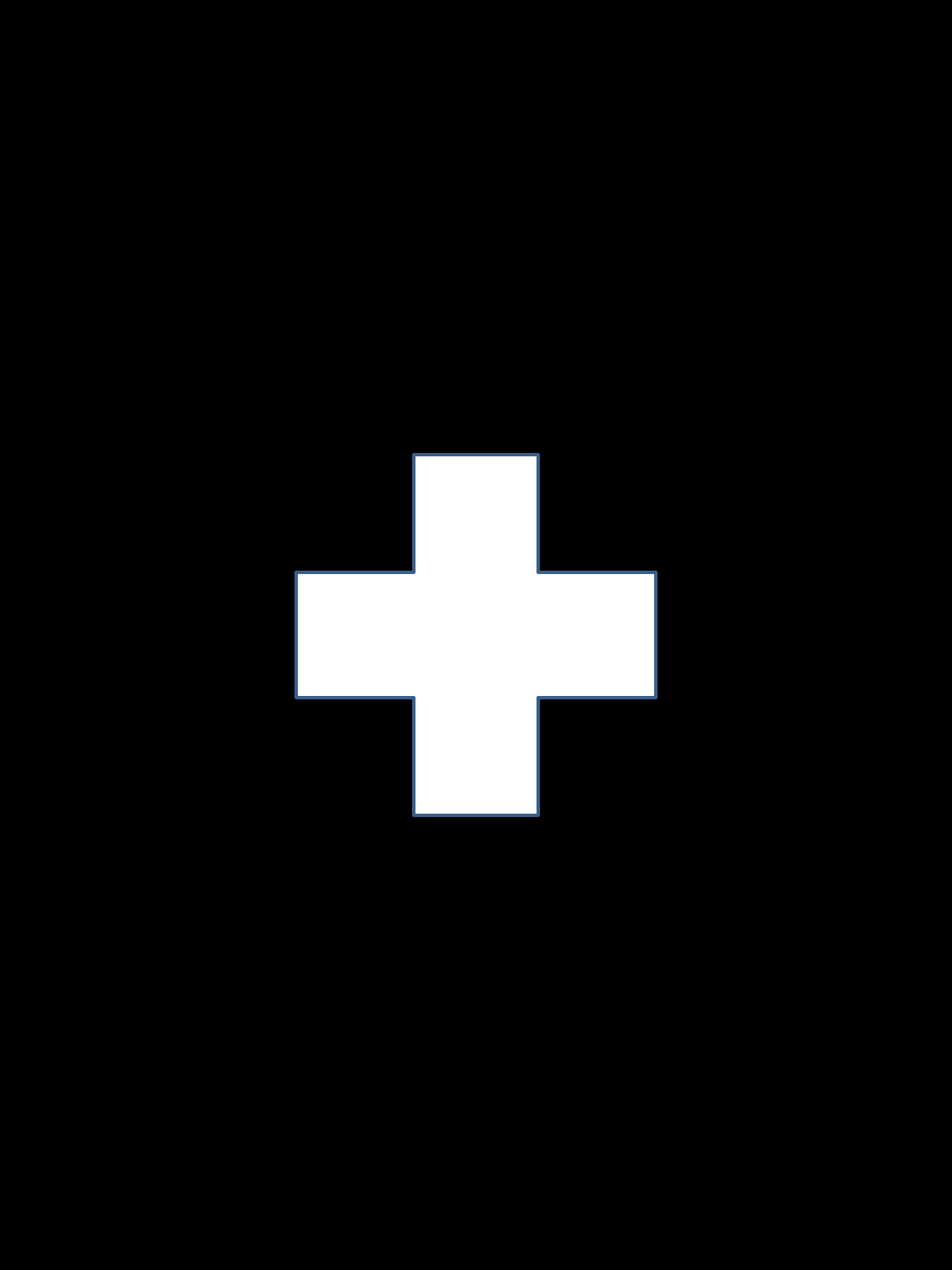

## Slide 9
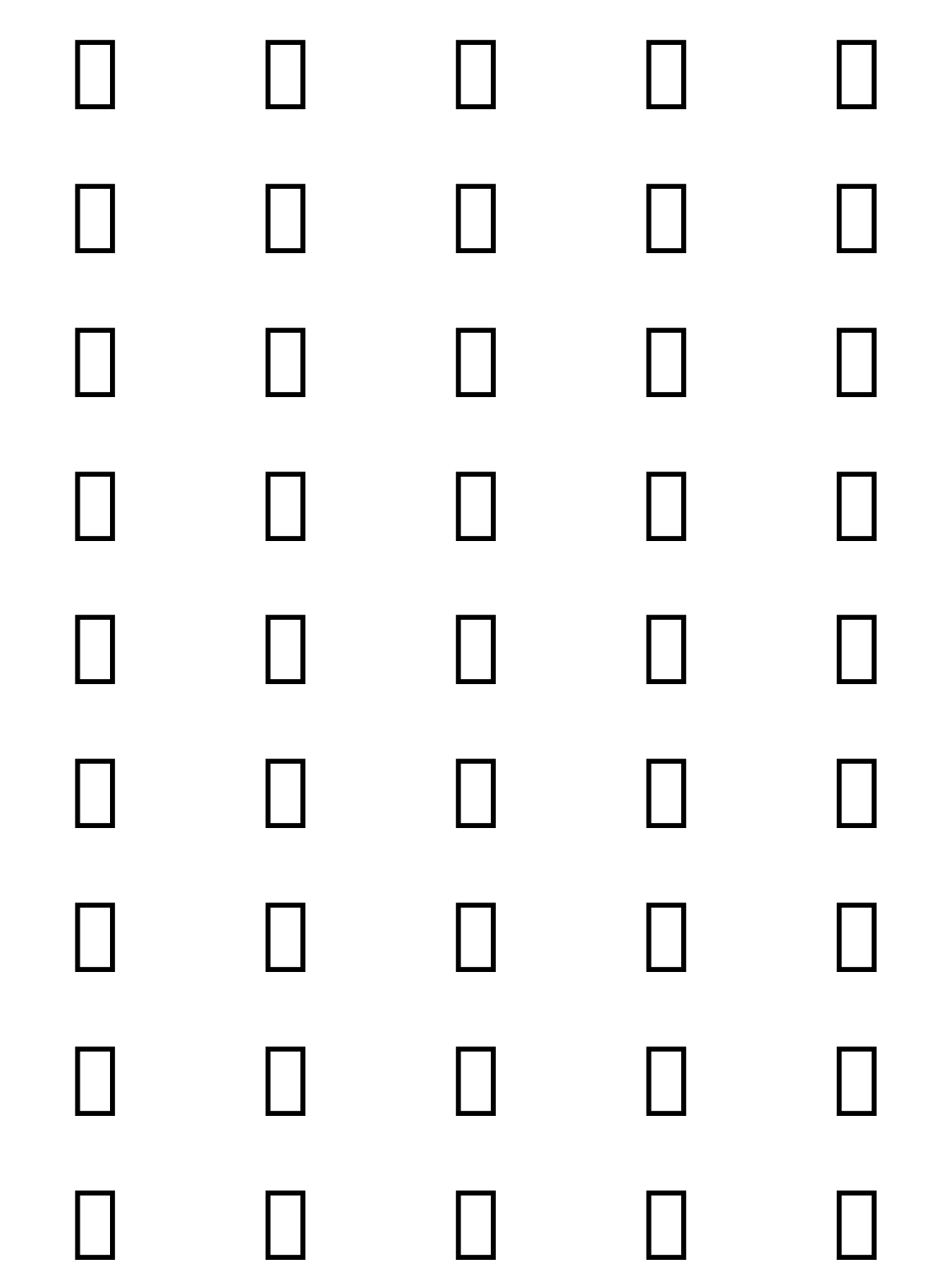

| ぬ | ら | う | す | え |
| --- | --- | --- | --- | --- |
| へ | る | ま | の | せ |
| い | そ | ほ | ひ | わ |
| こ | く | ん | な | か |
| ね | れ | つ | に | ろ |
| さ | を | ぬ | り | や |
| よ | ふ | け | へ | あ |
| た | き | み | と | お |
| も | ゆ | は | む | し |

## Slide 10
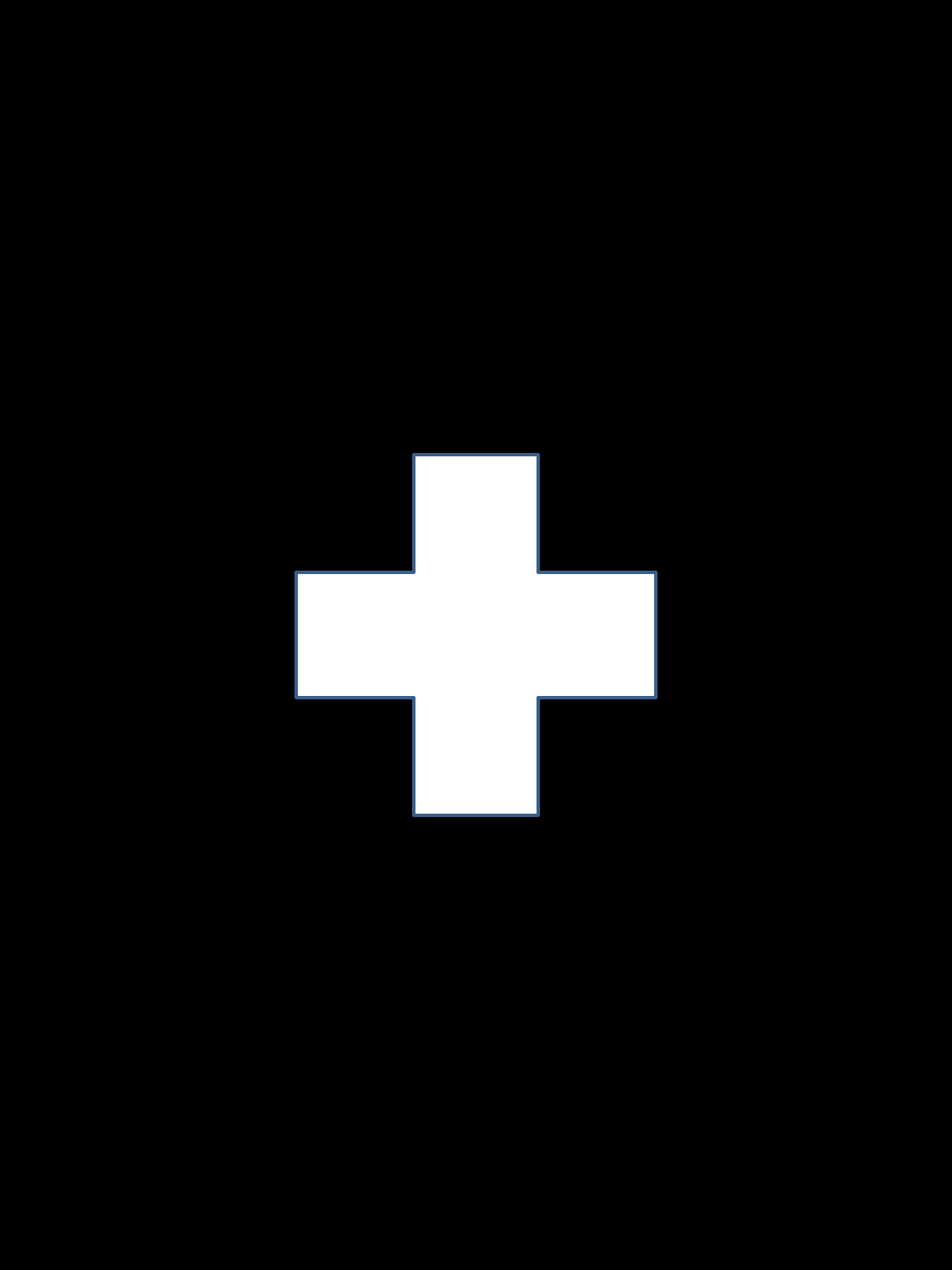

## Slide 11
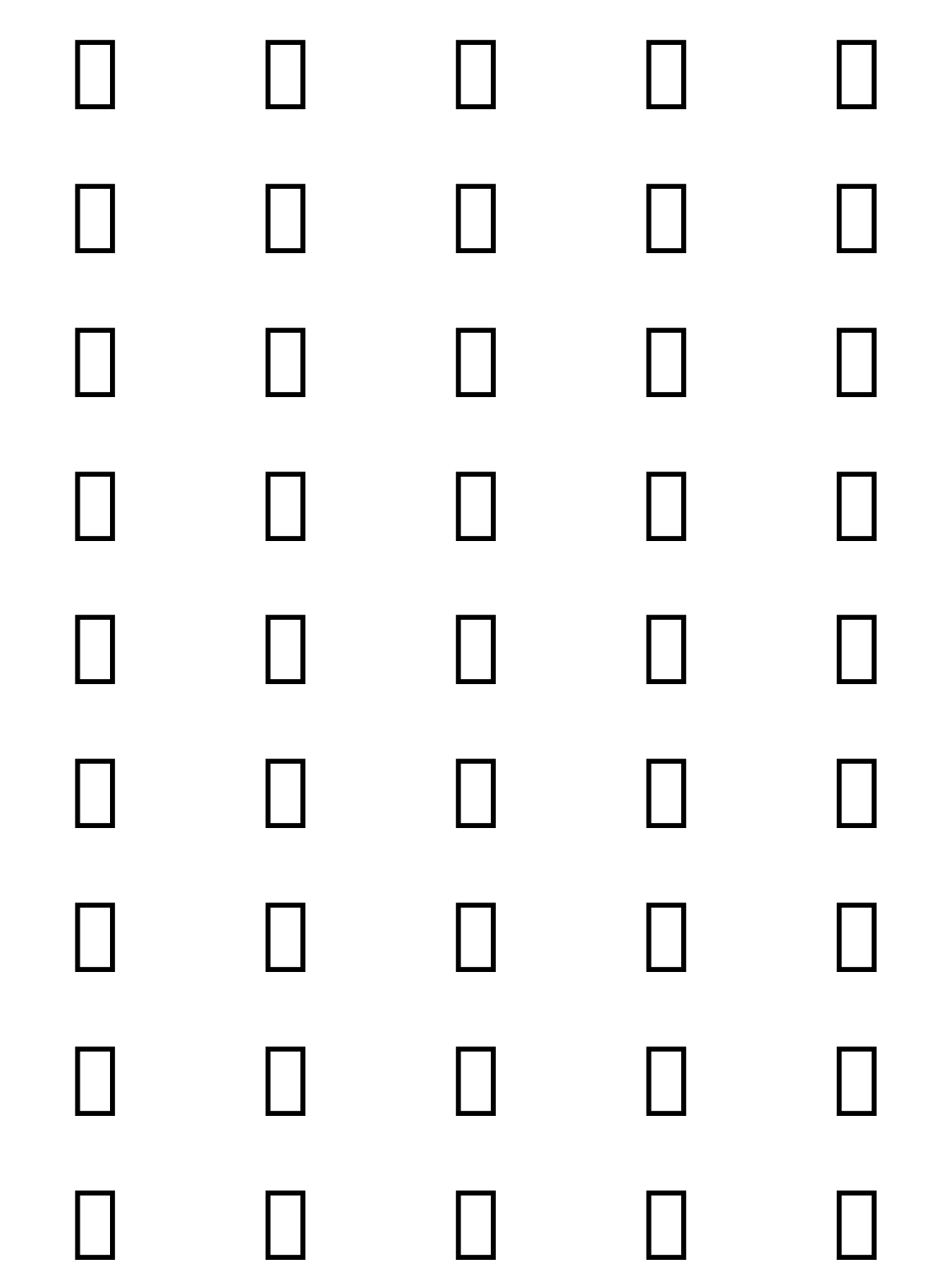

| せ | ま | れ | ん | ら |
| --- | --- | --- | --- | --- |
| て | ほ | る | す | あ |
| わ | の | え | つ | う |
| な | へ | は | や | ろ |
| か | く | り | を | み |
| ち | も | ひ | し | む |
| に | け | め | こ | ね |
| ぬ | ゆ | お | い | た |
| と | き | そ | よ | ふ |

## Slide 12
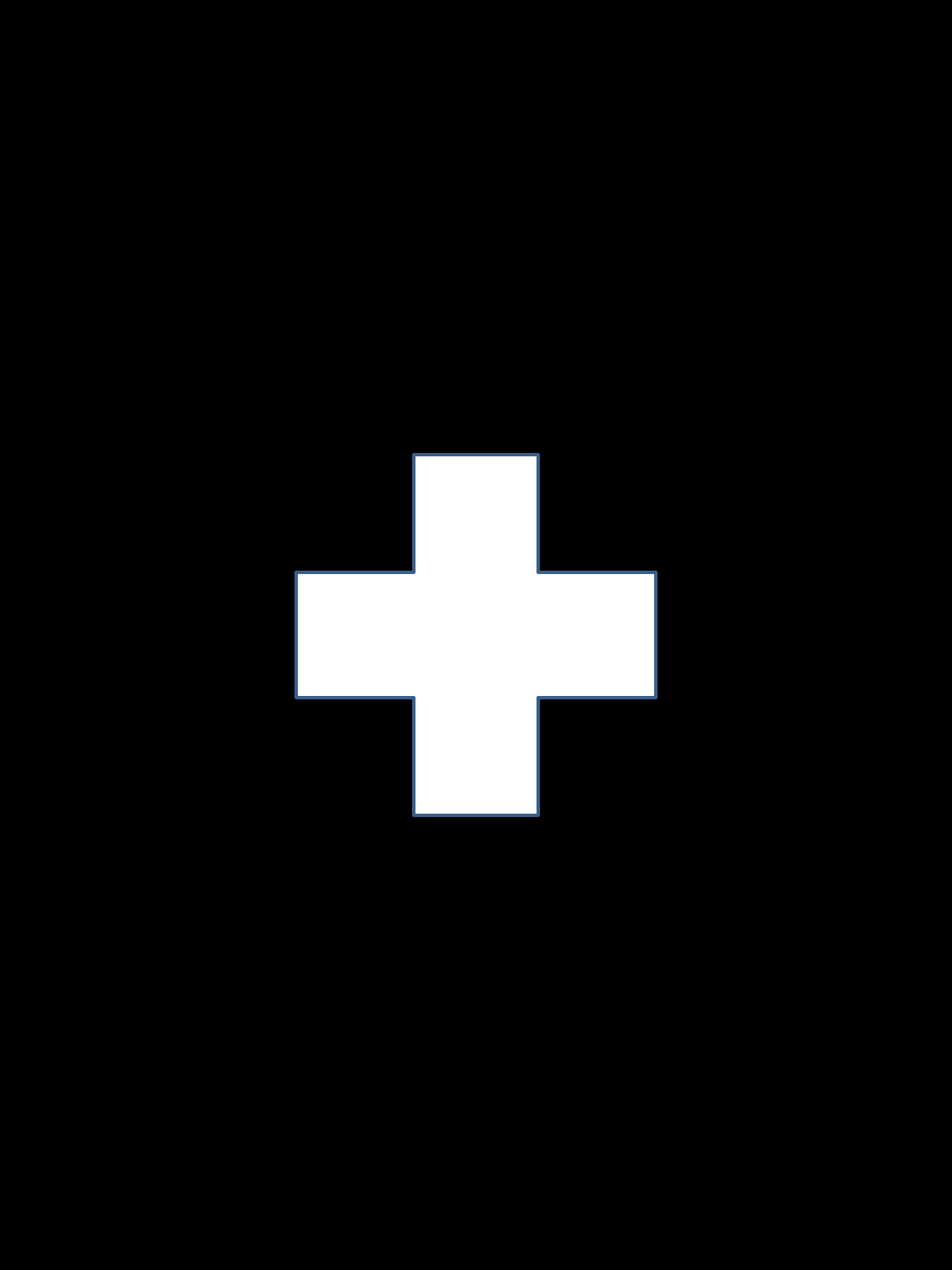

## Slide 13
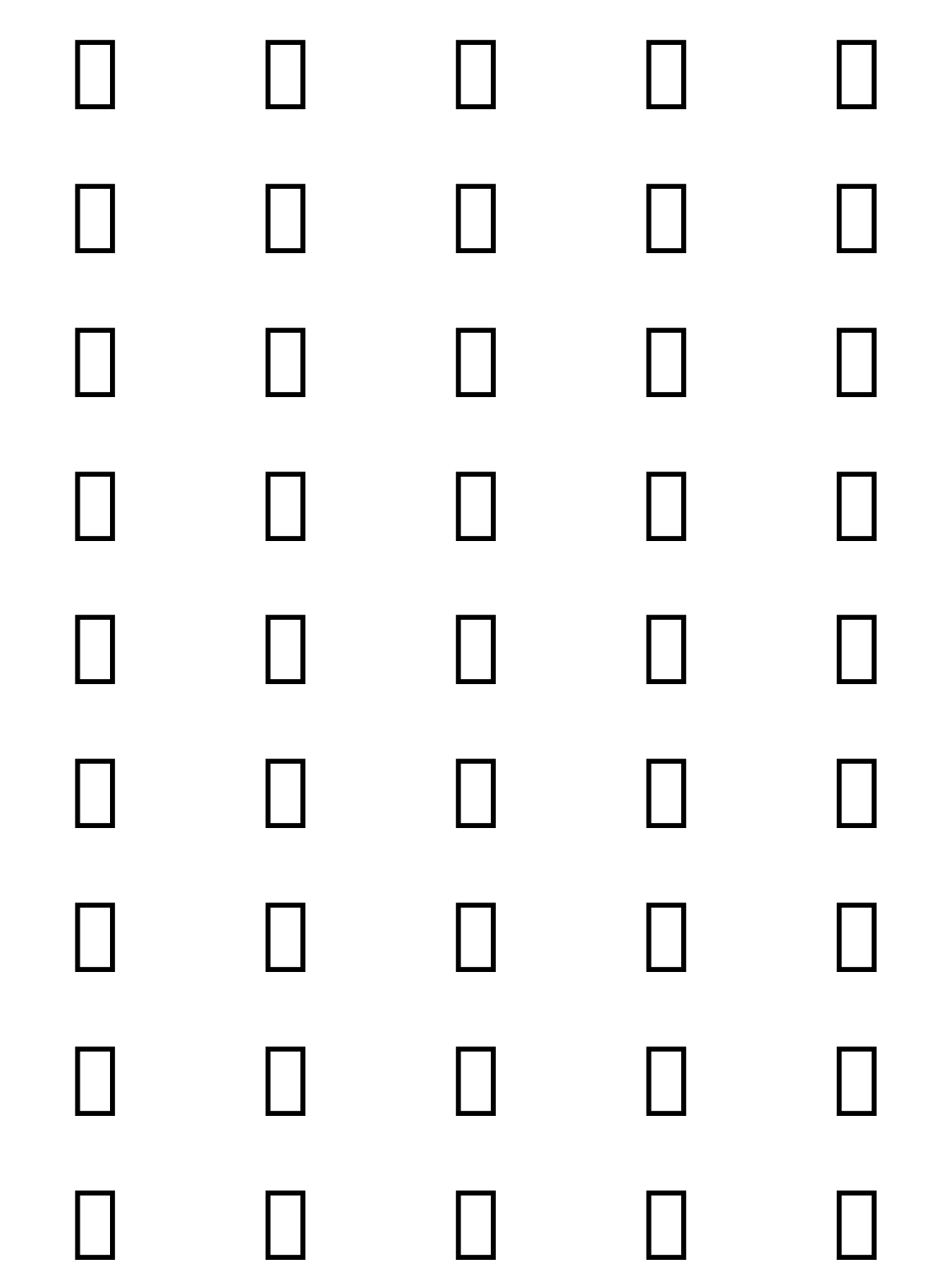

| い | ね | こ | め | け |
| --- | --- | --- | --- | --- |
| あ | わ | な | か | ち |
| す | の | て | く | も |
| る | え | は | り | ひ |
| は | つ | や | を | し |
| へ | う | ろ | み | む |
| せ | ん | ら | ぬ | に |
| さ | れ | ま | と | ゆ |
| よ | そ | ふ | き | お |

## Slide 14
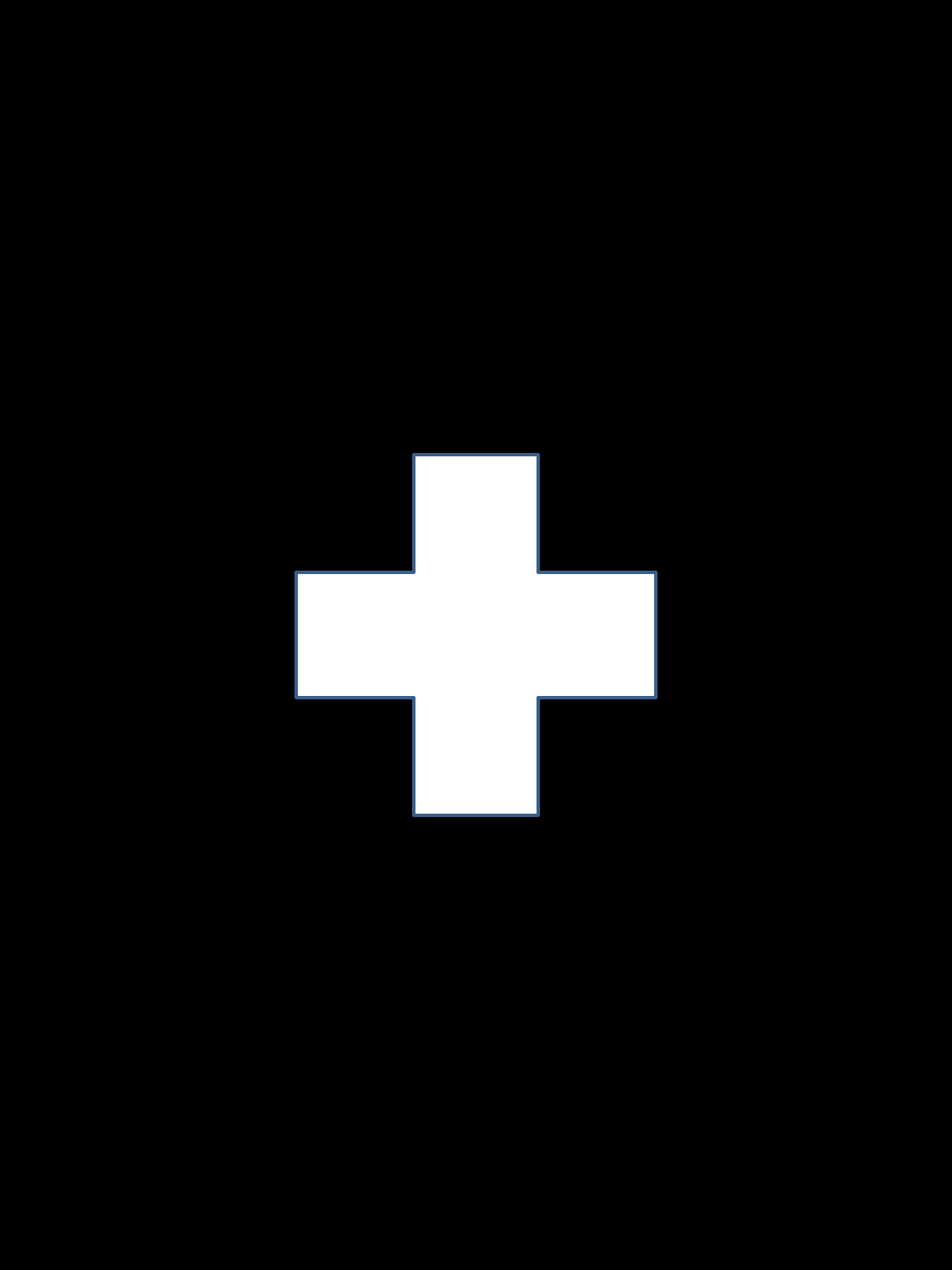

## Slide 15
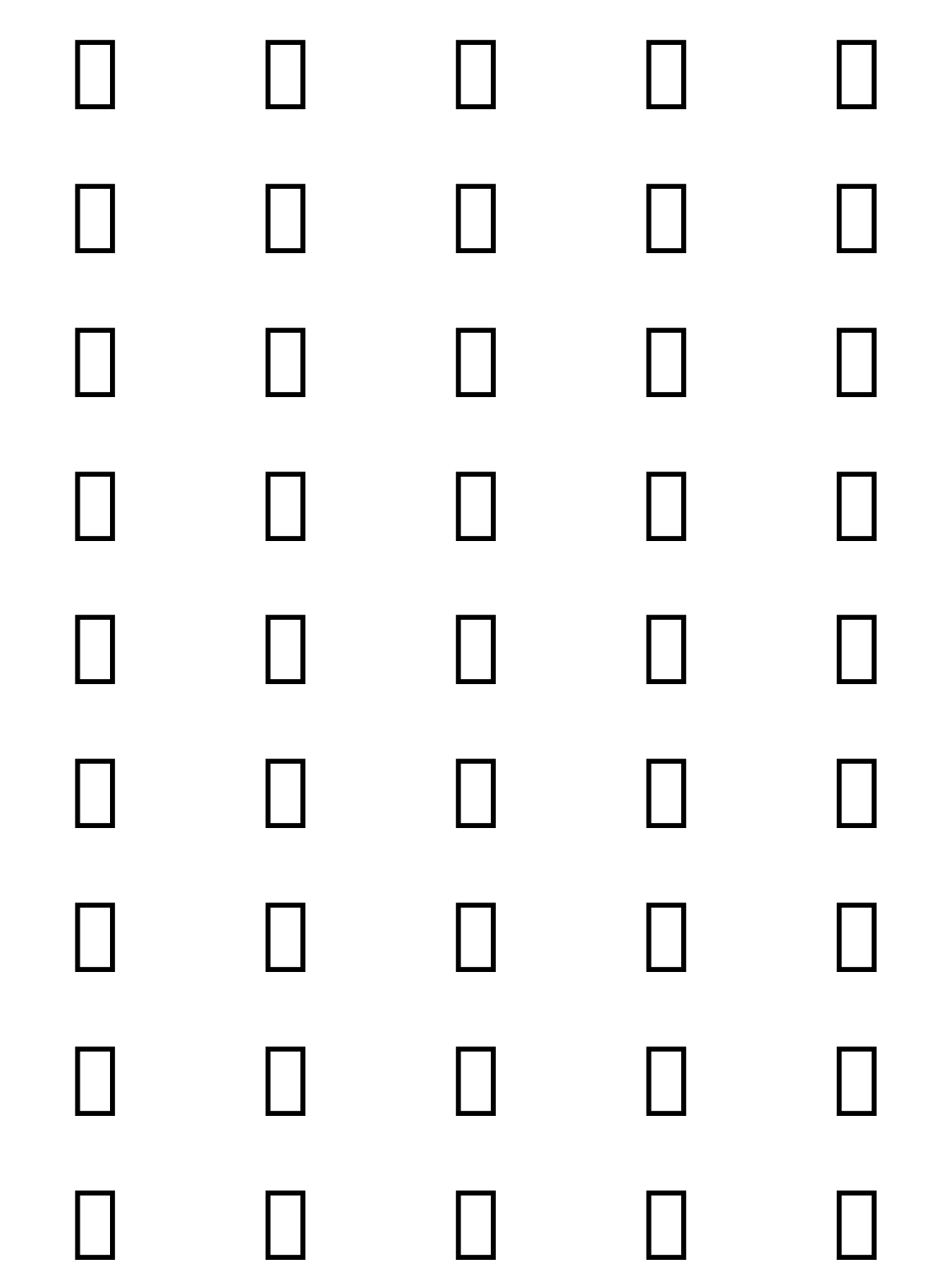

| け | め | こ | ね | い |
| --- | --- | --- | --- | --- |
| よ | ち | か | な | あ |
| も | く | へ | の | す |
| ひ | り | は | え | る |
| し | を | や | つ | ほ |
| む | み | ろ | う | て |
| に | ぬ | ら | ん | せ |
| ゆ | と | ま | れ | さ |
| お | き | ふ | そ | よ |

## Slide 16
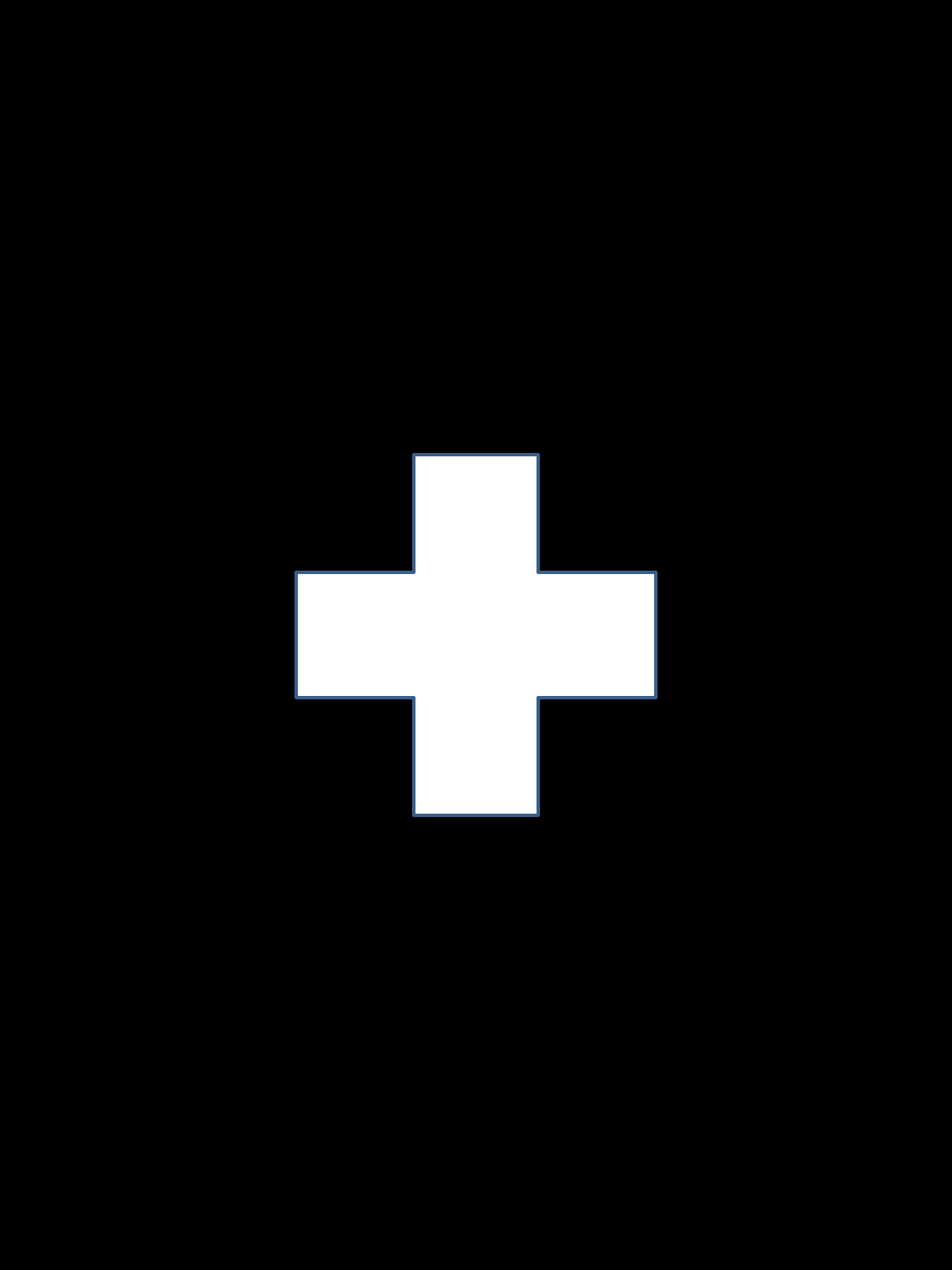

## Slide 17
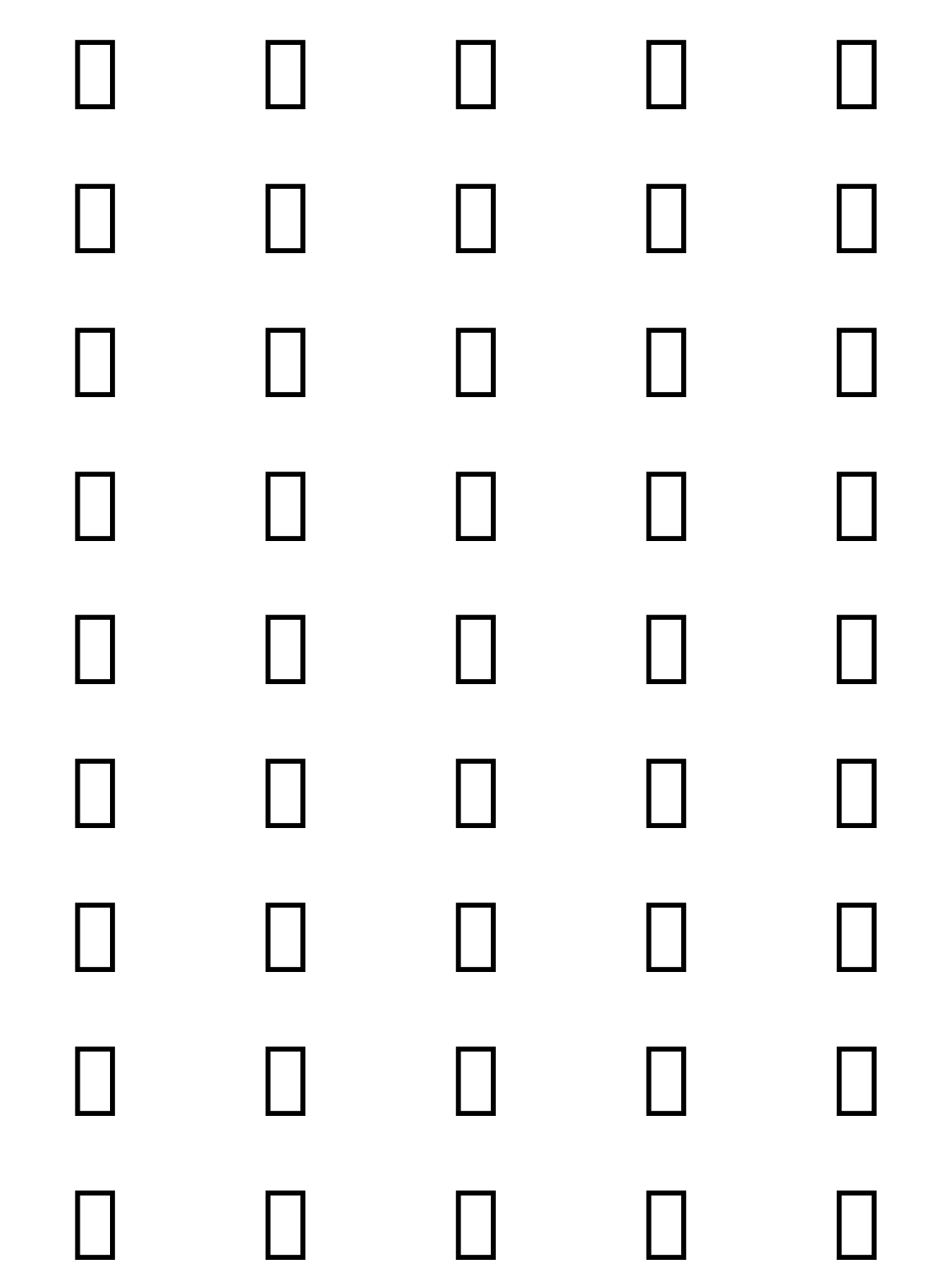

| け | ち | も | ひ | し |
| --- | --- | --- | --- | --- |
| む | に | ゆ | お | た |
| き | と | ぬ | み | を |
| り | く | か | め | こ |
| な | て | は | や | ろ |
| ら | ま | ふ | そ | れ |
| ん | う | つ | え | の |
| わ | ね | い | あ | す |
| る | ほ | て | せ | さ |

## Slide 18
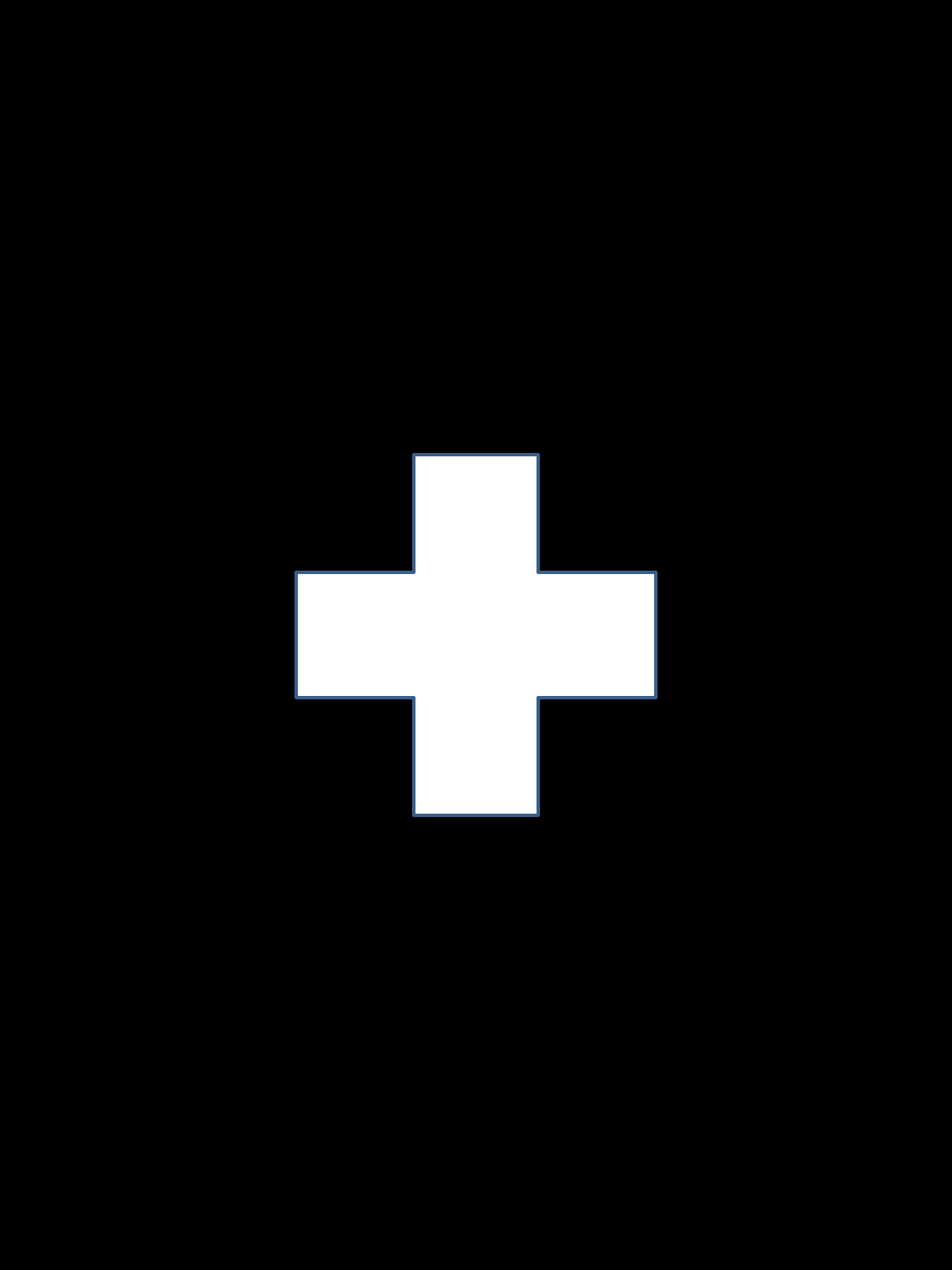

## Slide 19
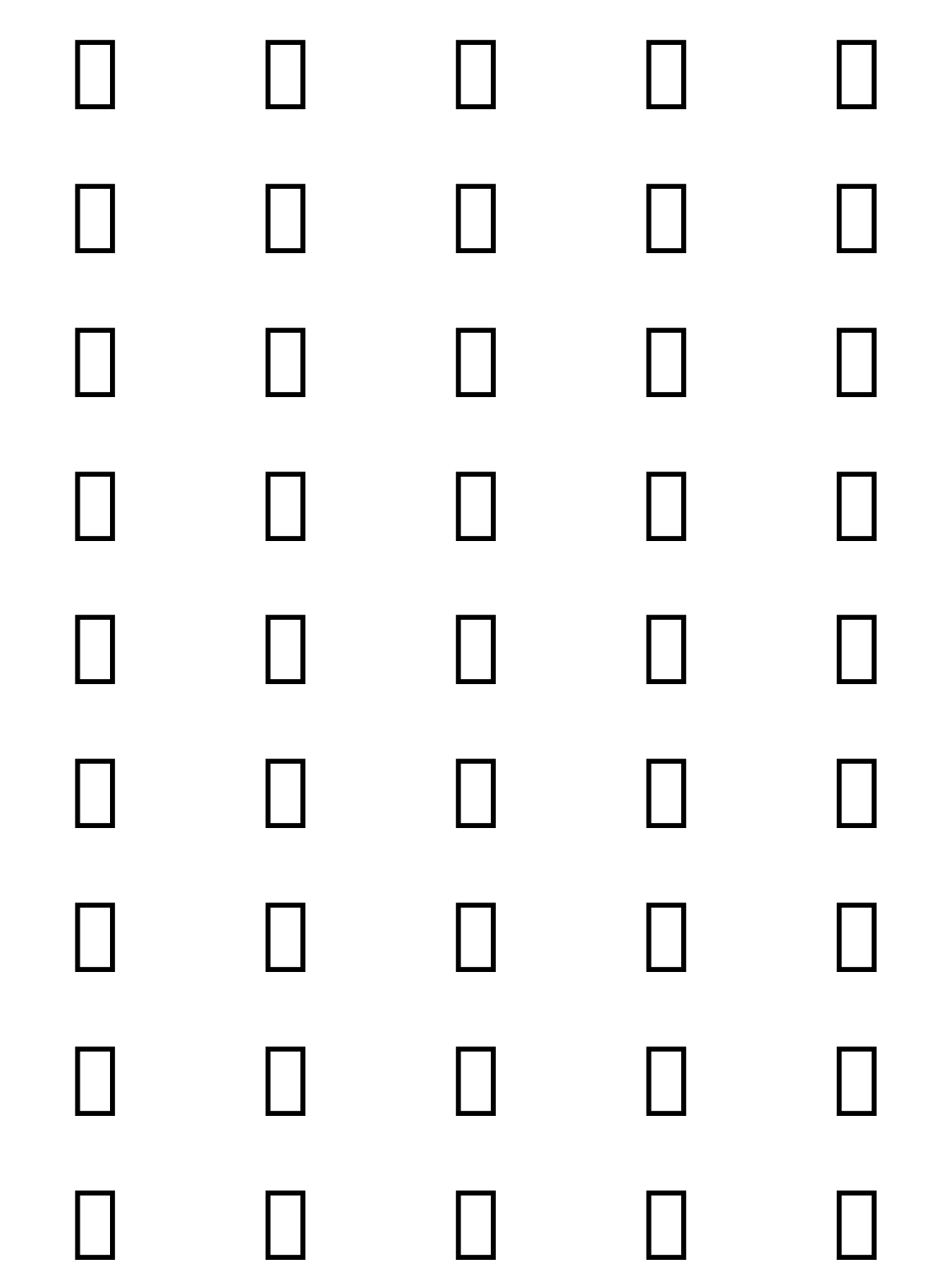

| あ | ろ | そ | よ | し |
| --- | --- | --- | --- | --- |
| の | る | を | ふ | む |
| ゆ | お | か | せ | に |
| て | わ | ま | ん | た |
| け | へ | す | ほ | え |
| つ | や | さ | い | は |
| な | き | く | う | り |
| め | も | と | こ | ひ |
| れ | み | ち | ぬ | ね |

## Slide 20
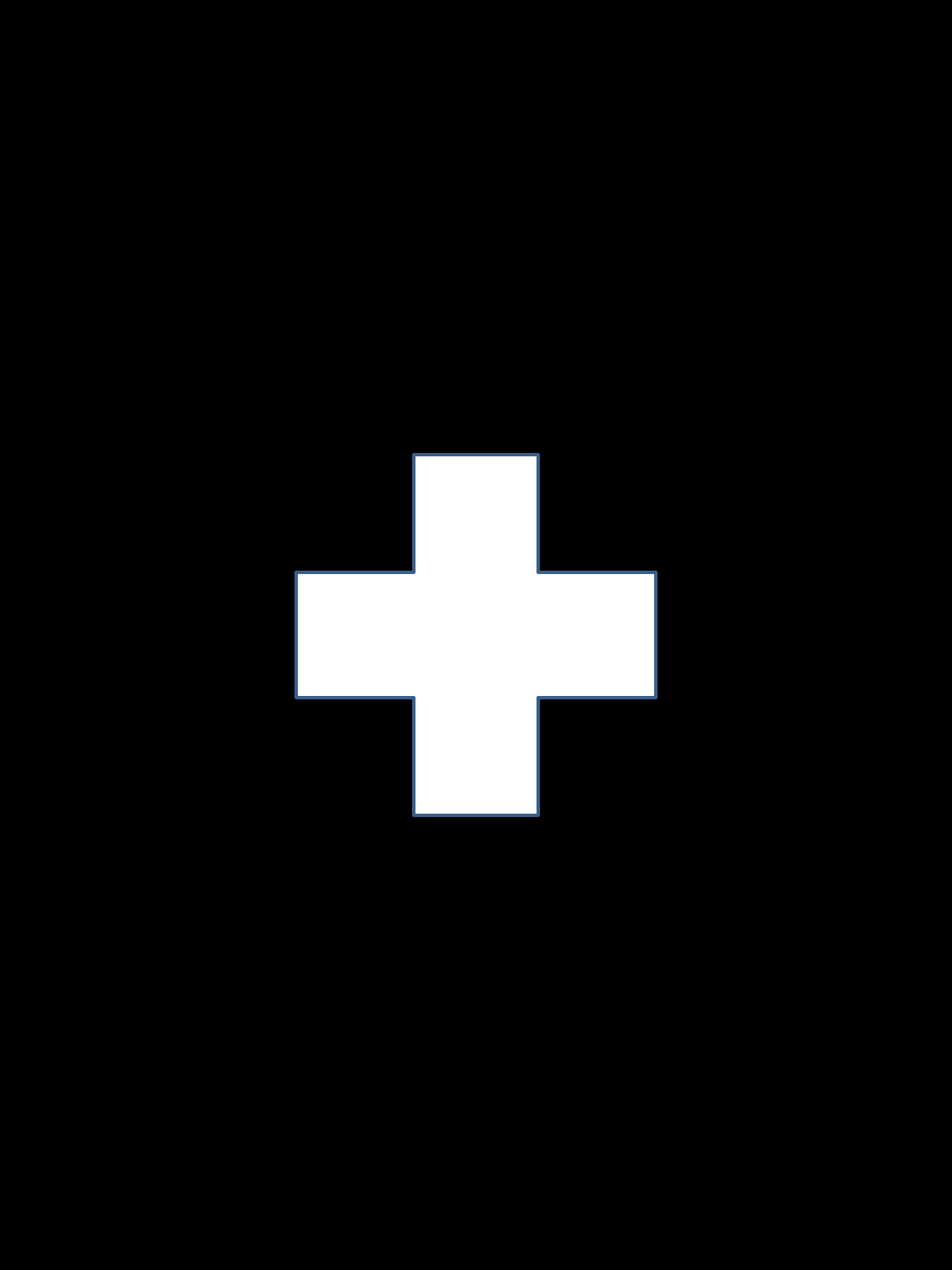

## Slide 21
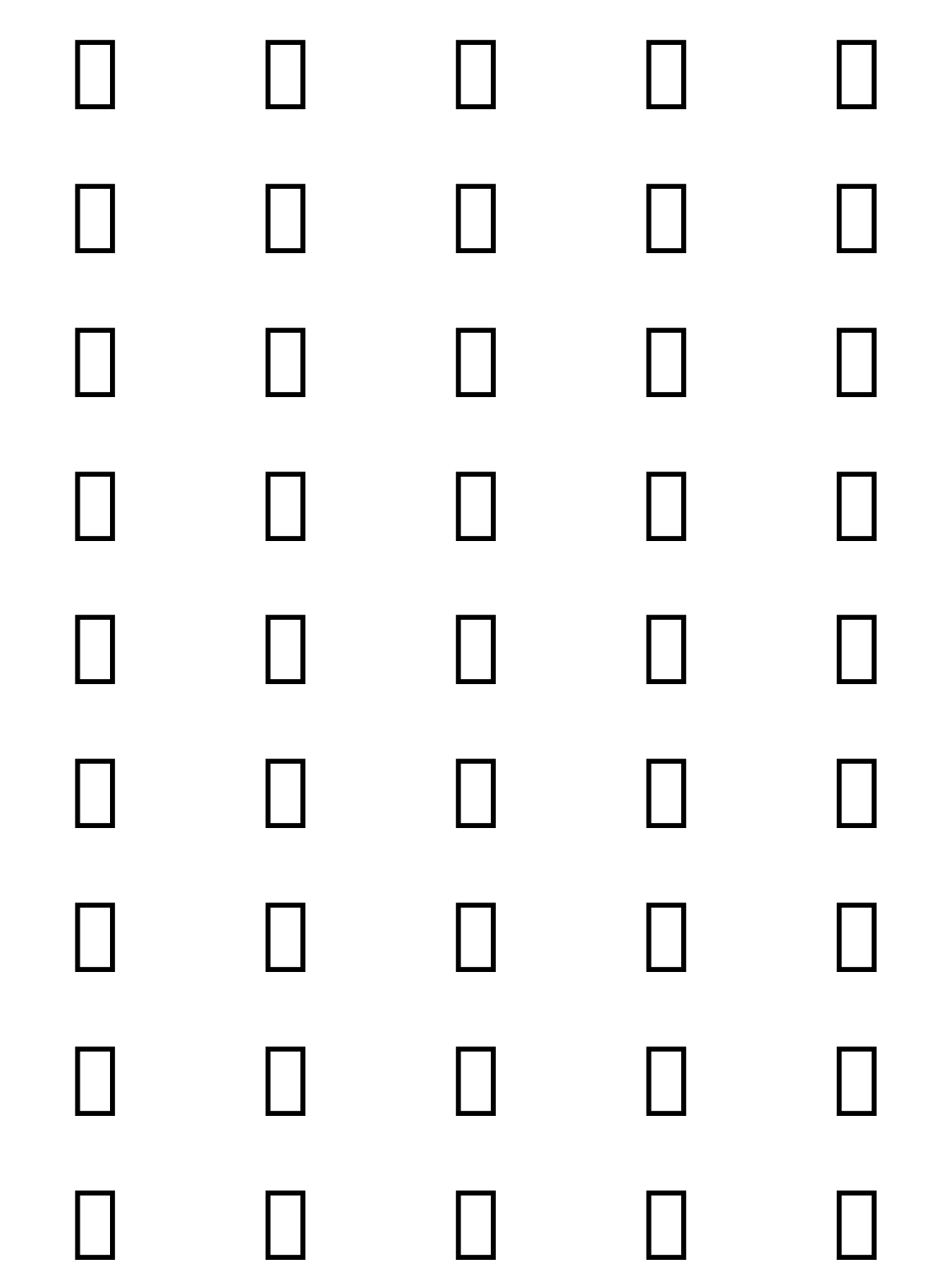

| し | む | に | た | え |
| --- | --- | --- | --- | --- |
| は | り | ひ | ね | ぬ |
| こ | う | い | ほ | ん |
| せ | ふ | よ | そ | を |
| か | ま | す | さ | く |
| と | ち | み | も | き |
| や | へ | わ | お | る |
| ろ | ら | あ | の | ゆ |
| て | け | つ | な | め |

## Slide 22
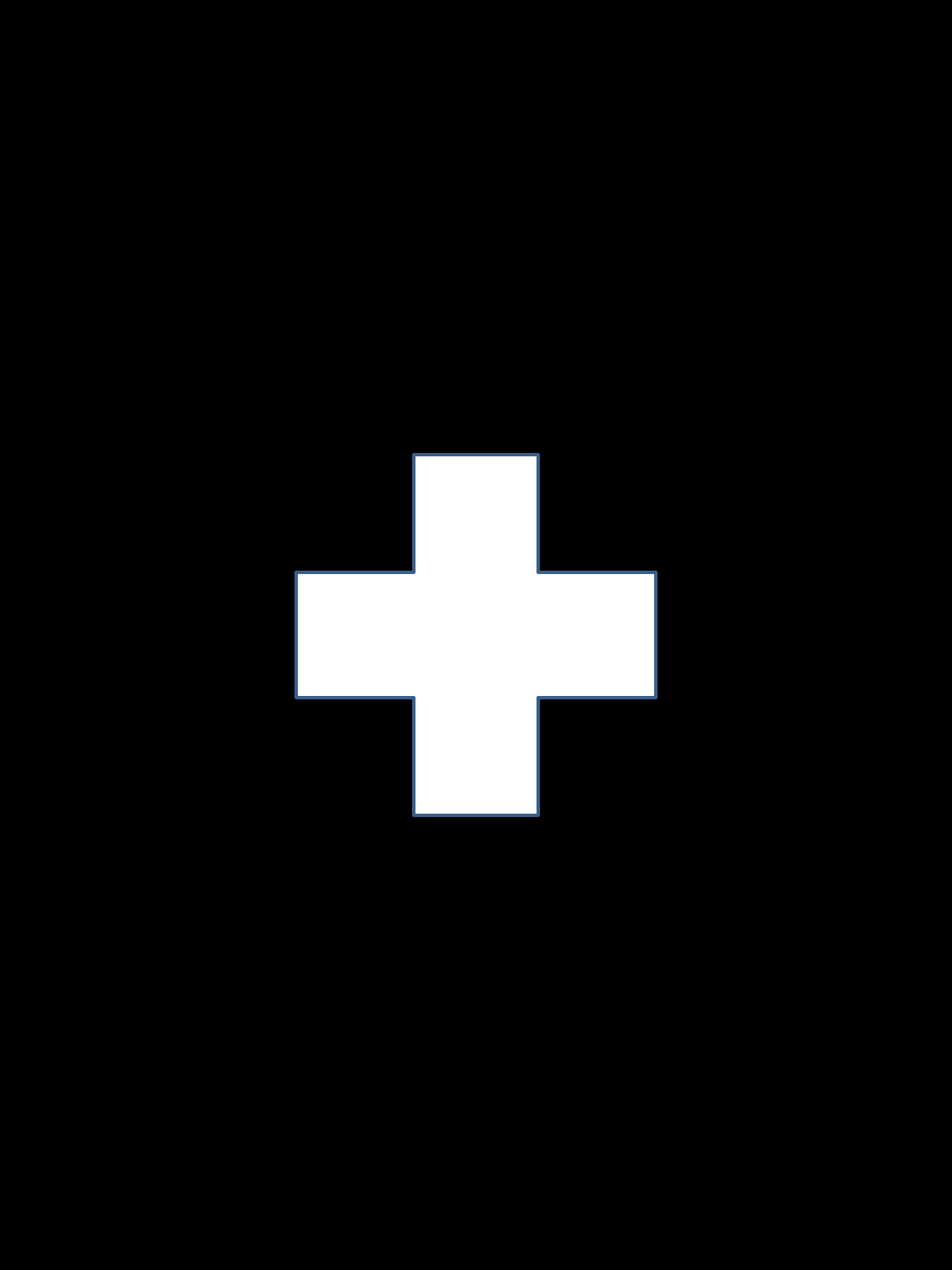

## Slide 23
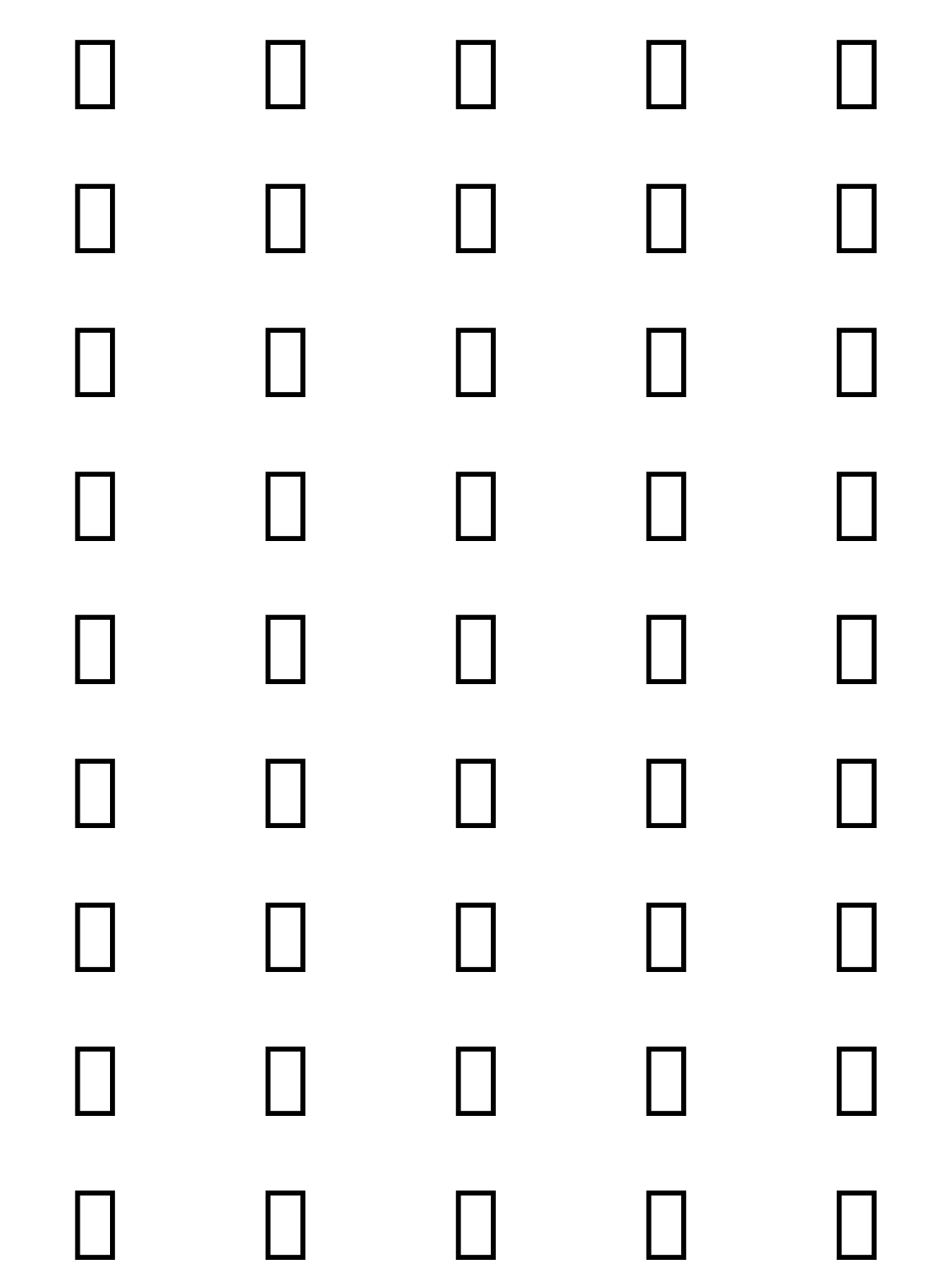

| し | よ | そ | ろ | あ |
| --- | --- | --- | --- | --- |
| む | ふ | を | る | の |
| に | せ | か | お | ゆ |
| た | ん | ま | わ | て |
| え | ほ | す | へ | け |
| は | い | さ | や | つ |
| り | う | く | き | な |
| ひ | こ | と | も | め |
| ね | ぬ | ち | み | れ |

## Slide 24
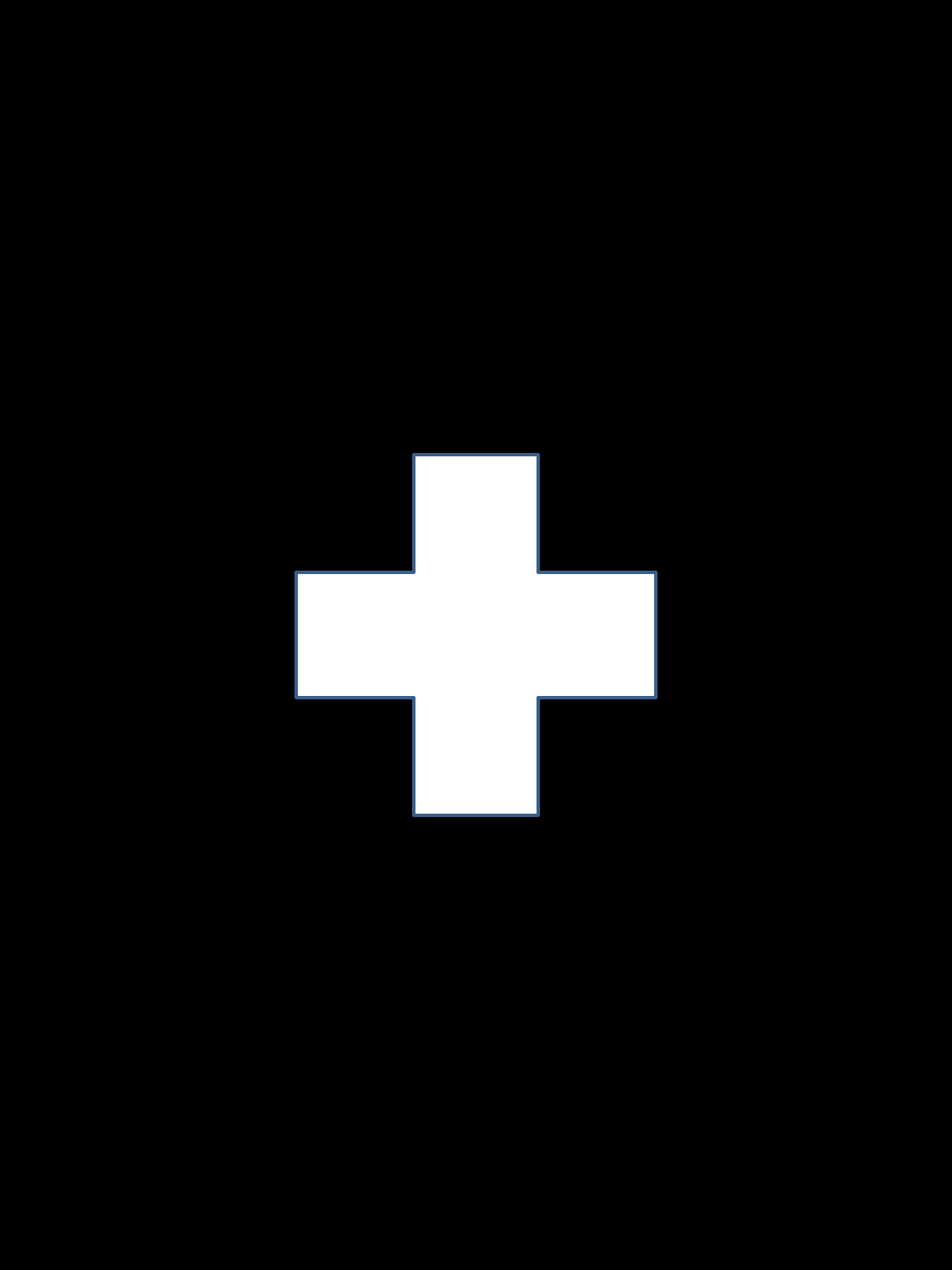

## Slide 25
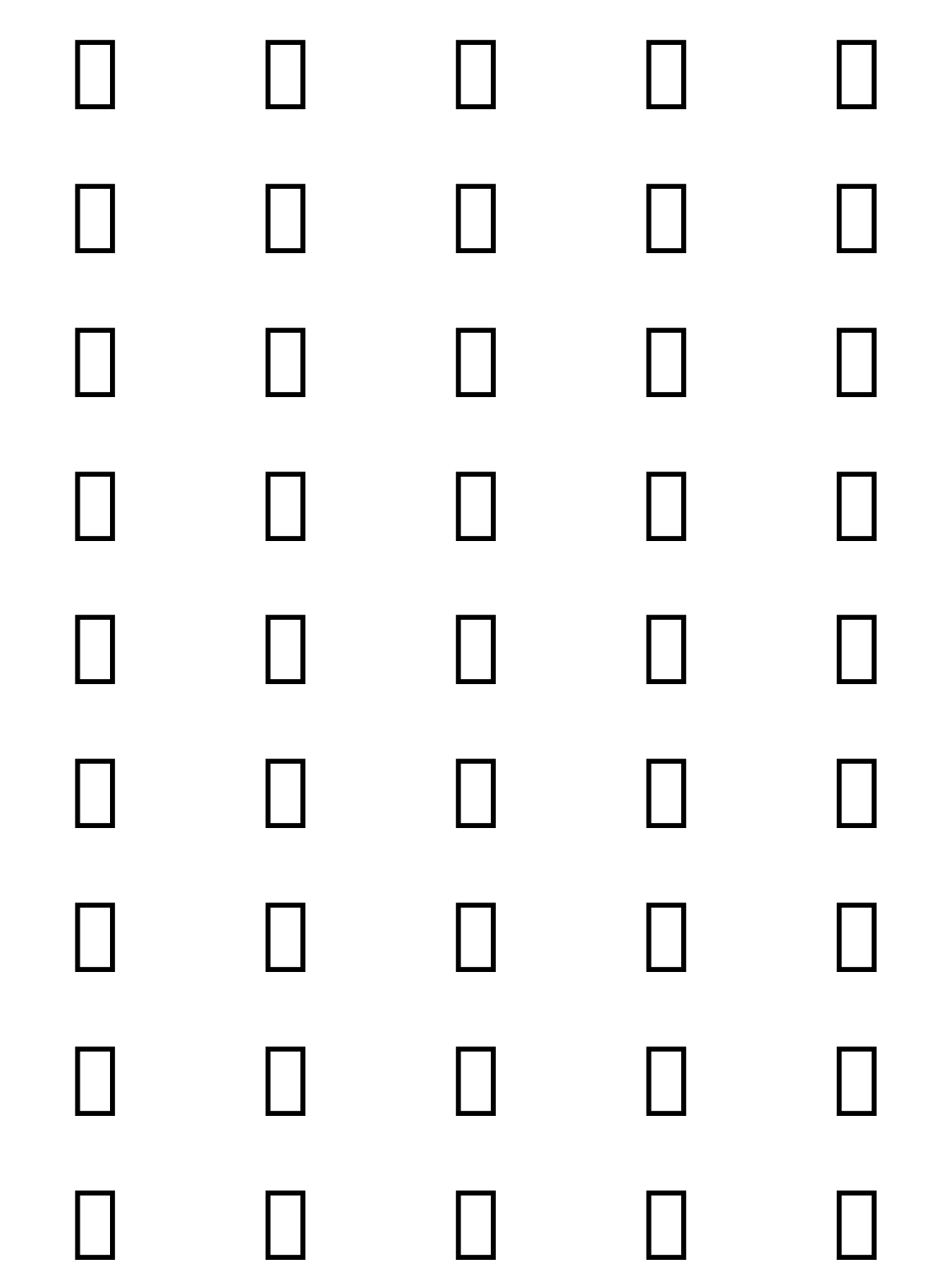

| は | ろ | え | う | の |
| --- | --- | --- | --- | --- |
| ね | そ | あ | お | せ |
| ま | つ | す | む | ん |
| り | か | も | し | い |
| ゆ | を | く | に | や |
| な | ふ | た | ひ | ほ |
| て | わ | る | み | れ |
| ぬ | さ | へ | め | き |
| ち | け | と | こ | ら |

## Slide 26
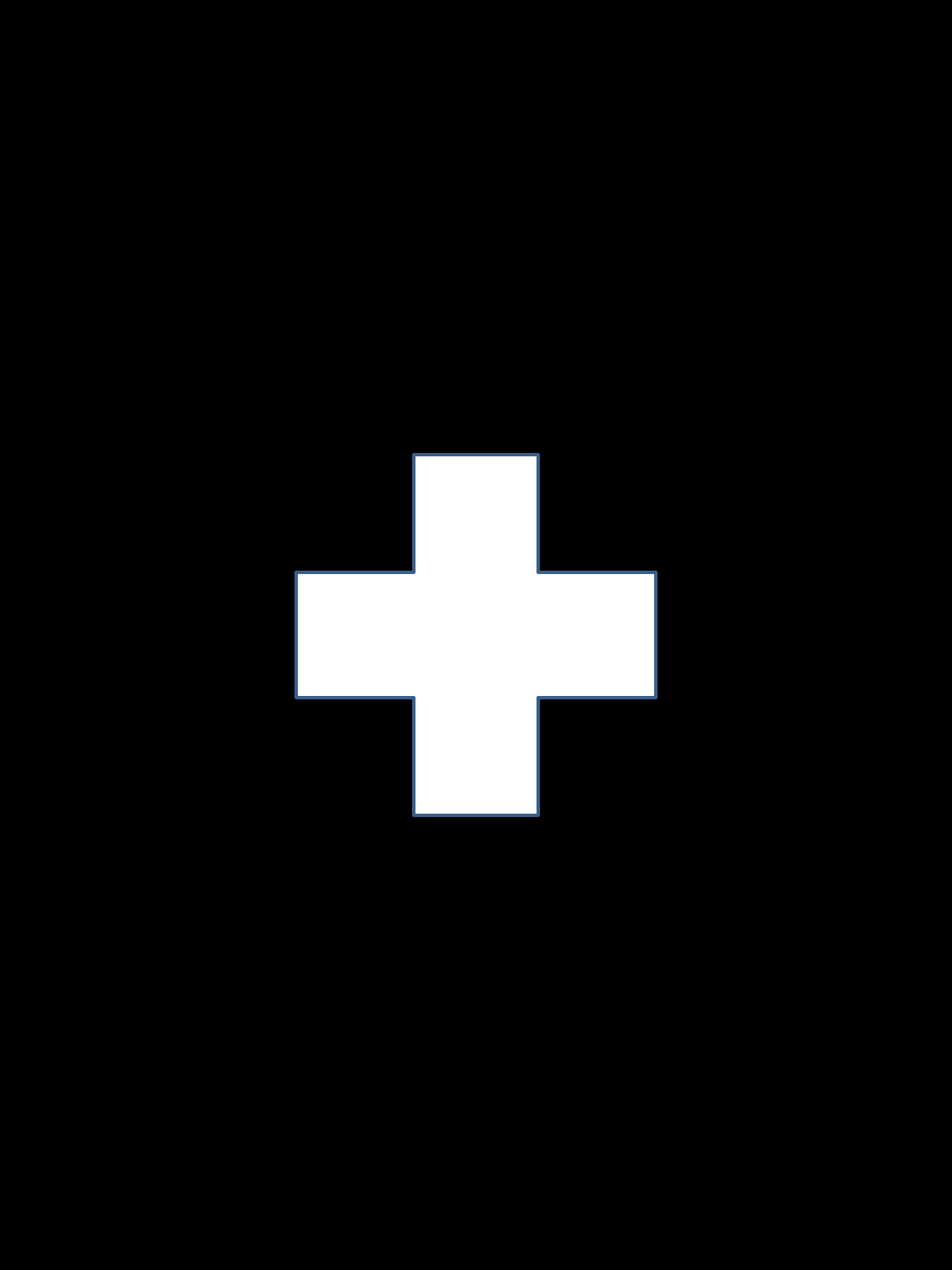

## Slide 27
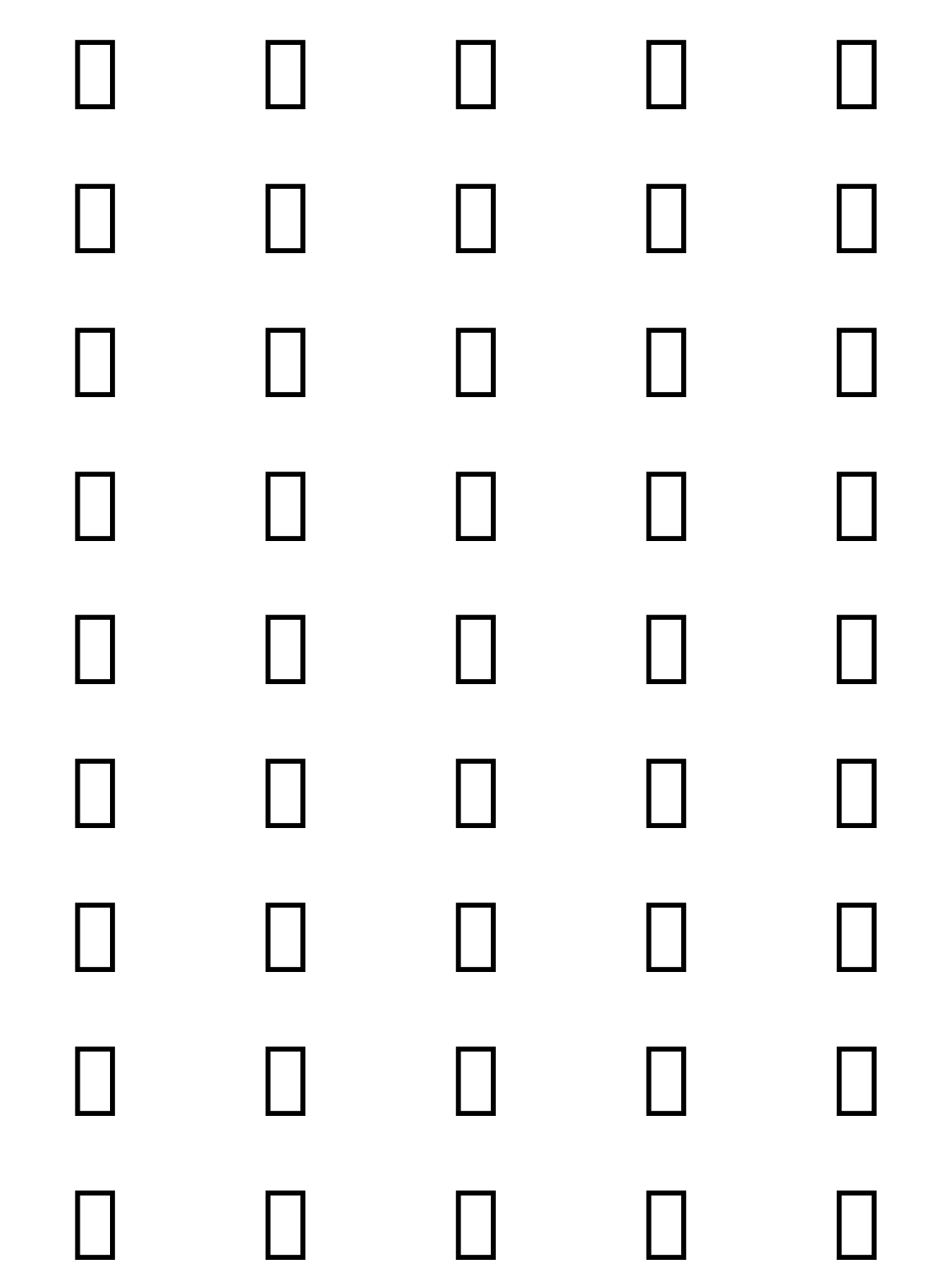

| ら | き | れ | ほ | や |
| --- | --- | --- | --- | --- |
| い | ん | せ | の | う |
| お | み | し | に | ひ |
| み | め | こ | と | へ |
| る | た | く | も | す |
| あ | え | ろ | そ | つ |
| か | を | ふ | わ | さ |
| け | ち | ぬ | て | な |
| ゆ | り | ま | ね | は |

## Slide 28
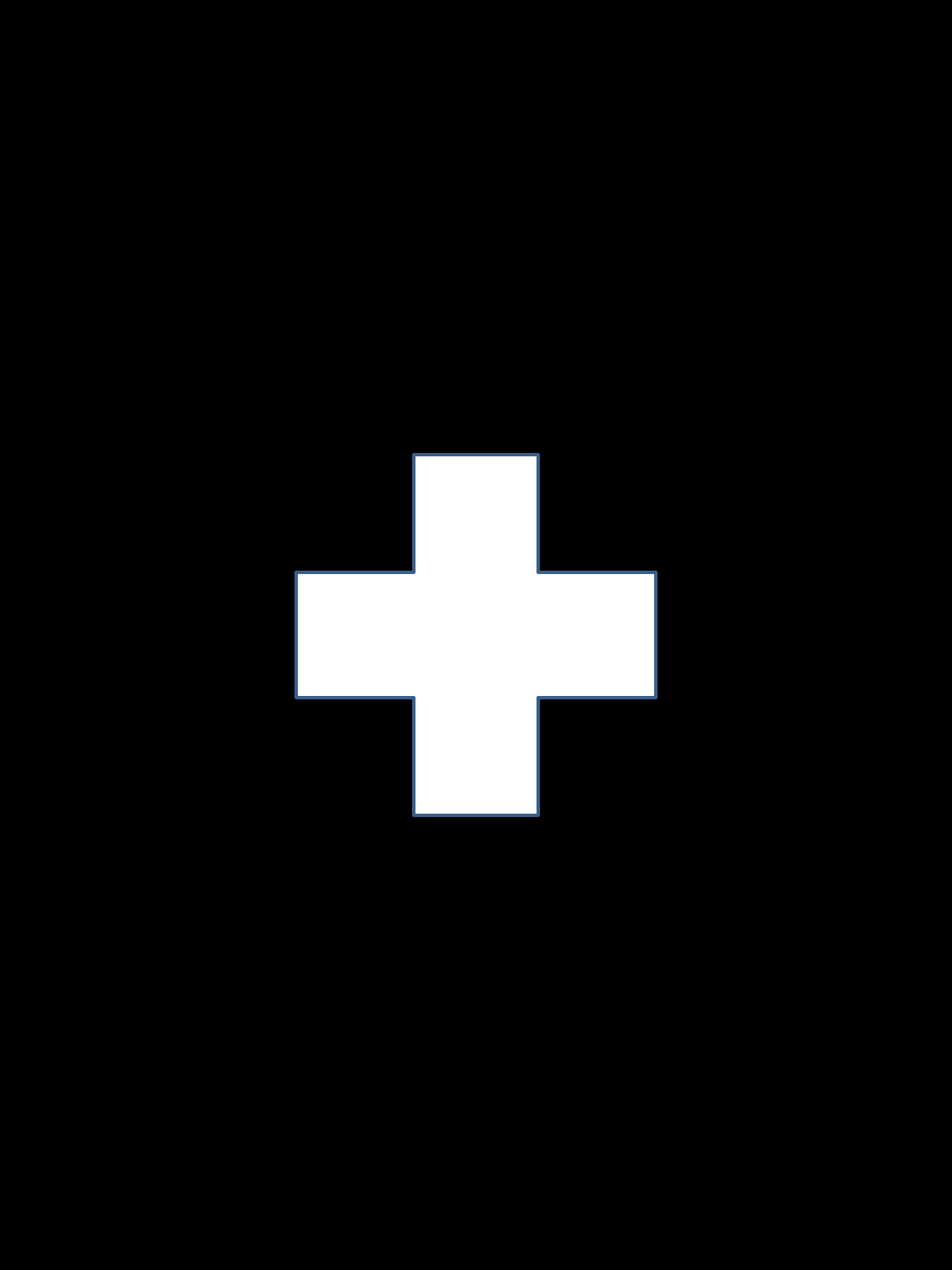

## Slide 29
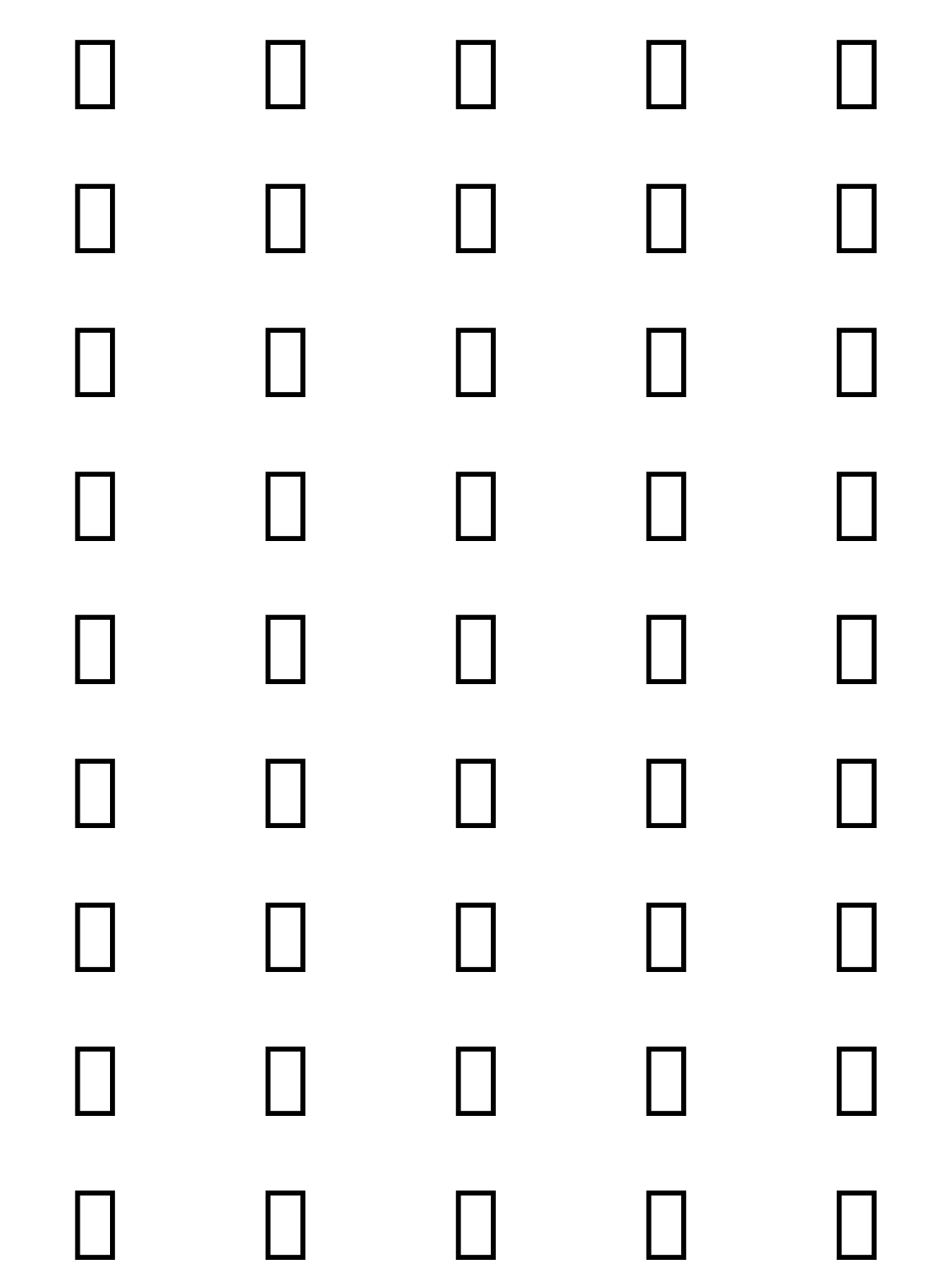

| の | せ | ん | い | や |
| --- | --- | --- | --- | --- |
| ほ | れ | き | ら | う |
| お | む | し | に | た |
| へ | こ | え | あ | す |
| も | く | み | ひ | あ |
| と | ろ | そ | つ | か |
| を | ふ | わ | さ | け |
| よ | は | ね | ま | り |
| ゆ | な | て | ぬ | ち |

## Slide 30
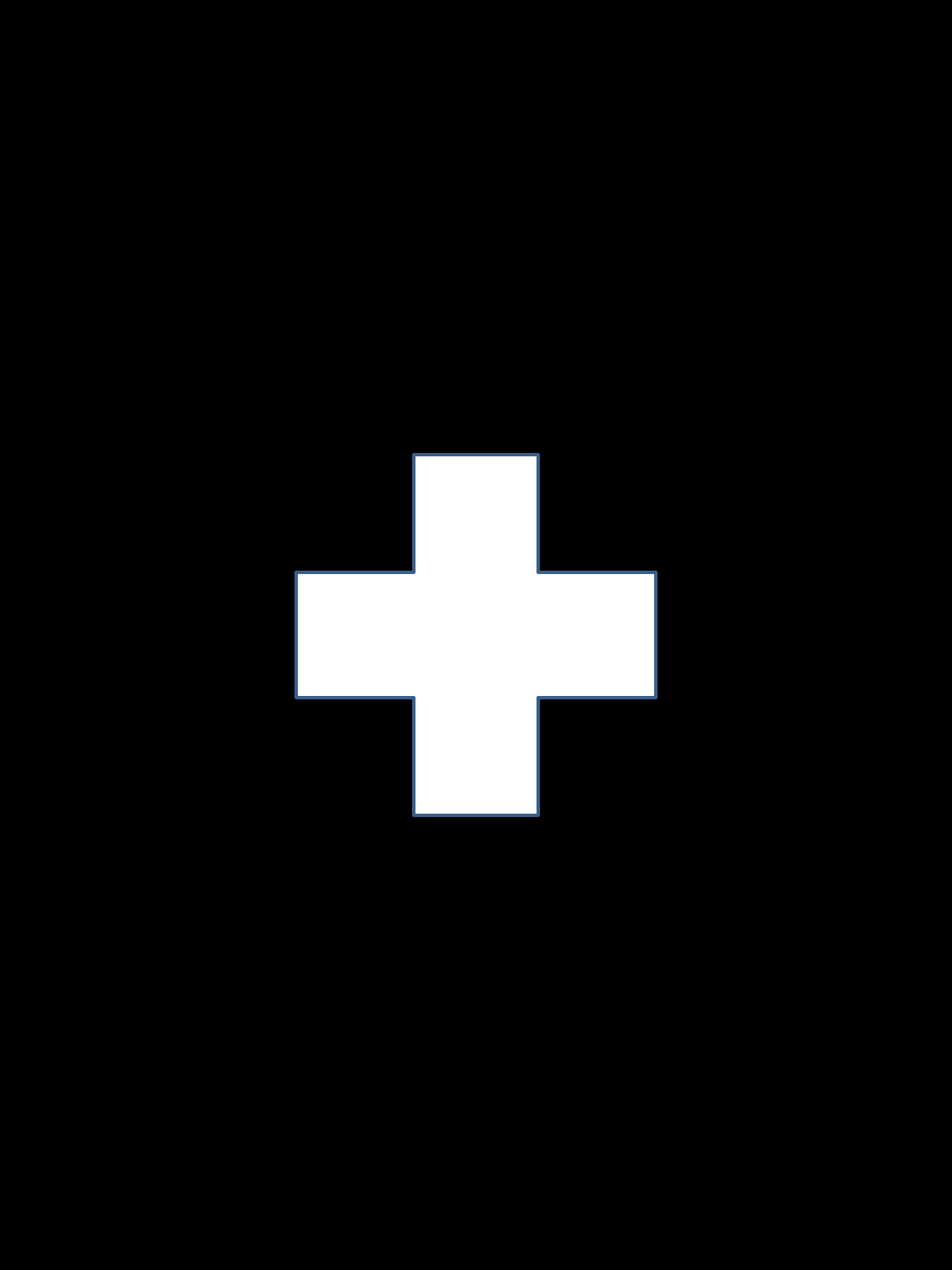

## Slide 31
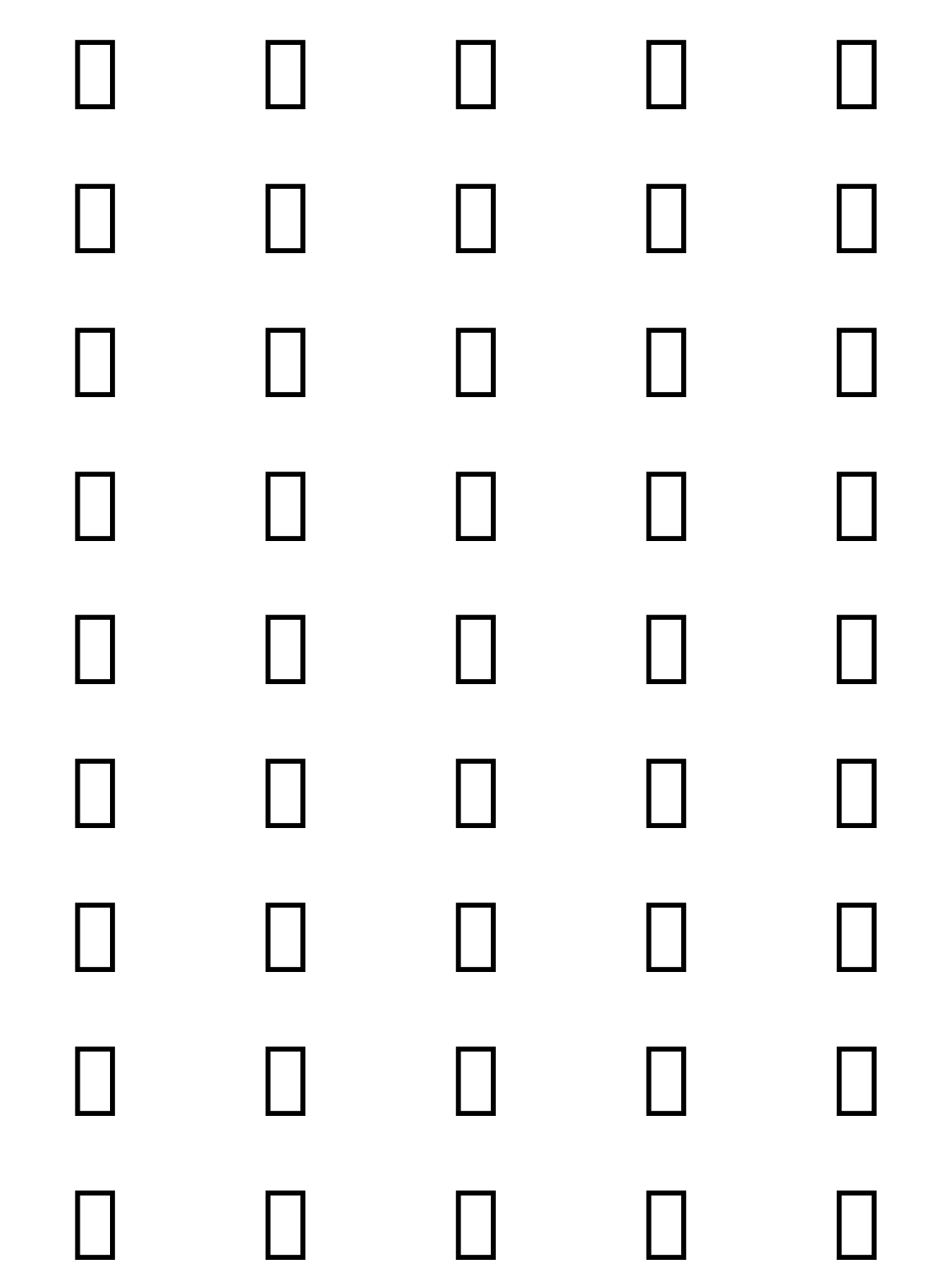

| む | お | こ | せ | そ |
| --- | --- | --- | --- | --- |
| た | い | あ | に | ふ |
| す | や | ほ | る | し |
| ろ | ら | か | め | れ |
| ち | て | ま | つ | ね |
| へ | わ | き | ぬ | ろ |
| く | け | さ | を | え |
| な | み | よ | ゆ | り |
| も | ひ | の | は | ん |

## Slide 32
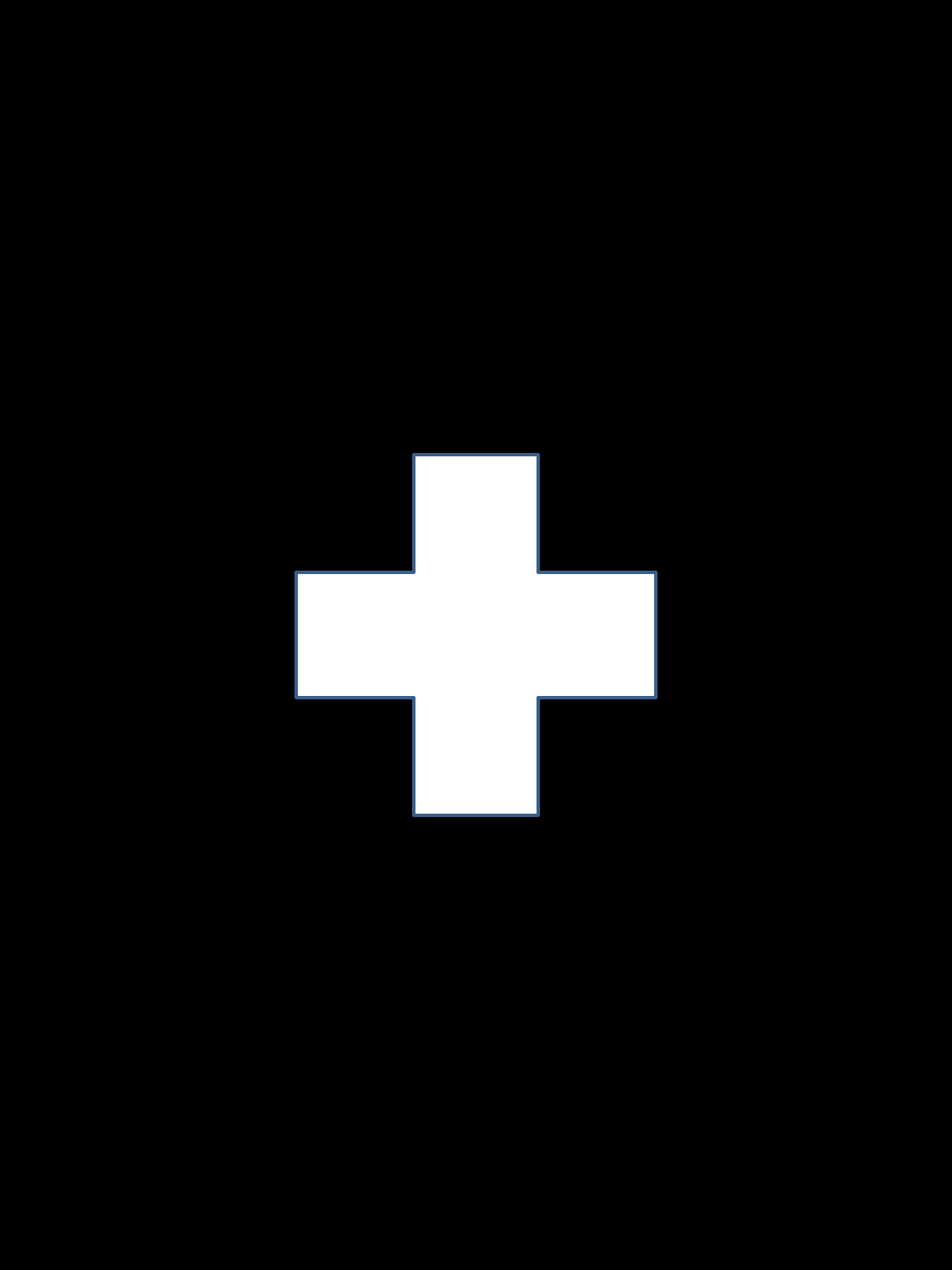

## Slide 33
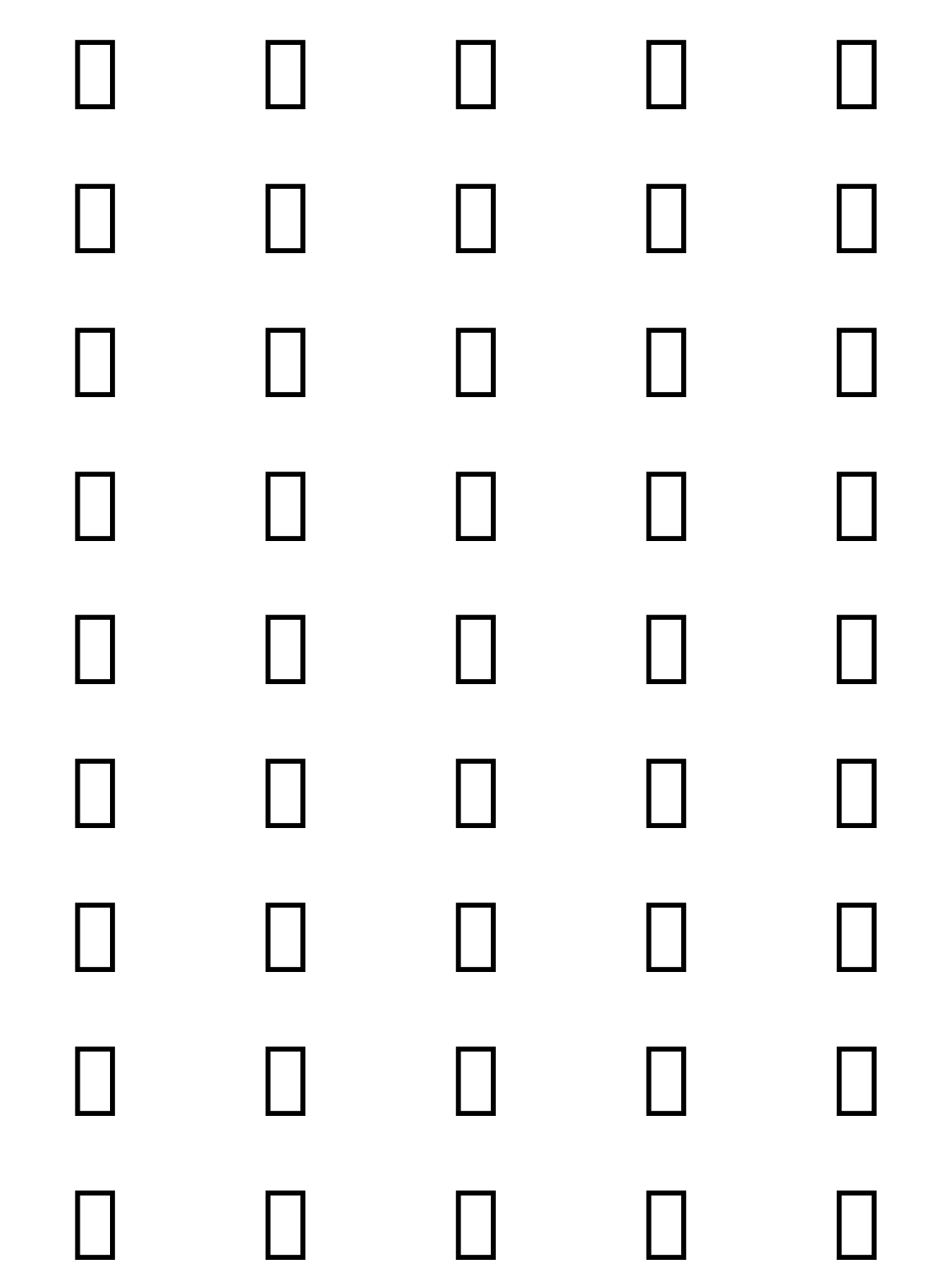

| そ | ふ | し | れ | ね |
| --- | --- | --- | --- | --- |
| う | え | り | ん | せ |
| に | る | め | つ | ぬ |
| を | ゆ | は | こ | あ |
| ほ | か | ま | き | さ |
| よ | の | お | い | や |
| ら | て | わ | け | み |
| ひ | う | む | た | す |
| ろ | ち | へ | く | な |

## Slide 34
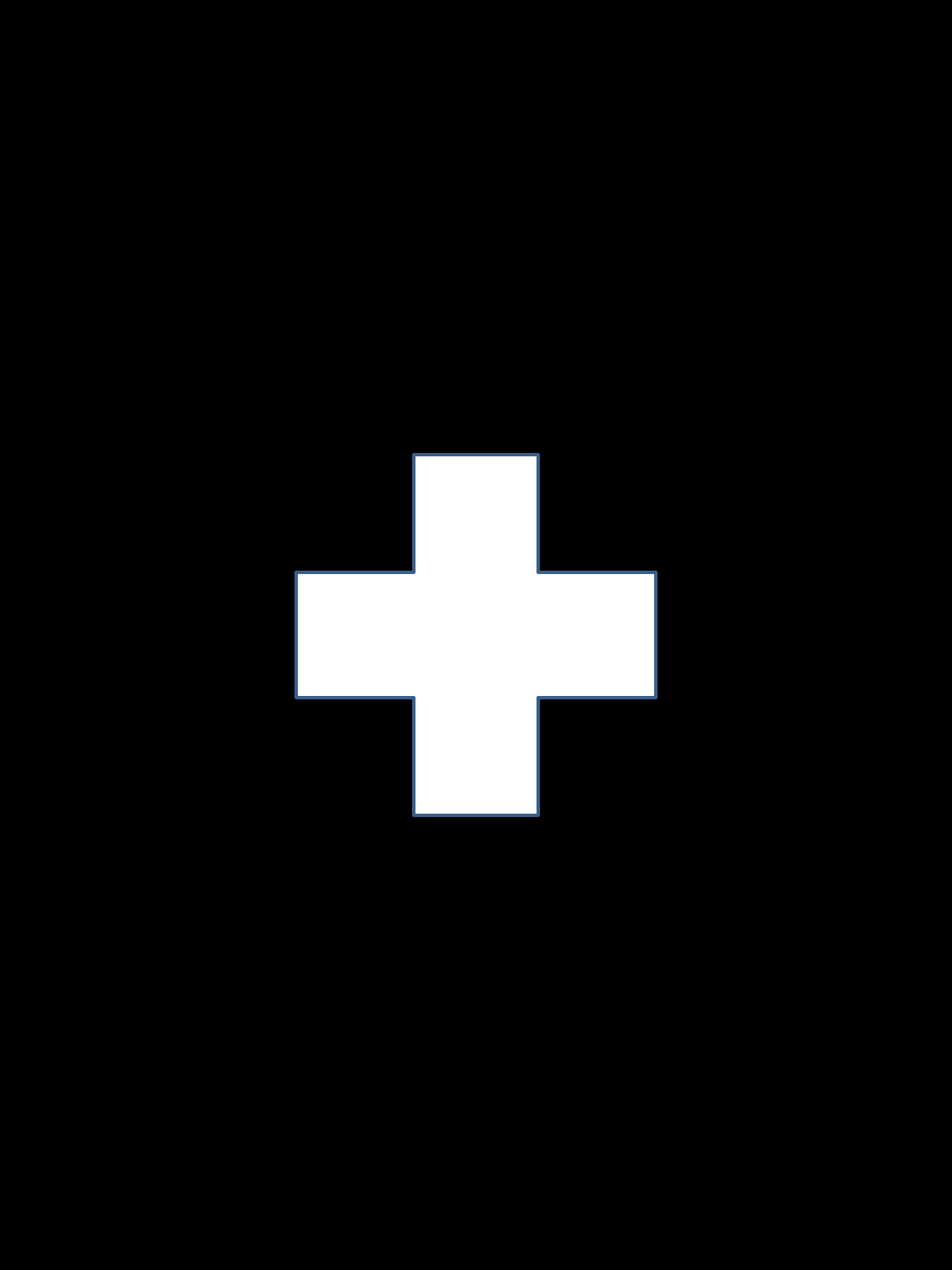

## Slide 35
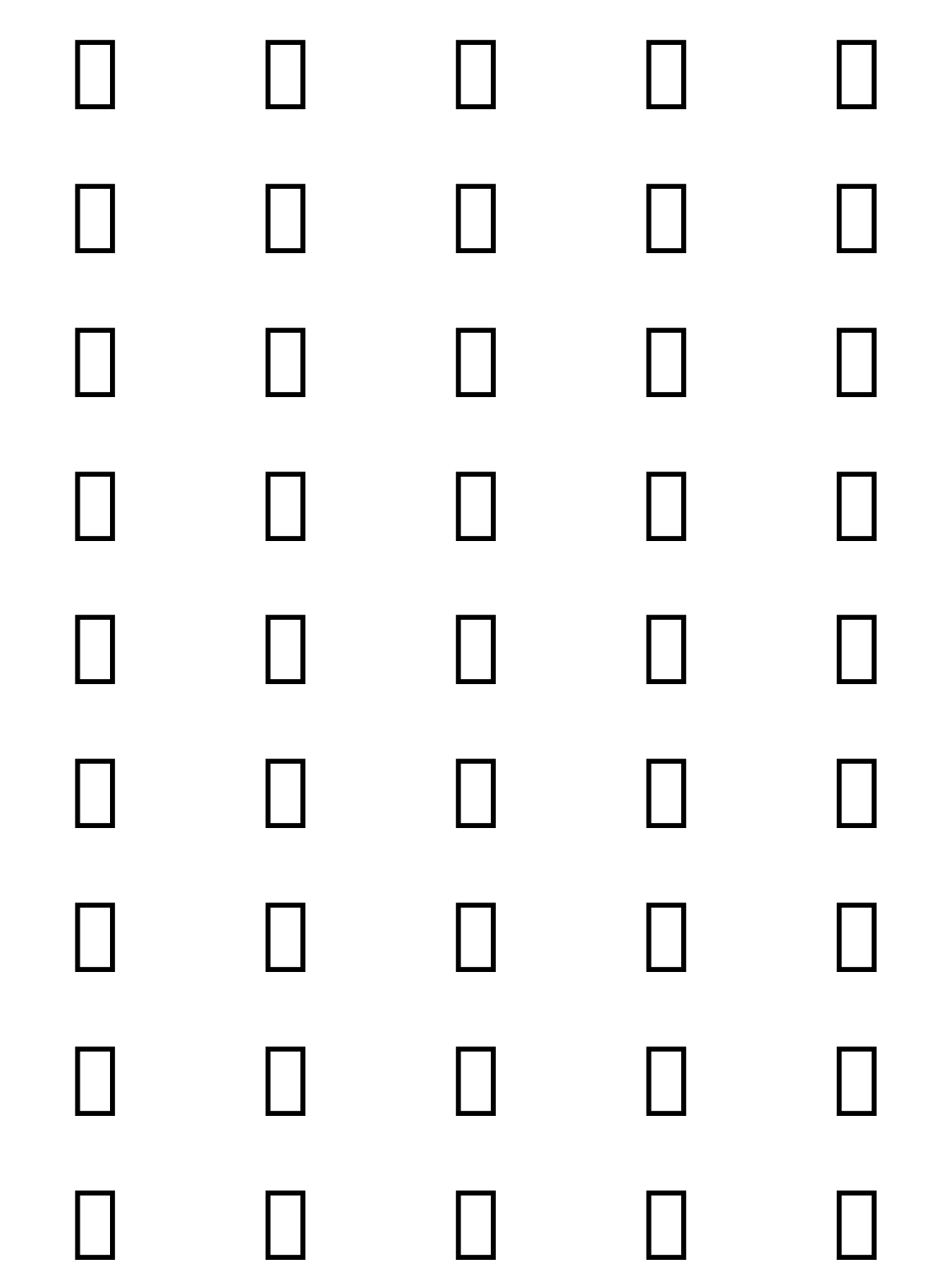

| ん | り | え | ろ | ね |
| --- | --- | --- | --- | --- |
| れ | し | ふ | そ | せ |
| に | る | め | つ | ぬ |
| を | ゆ | は | の | よ |
| さ | き | ま | か | ほ |
| あ | こ | お | い | や |
| ら | て | わ | け | み |
| ひ | も | な | く | へ |
| ち | ろ | す | た | む |

## Slide 36
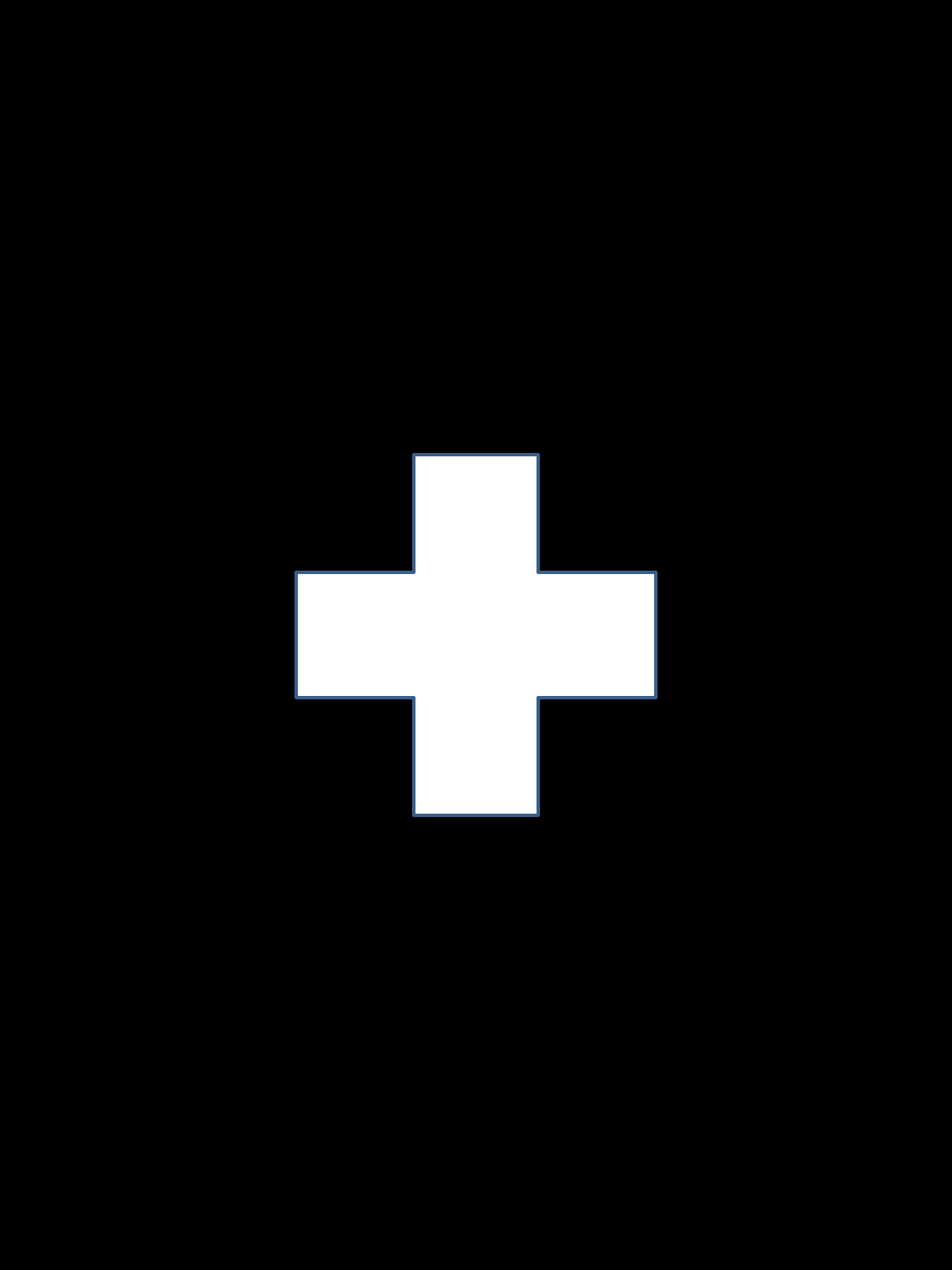

## Slide 37
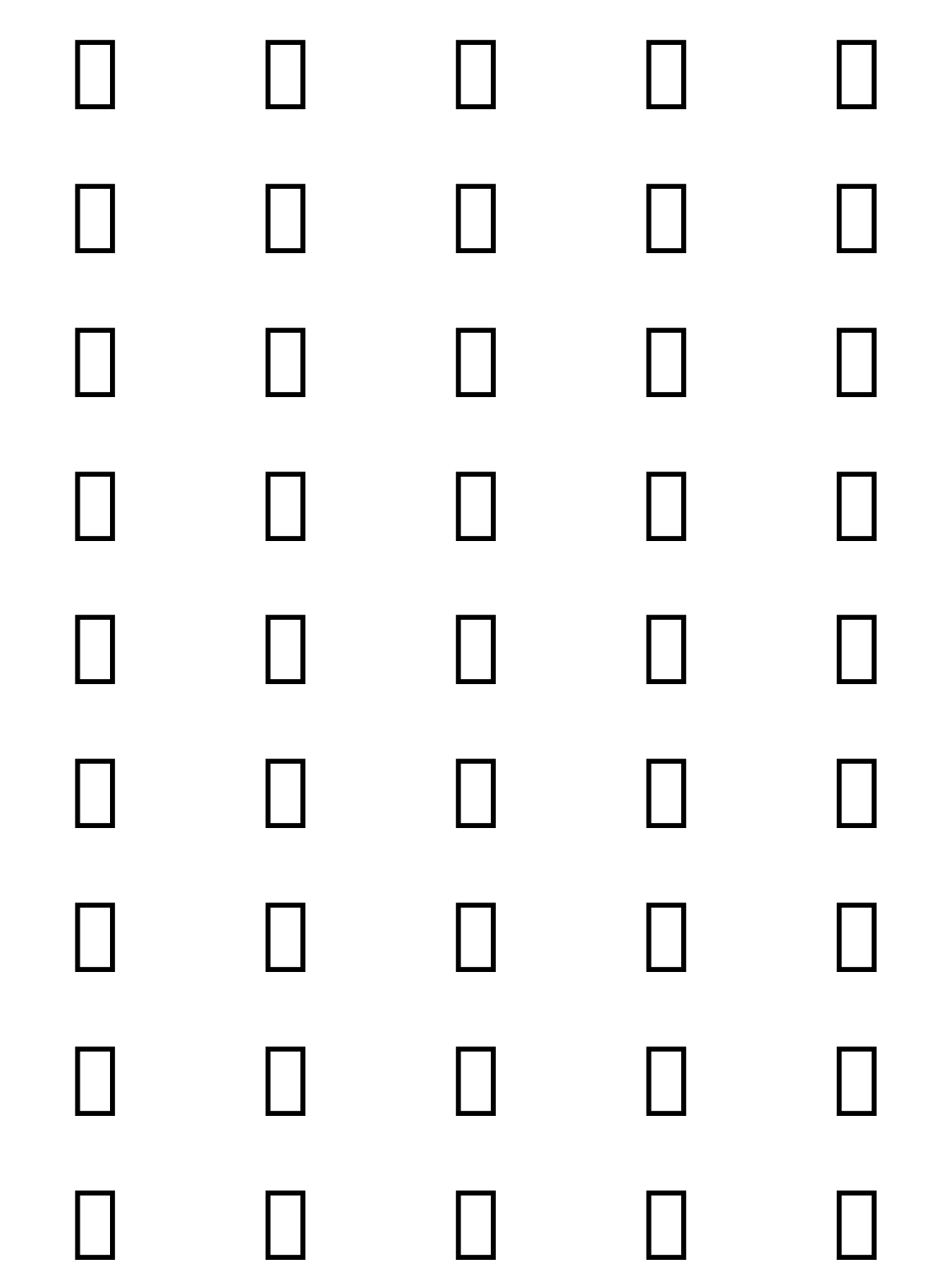

| ろ | ち | け | て | へ |
| --- | --- | --- | --- | --- |
| わ | ら | ま | つ | ね |
| き | く | な | ね | ほ |
| あ | こ | お | た | や |
| そ | も | の | ん | ひ |
| み | よ | は | さ | ゆ |
| を | り | え | ろ | か |
| ぬ | る | れ | し | す |
| い | に | む | う | せ |

## Slide 38
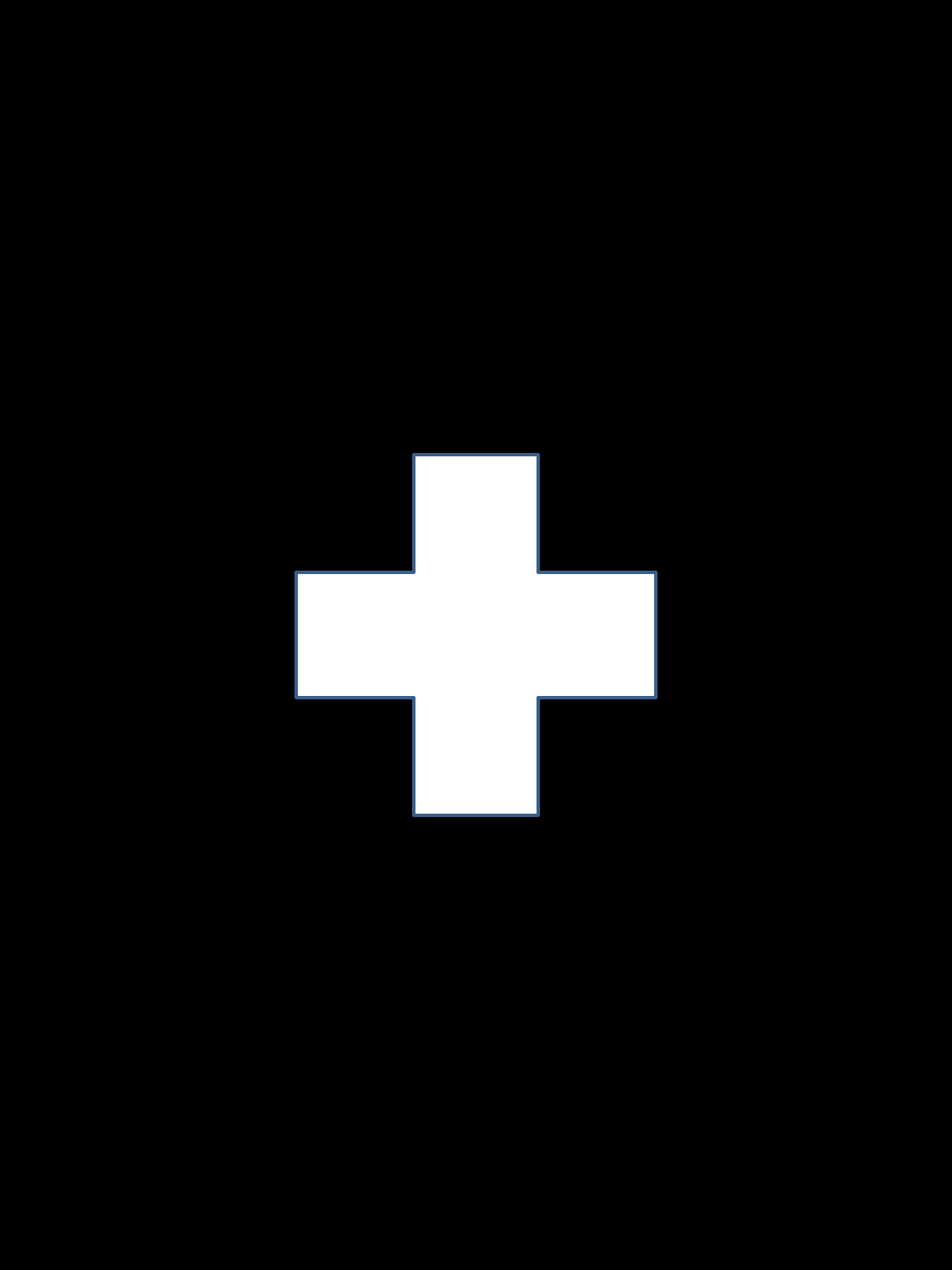

## Slide 39
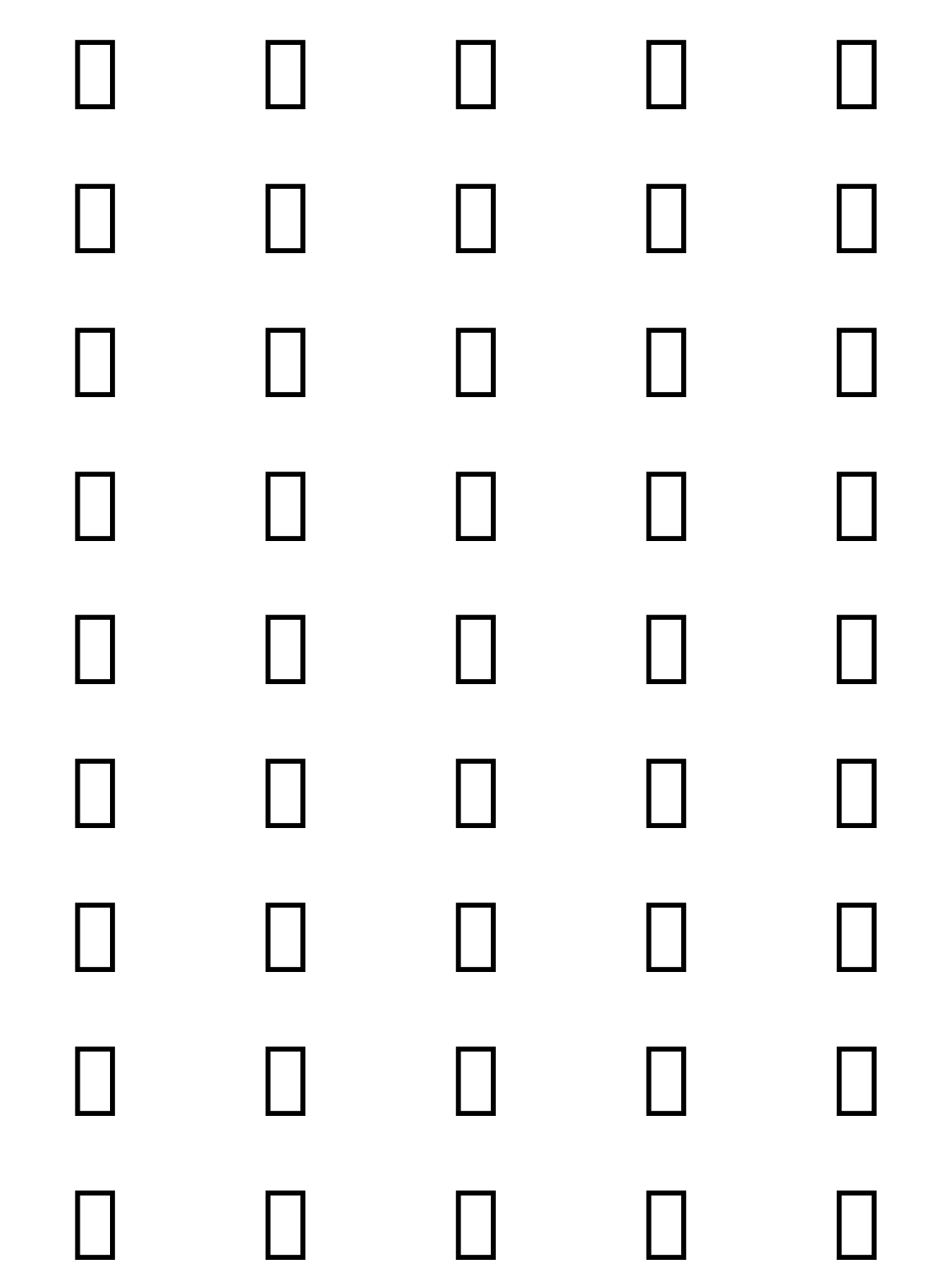

| し | そ | ふ | お | や |
| --- | --- | --- | --- | --- |
| ね | ま | せ | あ | り |
| つ | ぬ | ほ | を | よ |
| な | ゆ | き | く | け |
| の | れ | は | す | た |
| こ | ち | ら | み | わ |
| へ | め | て | ろ | い |
| さ | に | ひ | う | も |
| ん | る | か | え | ろ |

## Slide 40
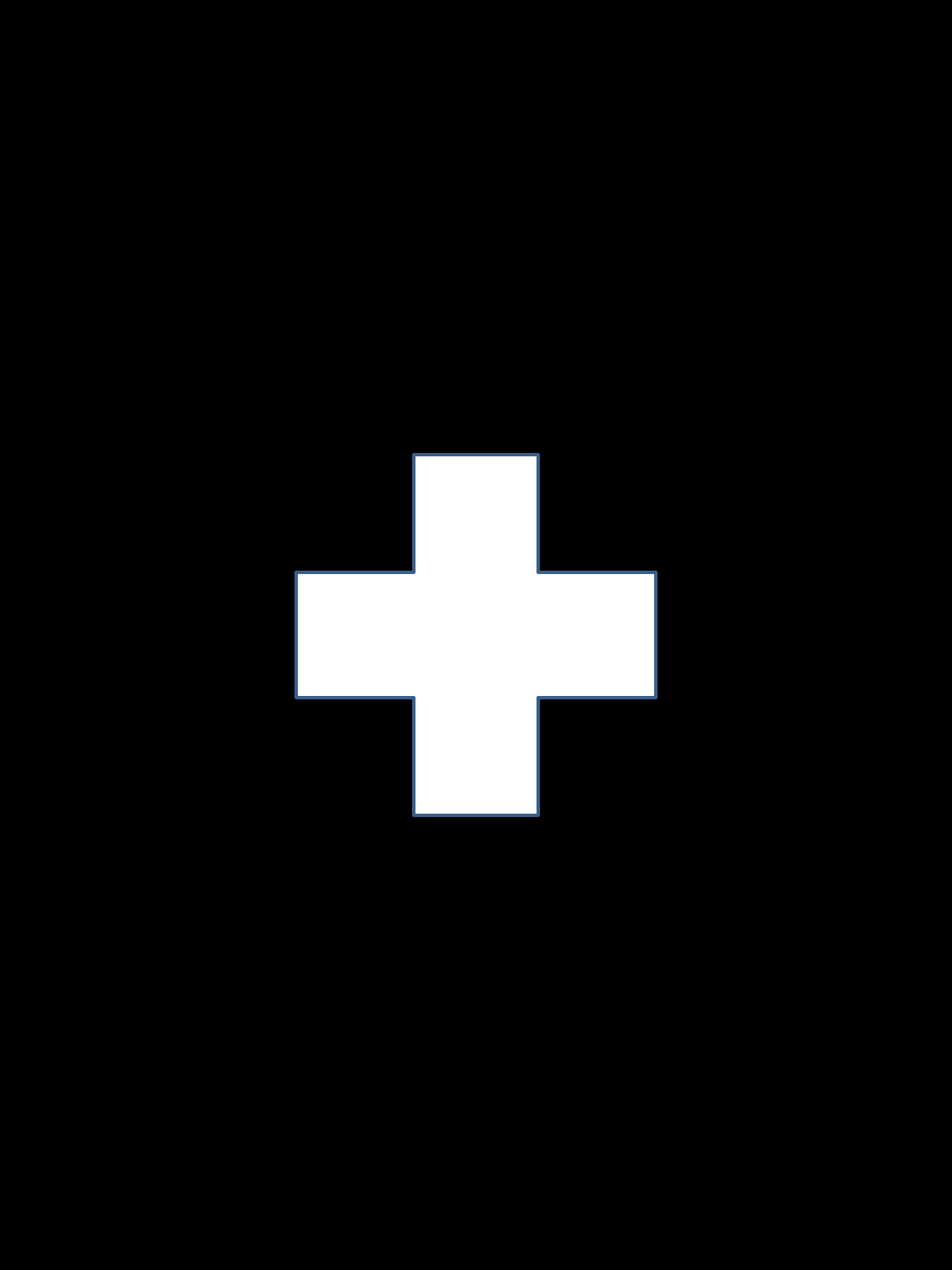

## Slide 41
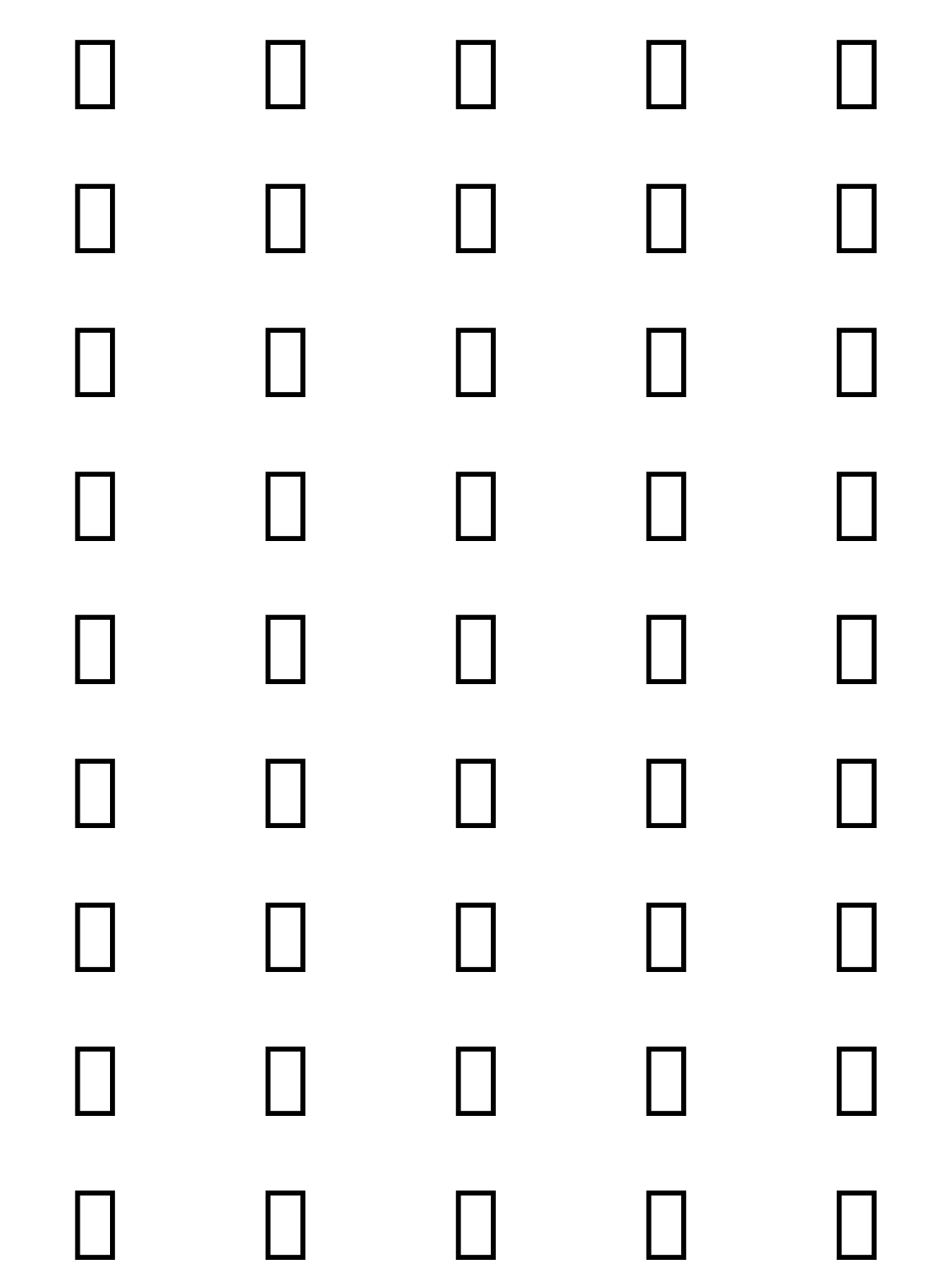

| に | そ | え | ね | う |
| --- | --- | --- | --- | --- |
| か | ひ | て | し | い |
| き | ち | ろ | む | や |
| も | け | あ | せ | く |
| ろ | わ | ゆ | ふ | な |
| さ | め | り | ら | み |
| ん | ま | つ | ぬ | へ |
| る | ほ | た | す | こ |
| は | の | れ | お | よ |

## Slide 42
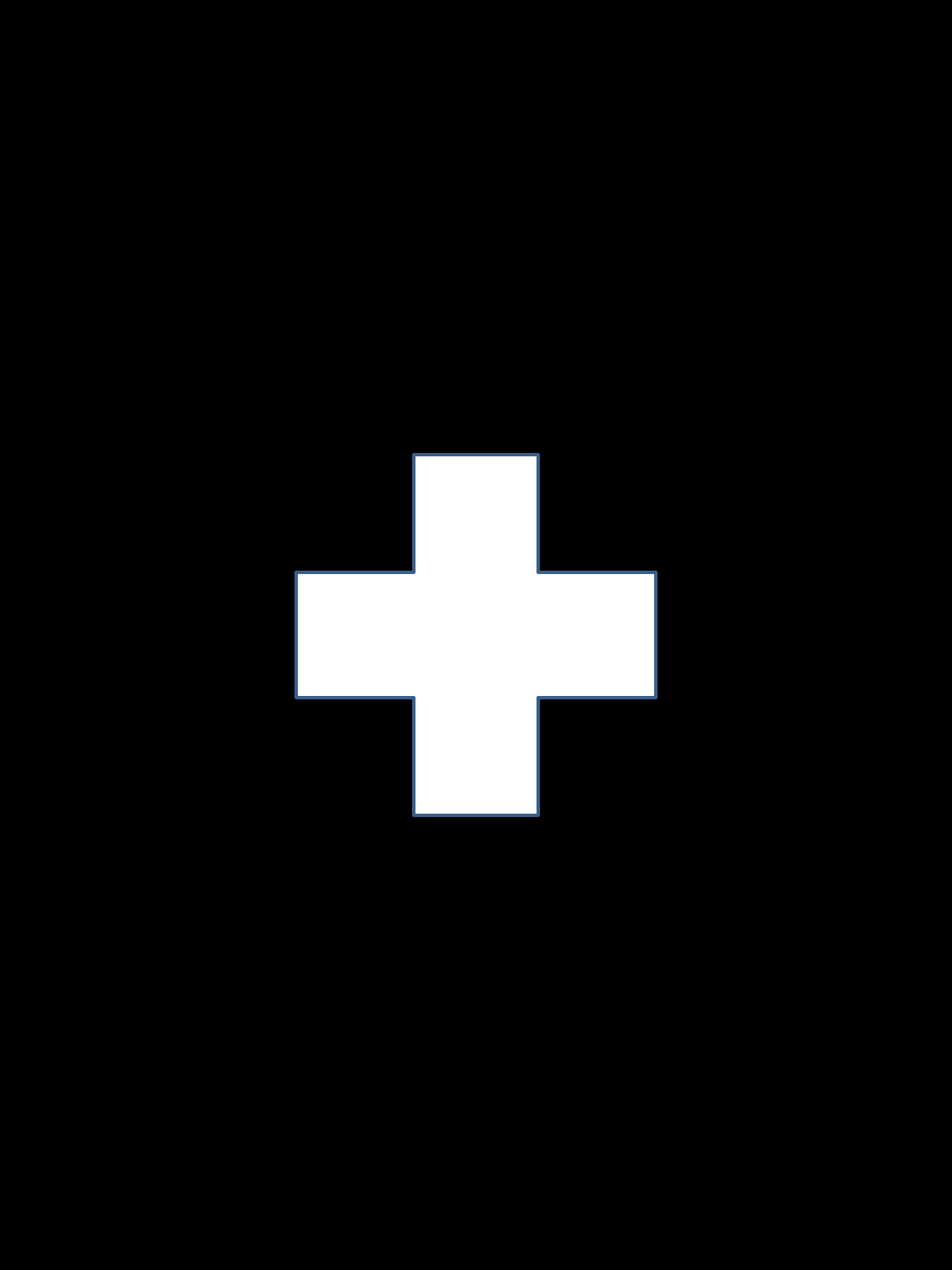

## Slide 43
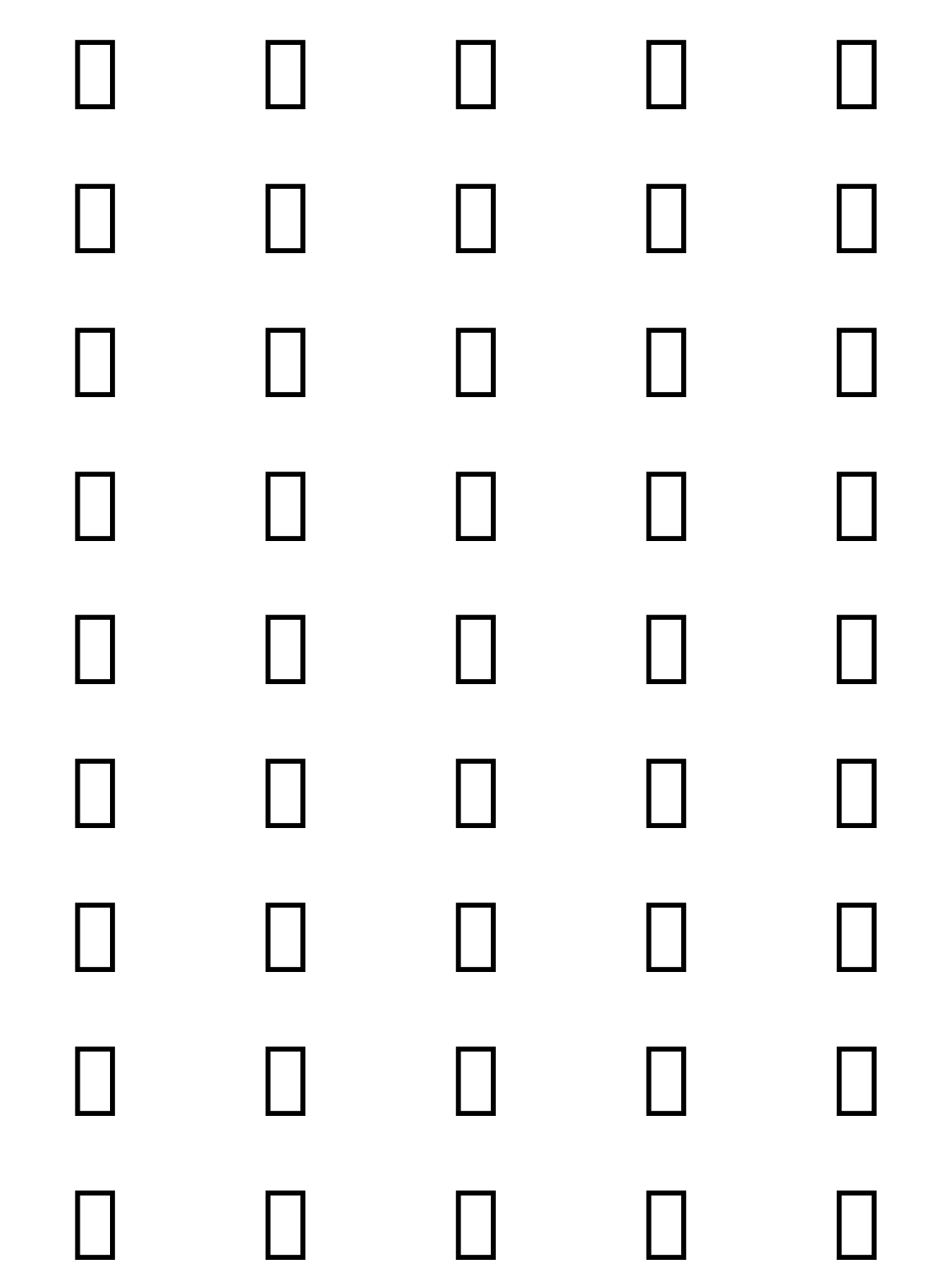

| や | て | ん | え | さ |
| --- | --- | --- | --- | --- |
| の | い | つ | か | む |
| る | め | う | ひ | せ |
| わ | ふ | ち | れ | お |
| あ | ほ | け | ま | せ |
| を | ろ | り | す | へ |
| ね | に | ら | ろ | み |
| よ | く | は | も | ゆ |
| き | こ | な | し | ぬ |

## Slide 44
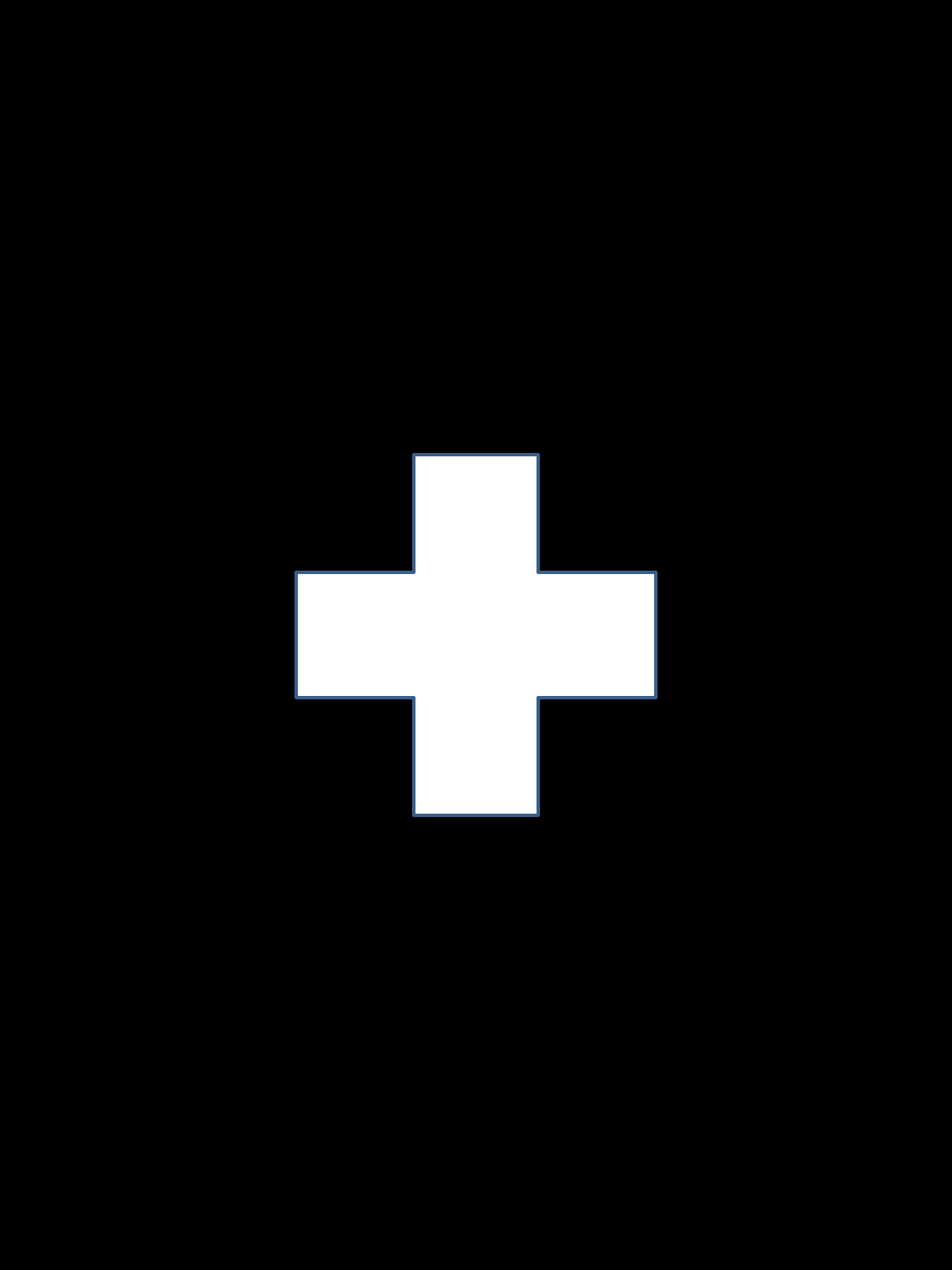

## Slide 45
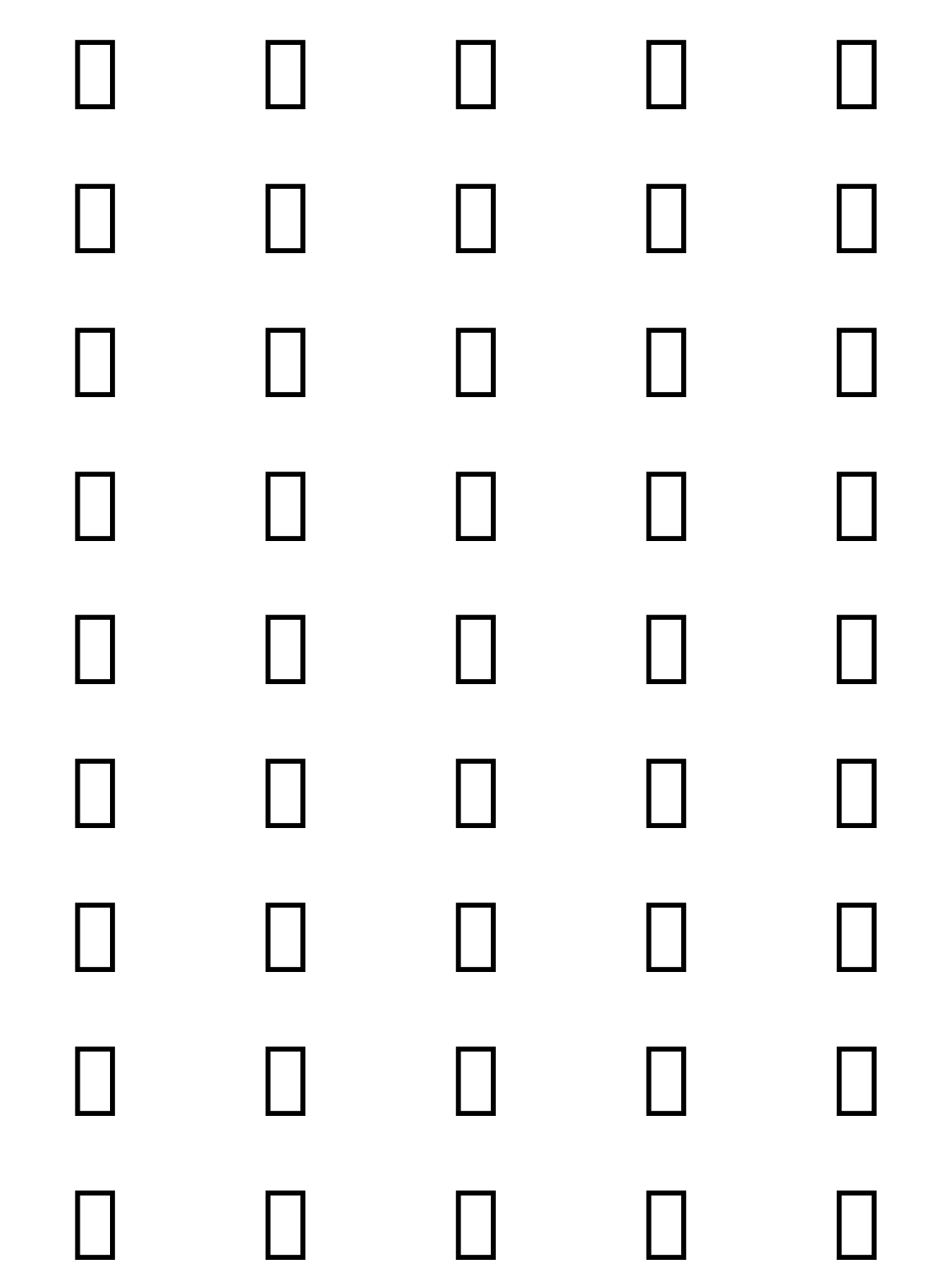

| そ | き | に | ひ | く |
| --- | --- | --- | --- | --- |
| て | ね | り | う | し |
| と | え | お | か | ん |
| の | け | い | わ | や |
| へ | も | ろ | さ | れ |
| た | ら | な | せ | す |
| よ | り | む | ま | み |
| は | ほ | ゆ | を | る |
| め | ふ | あ | ぬ | こ |

## Slide 46
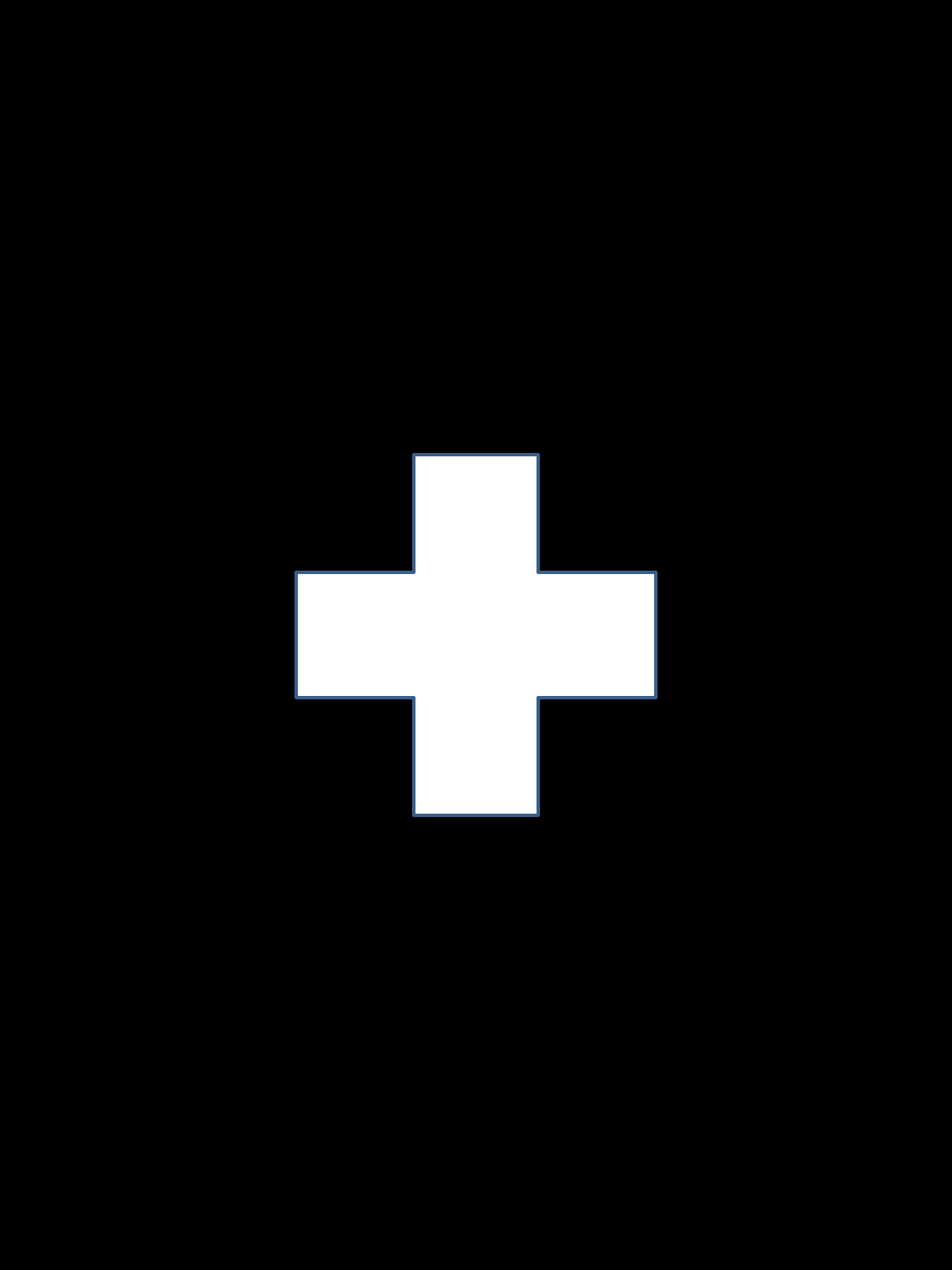

## Slide 47
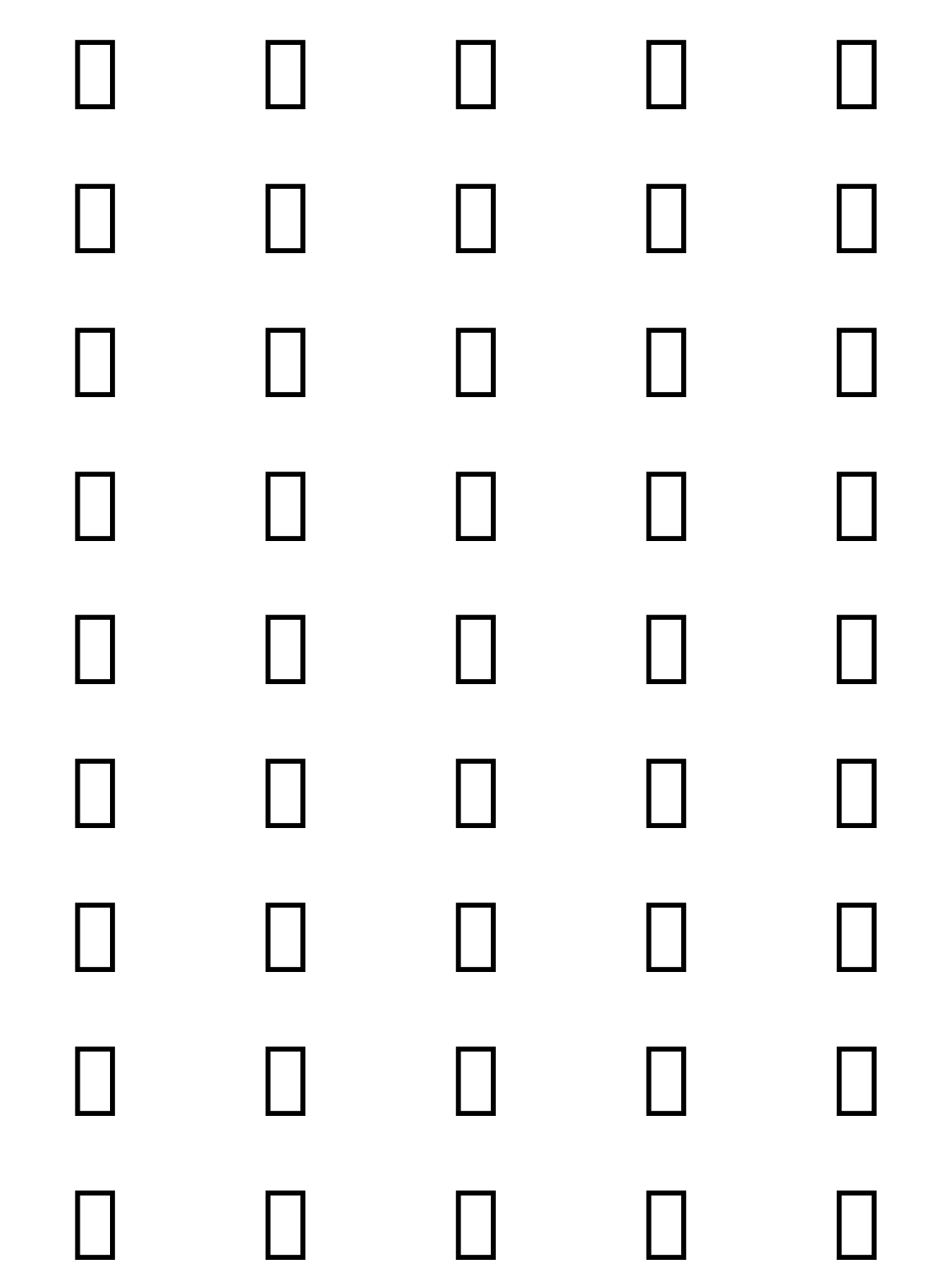

| る | よ | も | え | そ |
| --- | --- | --- | --- | --- |
| め | り | ろ | お | き |
| ふ | む | さ | か | に |
| あ | ま | れ | ん | ひ |
| ぬ | み | た | の | て |
| こ | は | ら | け | ね |
| つ | ほ | な | い | ち |
| ゆ | を | せ | わ | う |
| へ | す | と | や | し |

## Slide 48
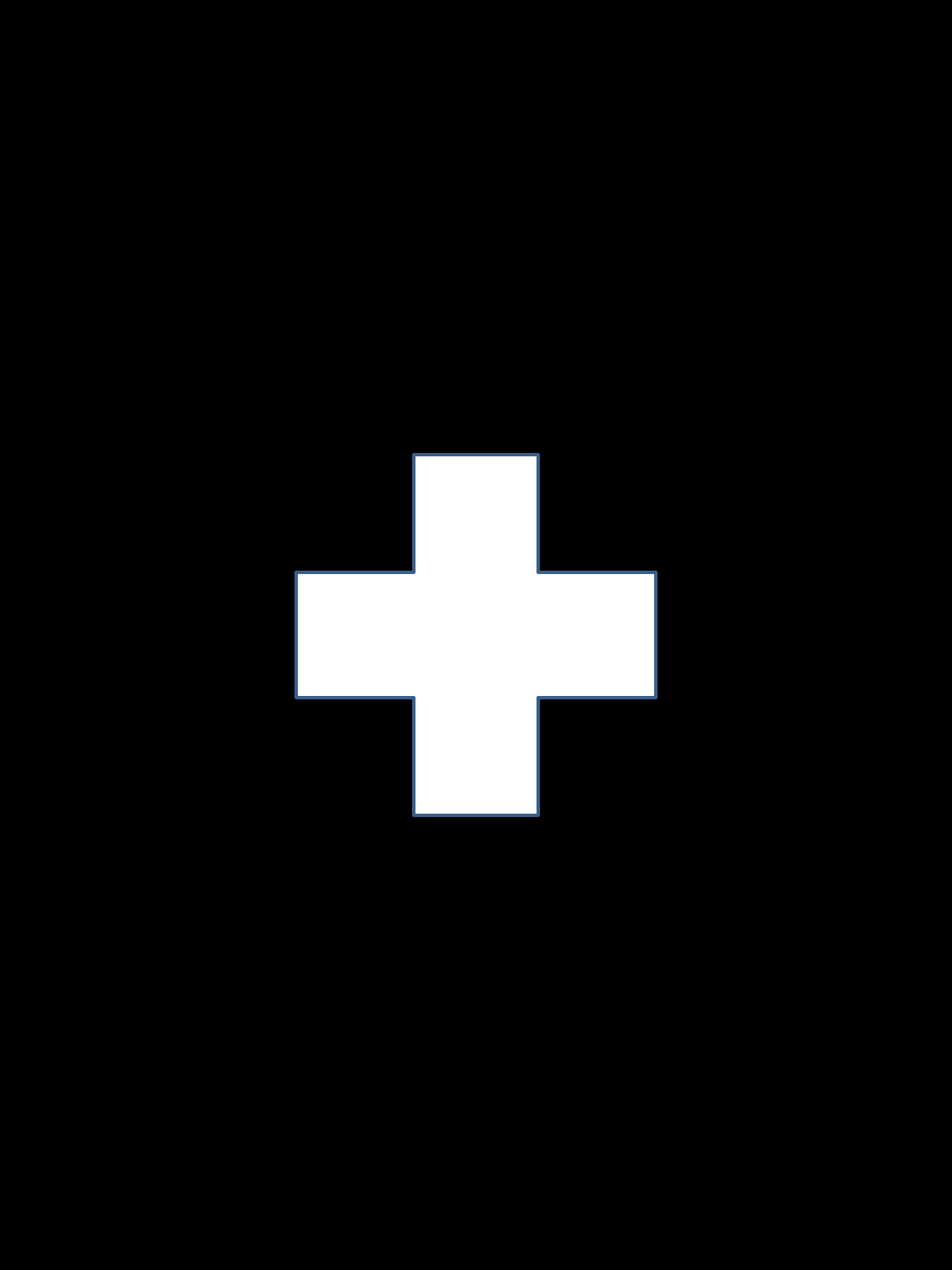

## Slide 49
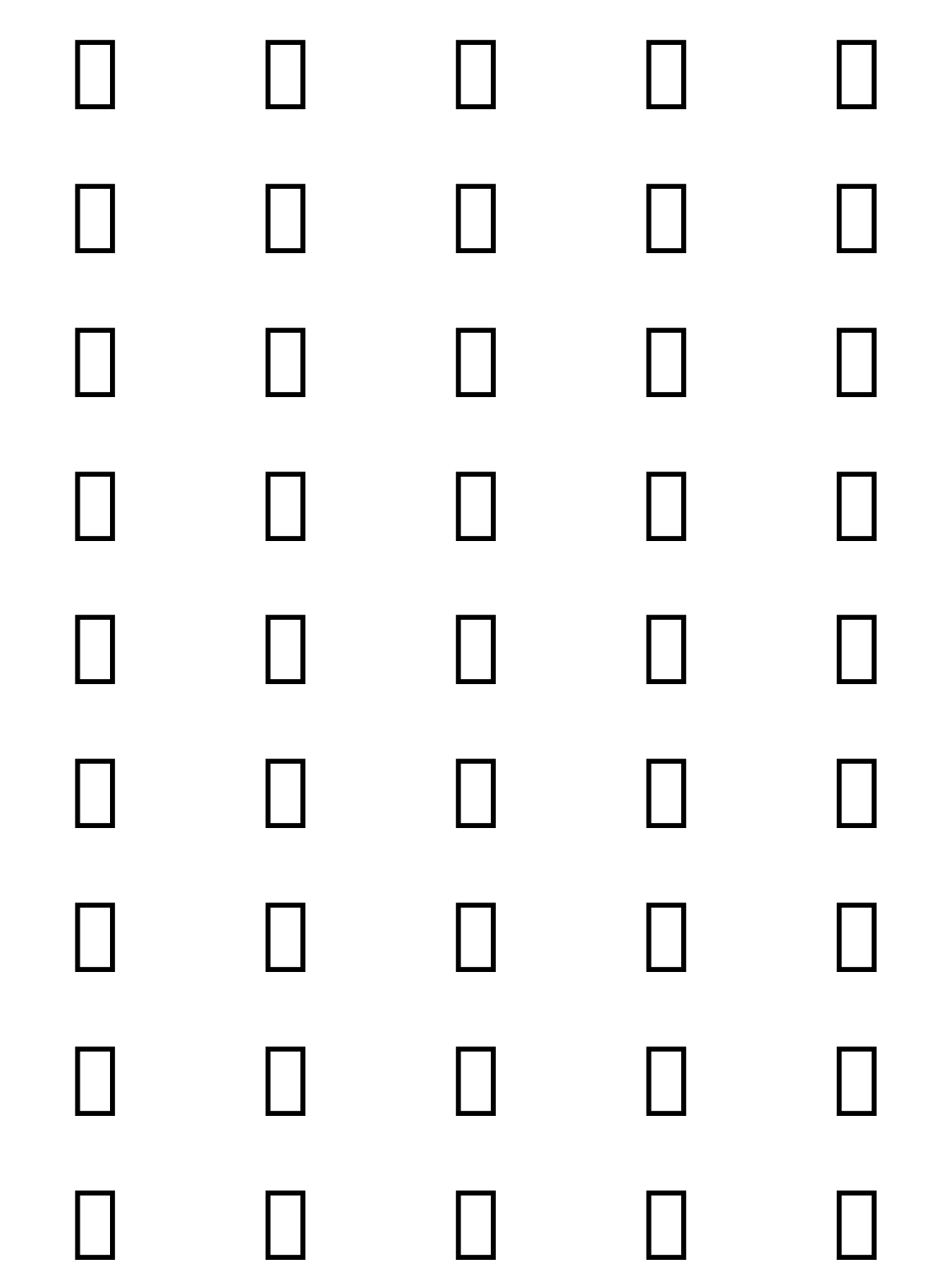

| そ | え | も | よ | く |
| --- | --- | --- | --- | --- |
| き | お | ろ | り | め |
| に | か | さ | む | ふ |
| ひ | ん | れ | ま | あ |
| て | の | た | み | ぬ |
| ね | け | た | は | こ |
| ち | い | な | ほ | つ |
| う | わ | せ | を | ゆ |
| し | や | と | す | へ |

## Slide 50
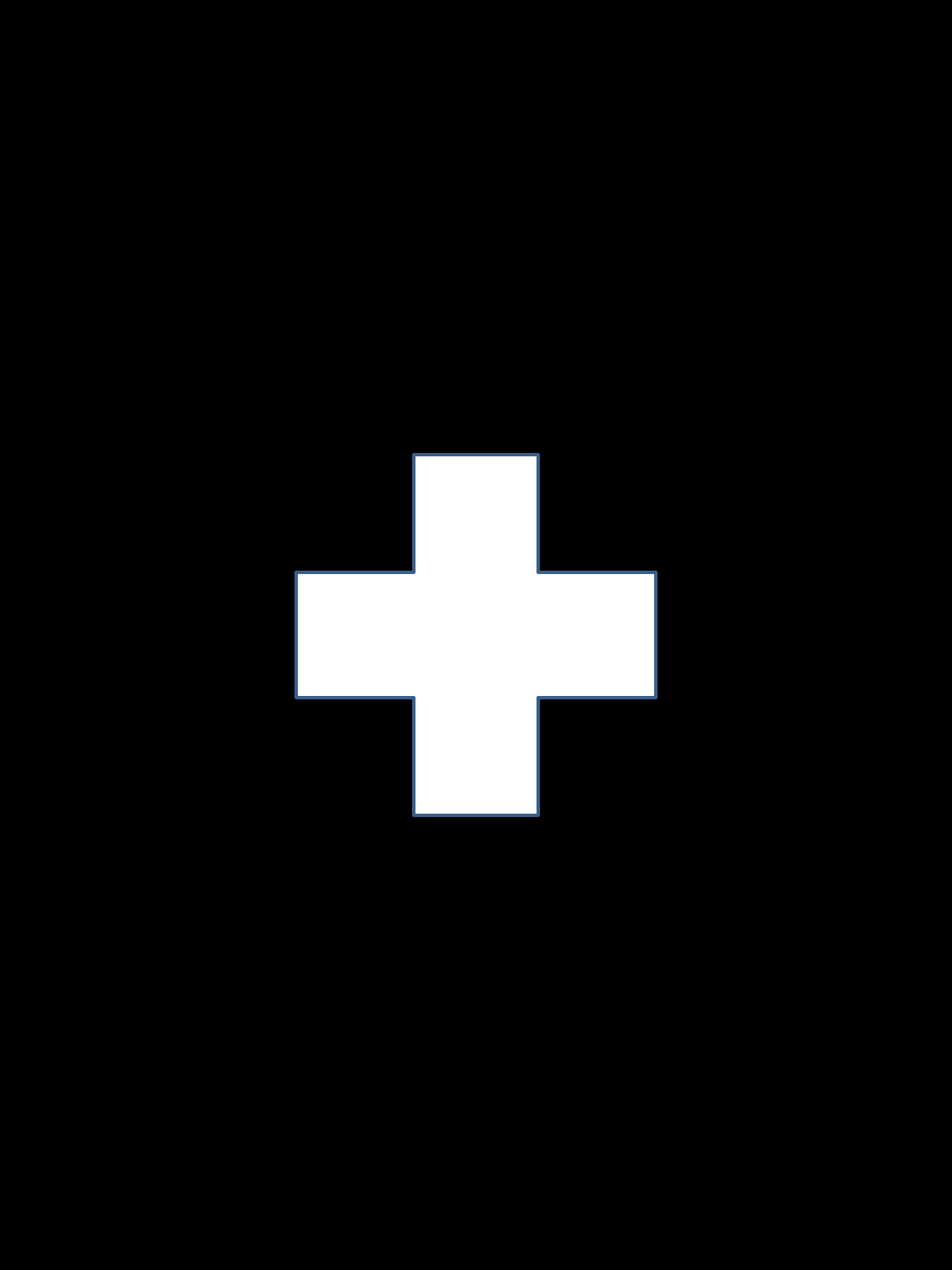

## Slide 51
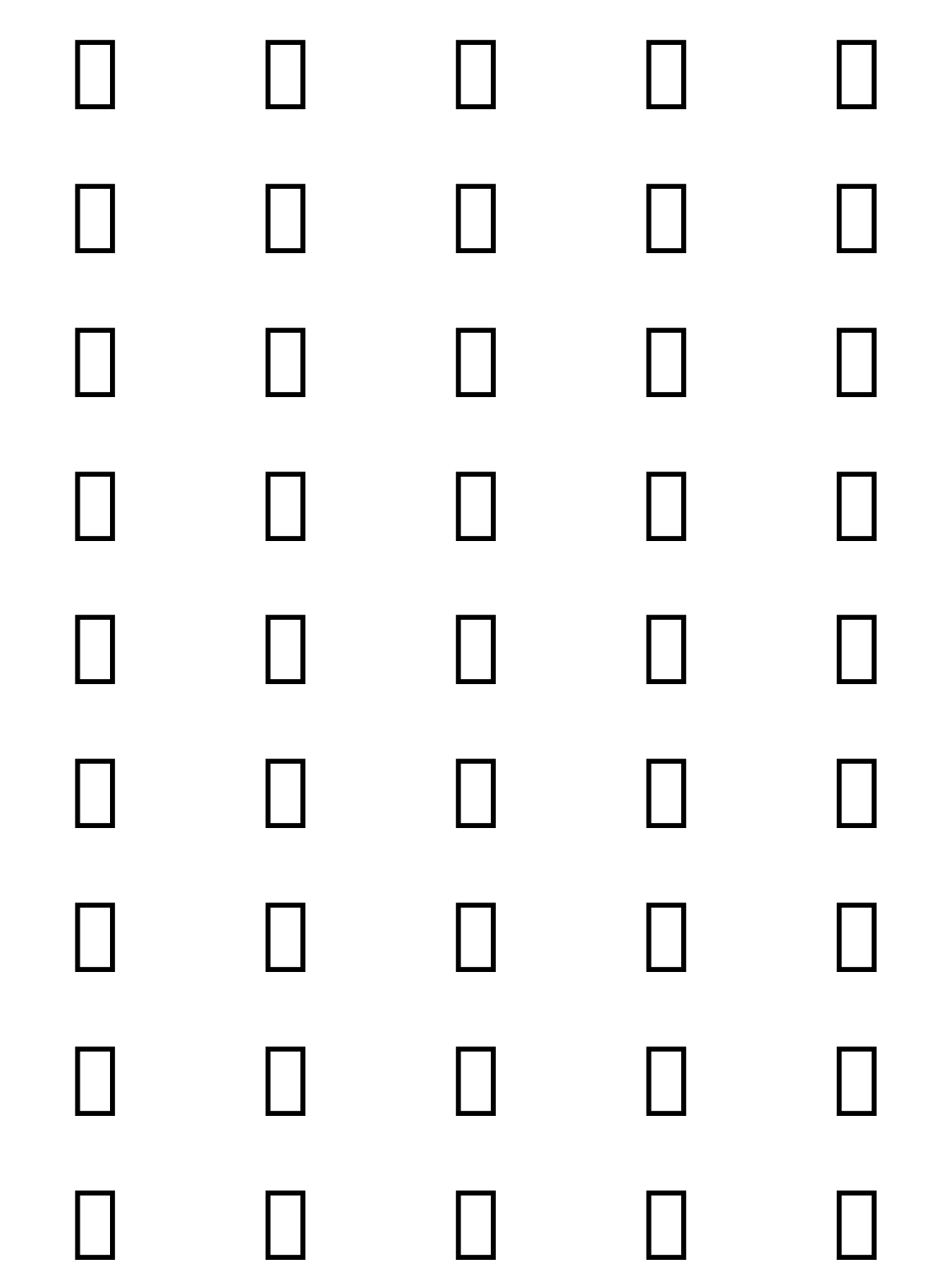

| み | ね | に | ひ | く |
| --- | --- | --- | --- | --- |
| ち | や | と | し | う |
| て | け | よ | り | む |
| ま | い | わ | な | の |
| ん | こ | つ | は | ほ |
| を | す | せ | か | お |
| る | め | ふ | あ | ぬ |
| ゆ | へ | え | た | ら |
| も | ろ | そ | き | さ |

## Slide 52
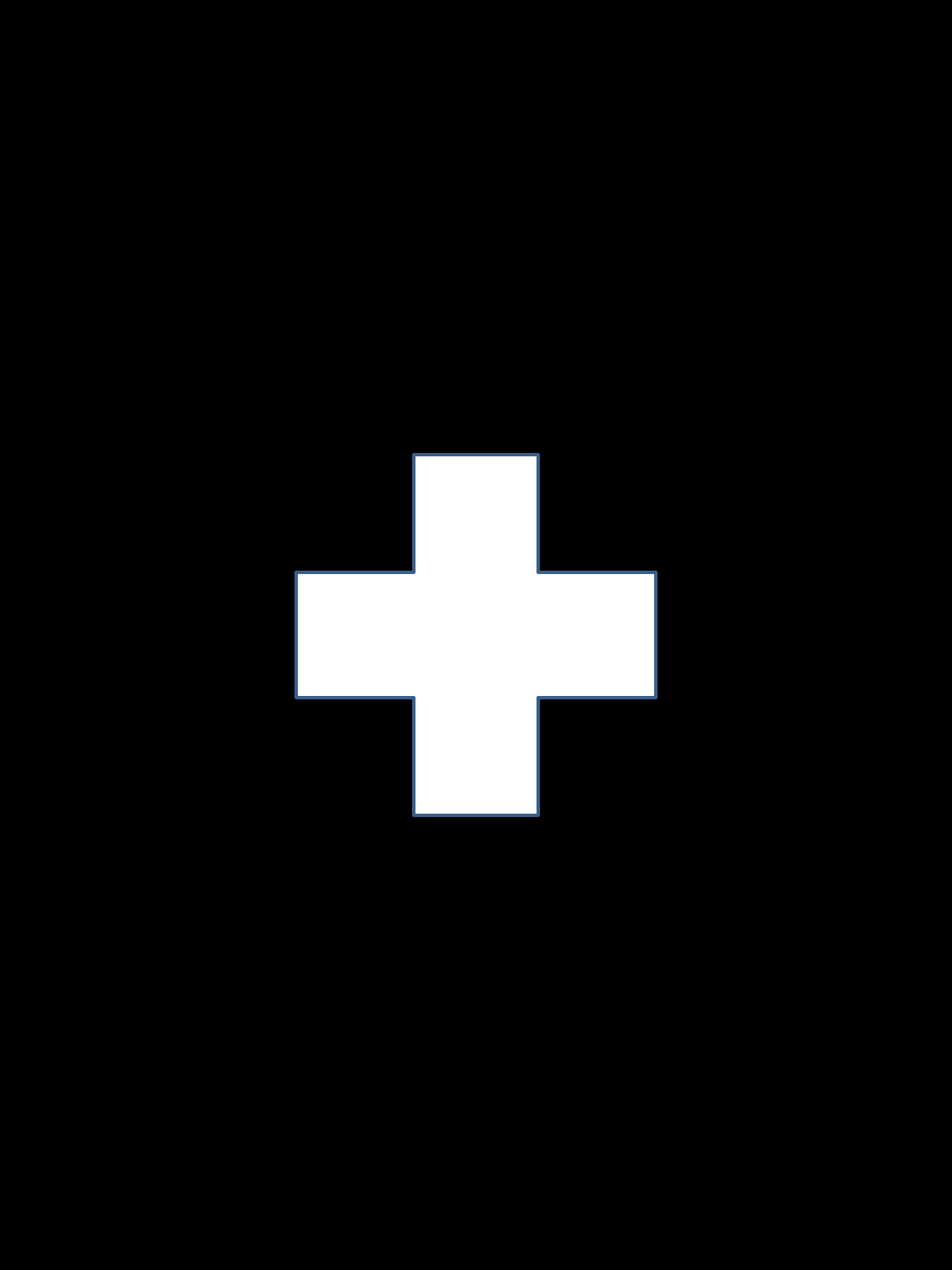

## Slide 53
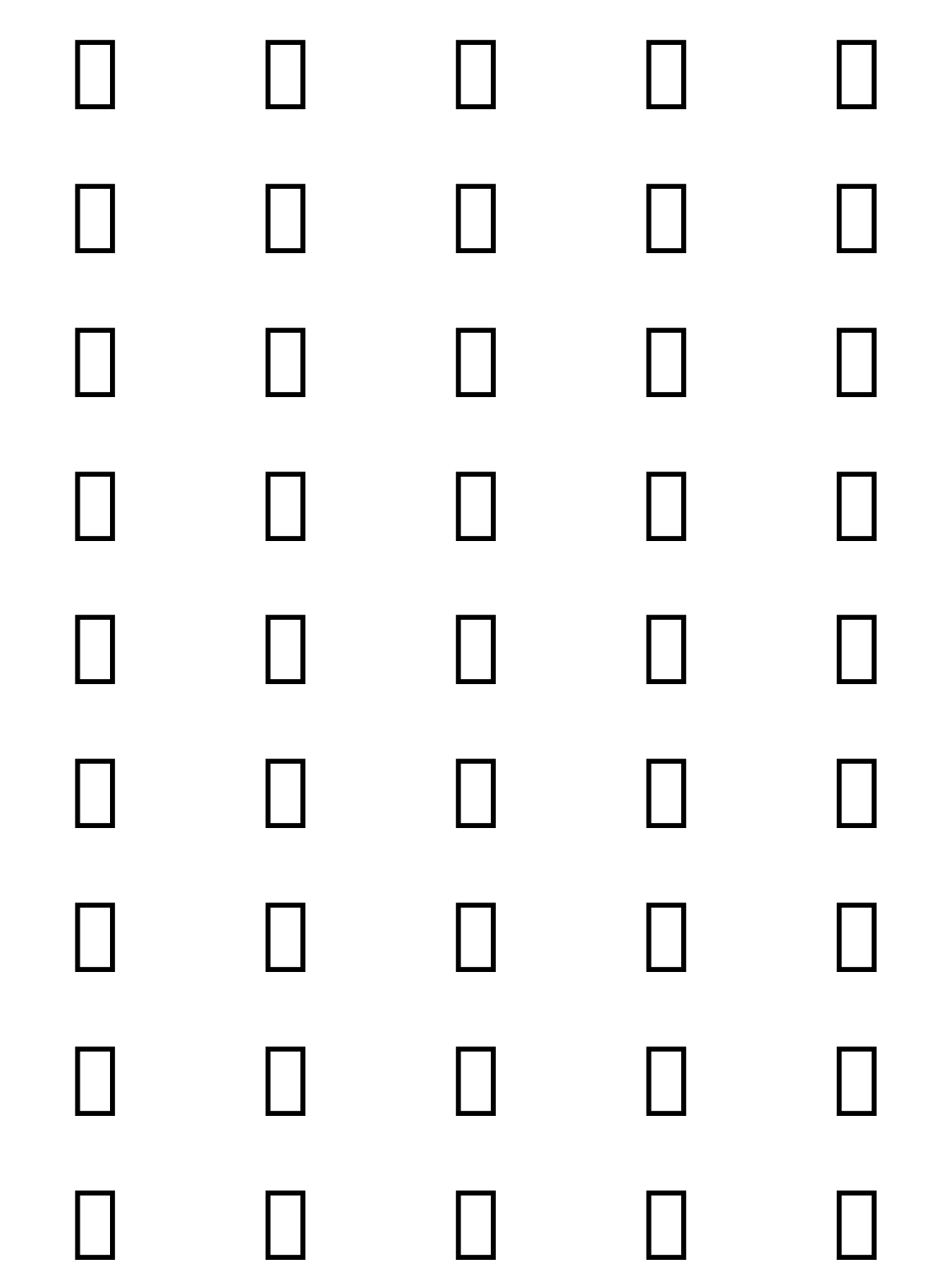

| え | か | の | て | み |
| --- | --- | --- | --- | --- |
| た | お | ん | け | ね |
| ら | る | こ | よ | に |
| も | め | つ | り | ひ |
| ろ | ふ | は | む | く |
| そ | あ | ほ | ま | ち |
| き | む | を | い | や |
| さ | ゆ | す | わ | と |
| れ | へ | せ | な | し |

## Slide 54
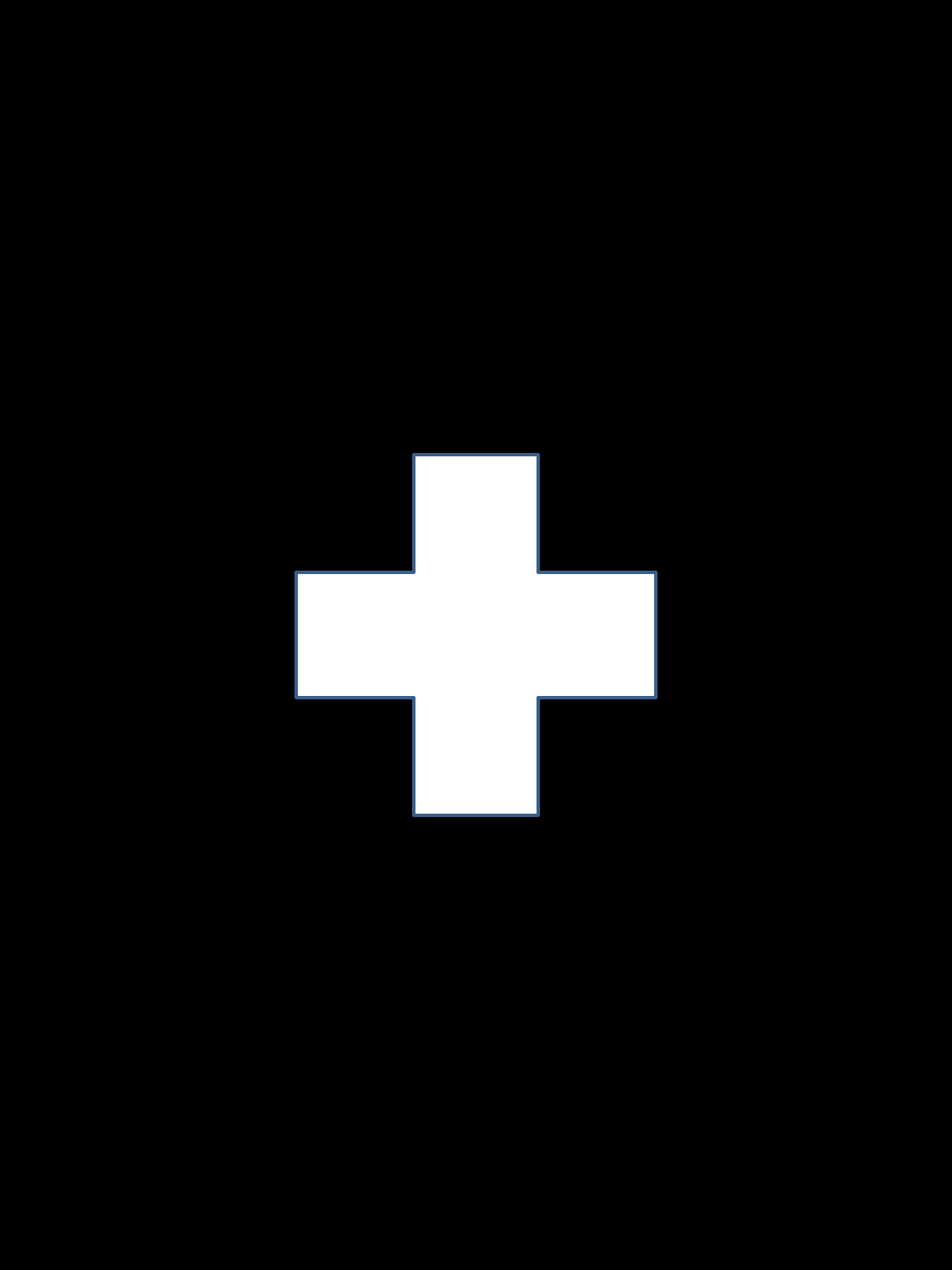

## Slide 55
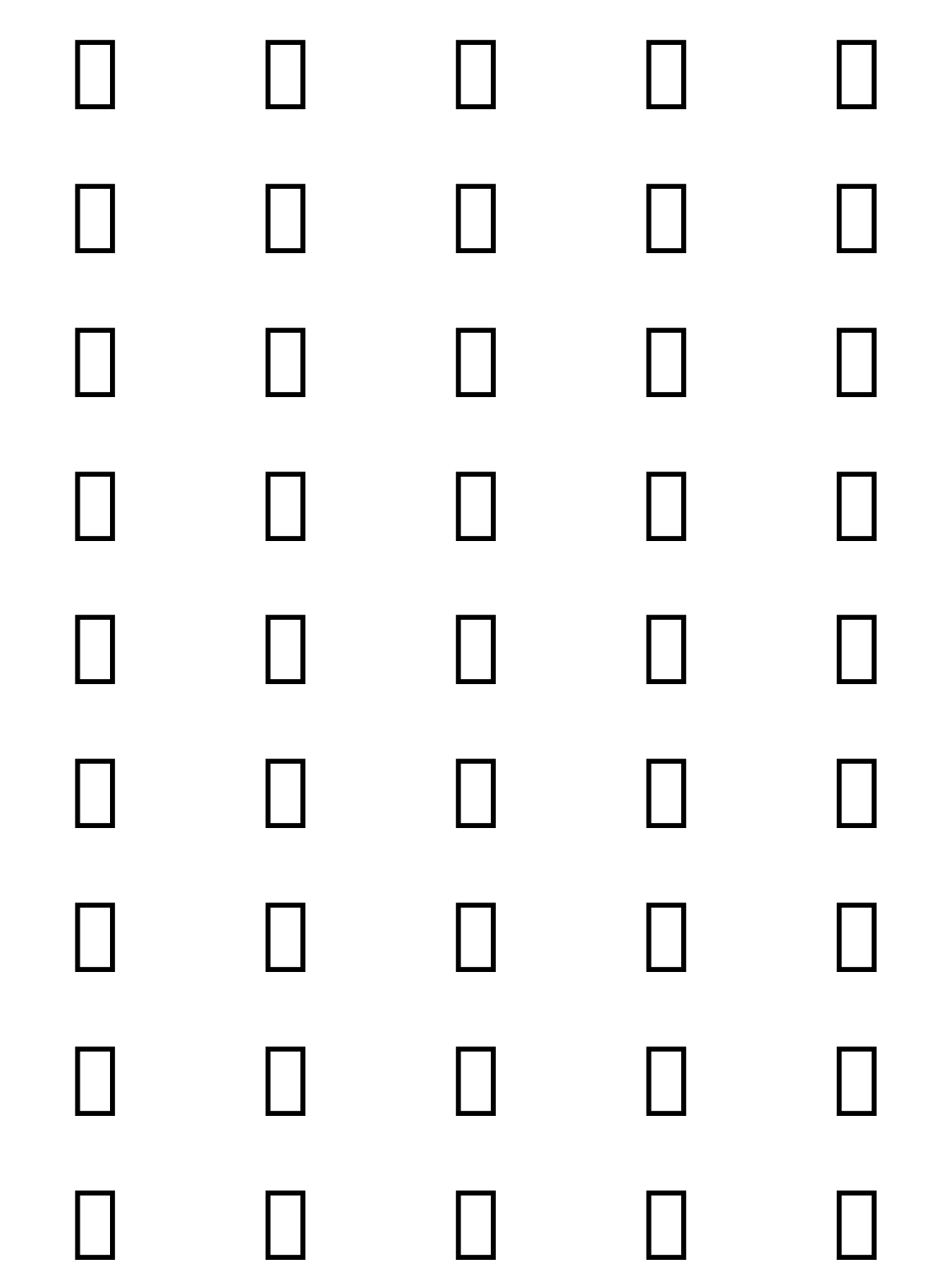

| し | な | せ | へ | れ |
| --- | --- | --- | --- | --- |
| と | わ | す | ゆ | さ |
| う | い | を | ぬ | き |
| ち | ま | ほ | あ | そ |
| く | む | は | ふ | ろ |
| ひ | り | つ | め | も |
| に | よ | こ | る | ら |
| ね | け | ん | お | た |
| み | て | の | か | え |

## Slide 56
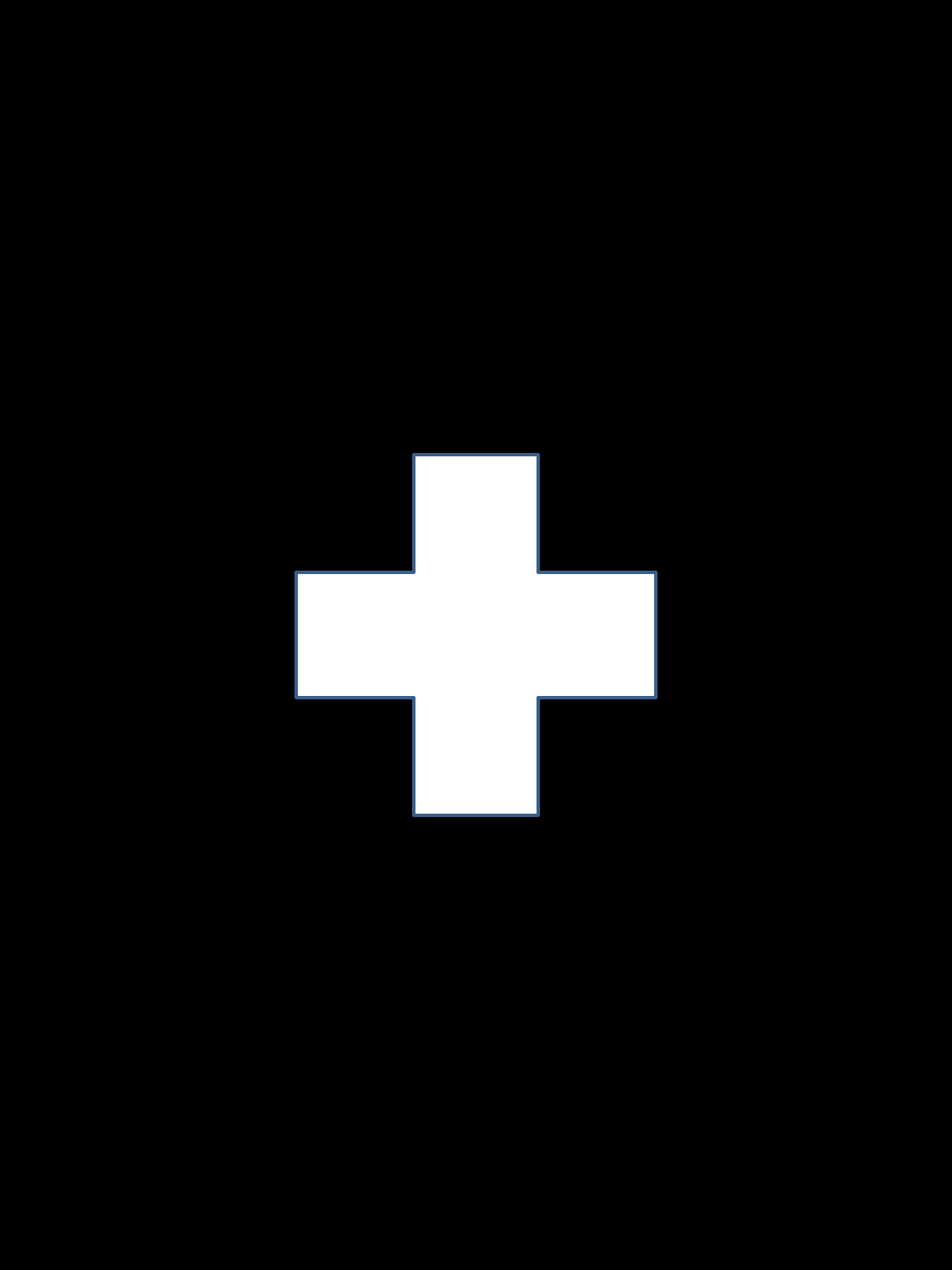

## Slide 57
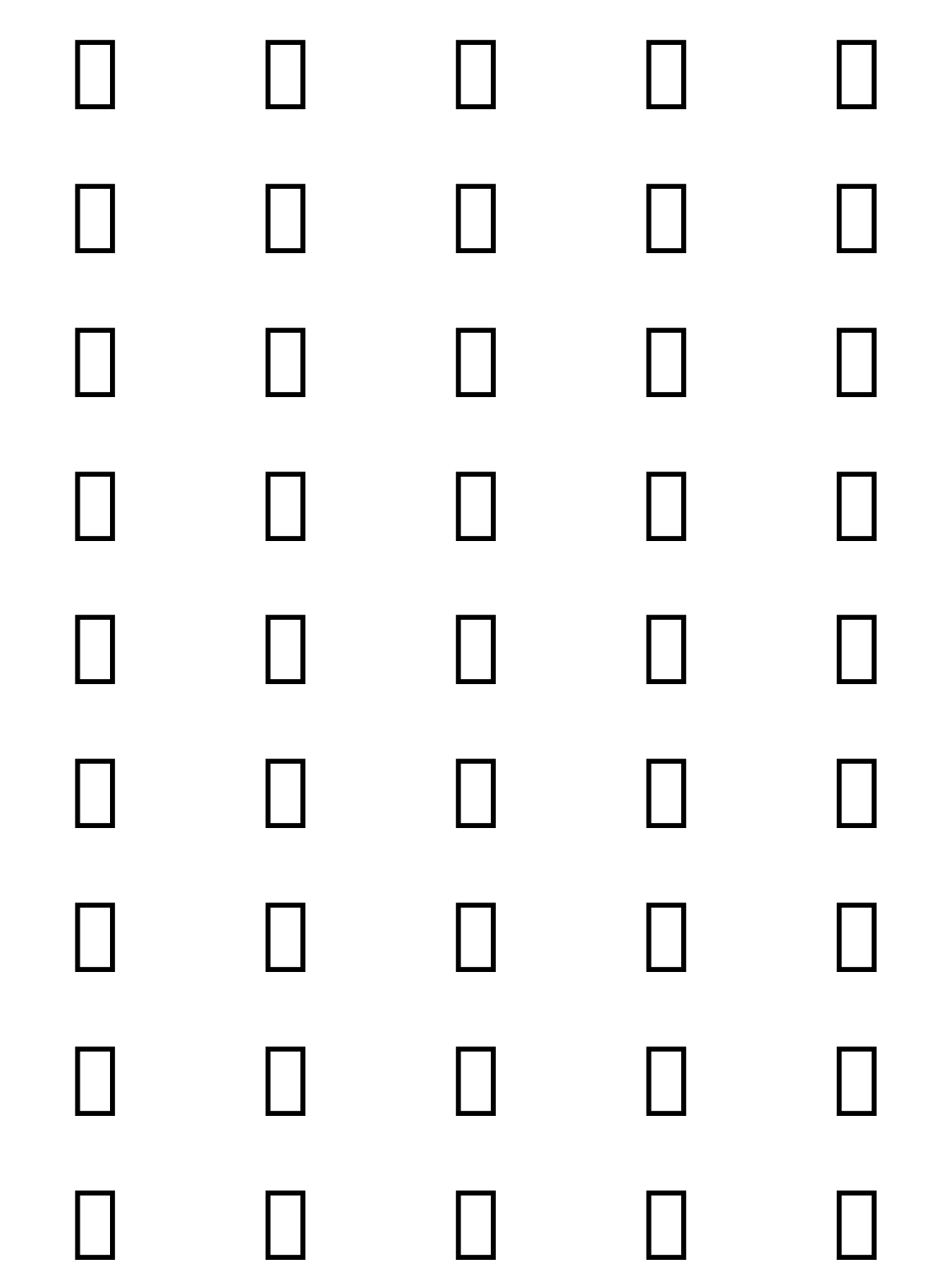

| う | な | す | せ | へ |
| --- | --- | --- | --- | --- |
| ち | や | し | そ | た |
| を | り | く | と | い |
| ひ | む | ゆ | ほ | ま |
| に | ね | よ | ぬ | ふ |
| み | て | け | ん | か |
| え | つ | ら | る | の |
| お | は | き | あ | れ |
| こ | ろ | わ | め | さ |

## Slide 58
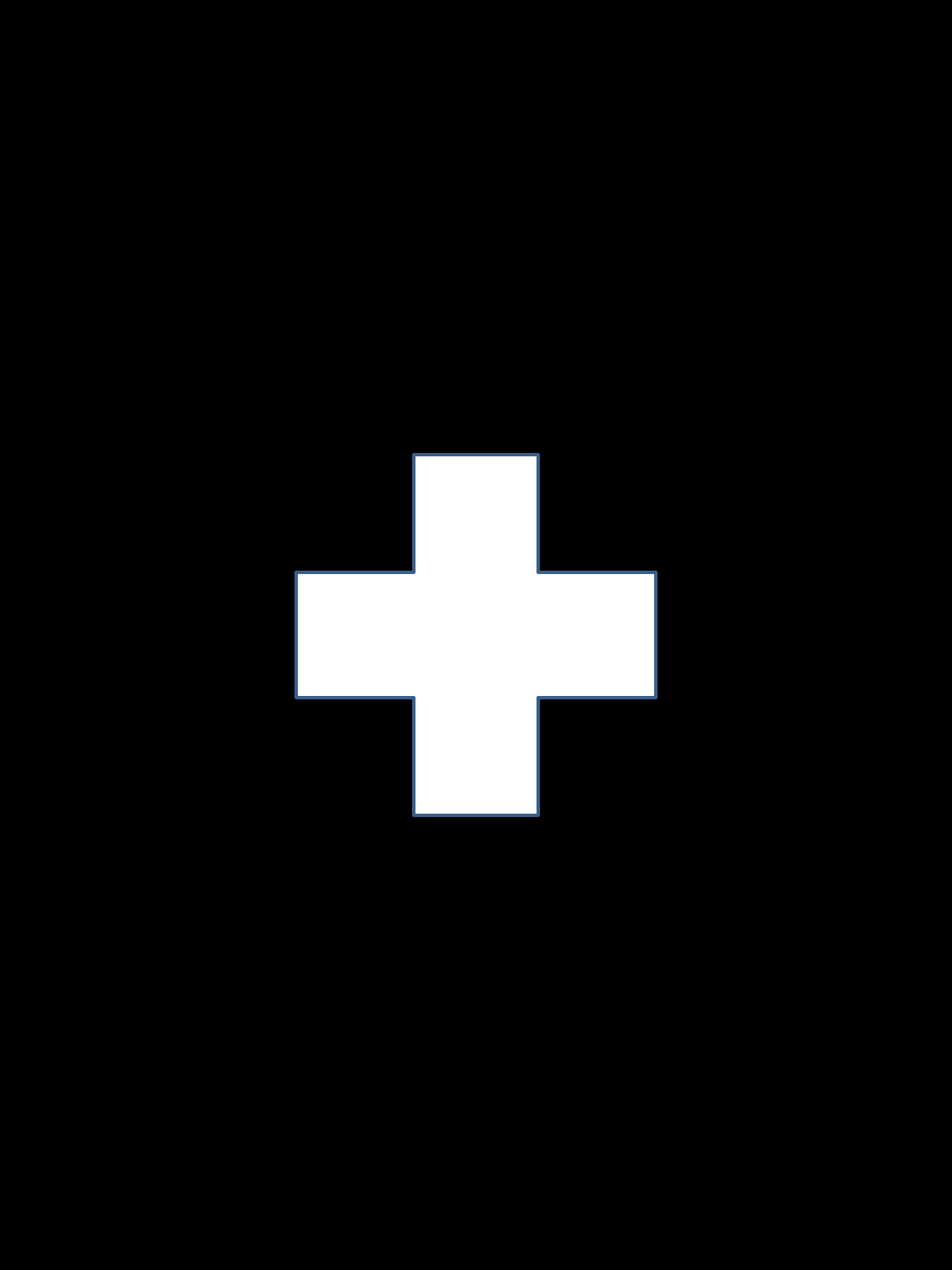

## Slide 59
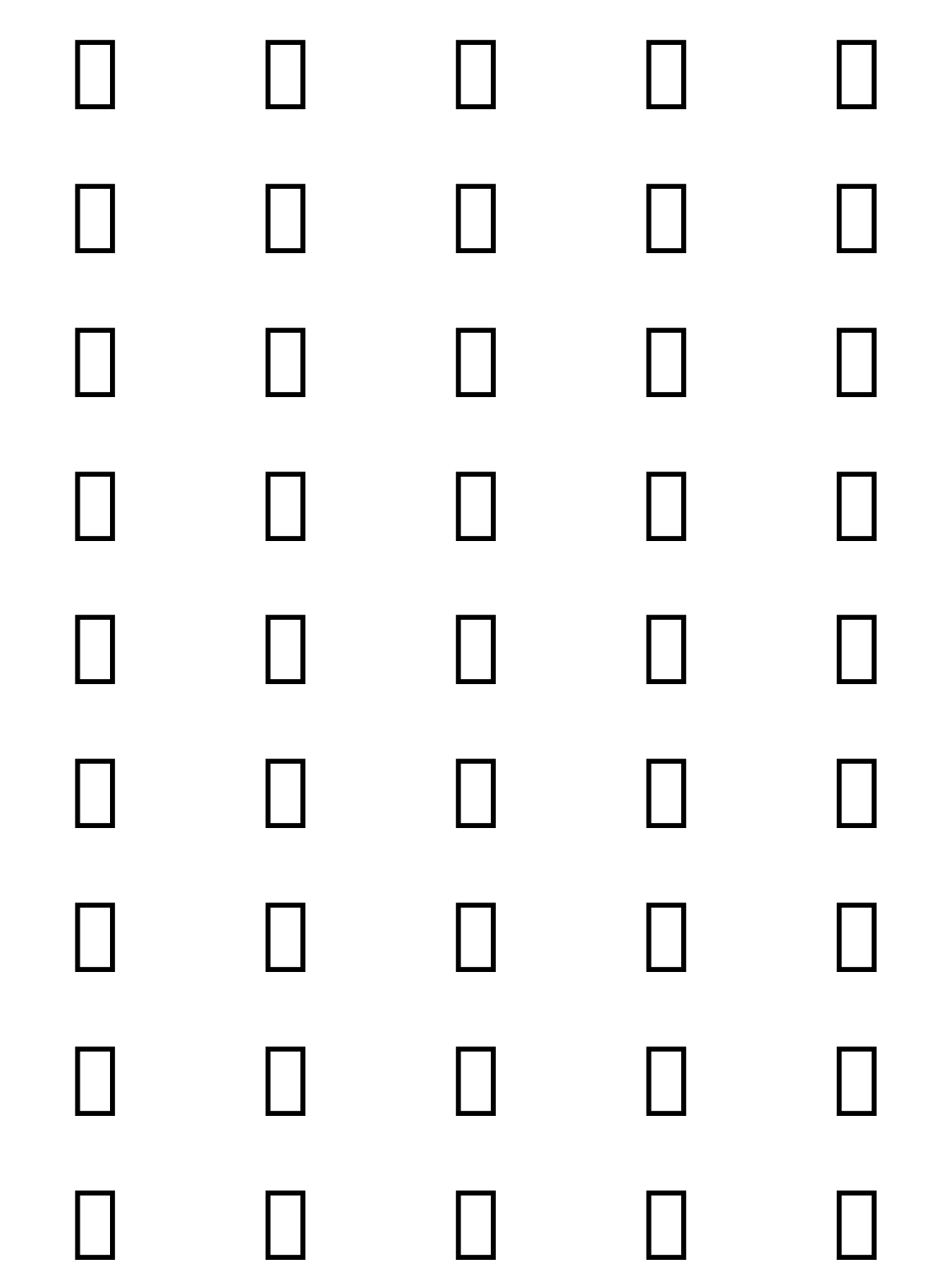

| へ | せ | す | な | も |
| --- | --- | --- | --- | --- |
| た | そ | し | や | ち |
| い | と | く | り | を |
| ま | ほ | ゆ | む | ひ |
| ふ | ぬ | よ | ね | に |
| か | ん | け | て | み |
| え | つ | ら | る | の |
| れ | あ | き | は | お |
| さ | あ | わ | う | こ |

## Slide 60
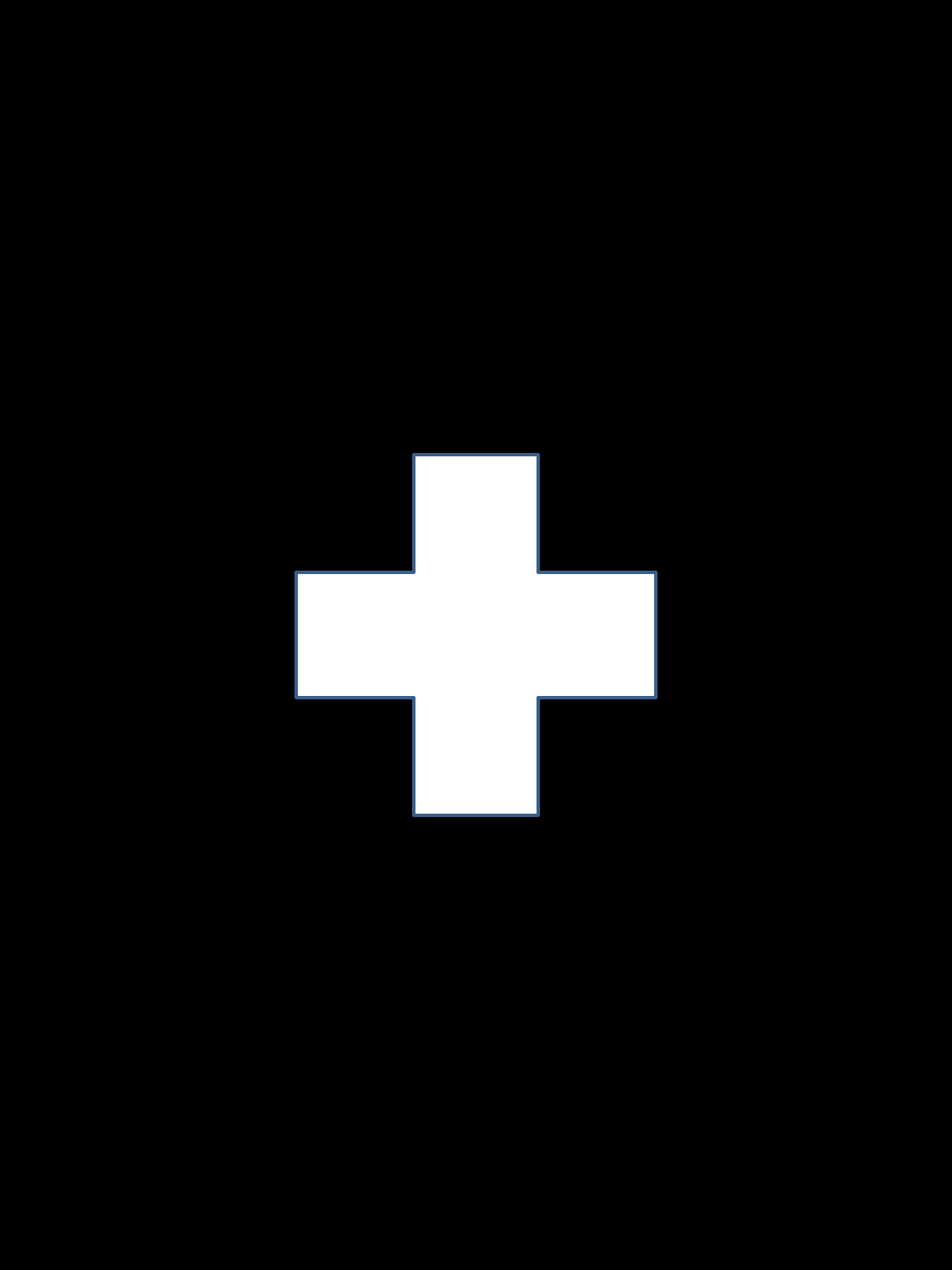

## Slide 61
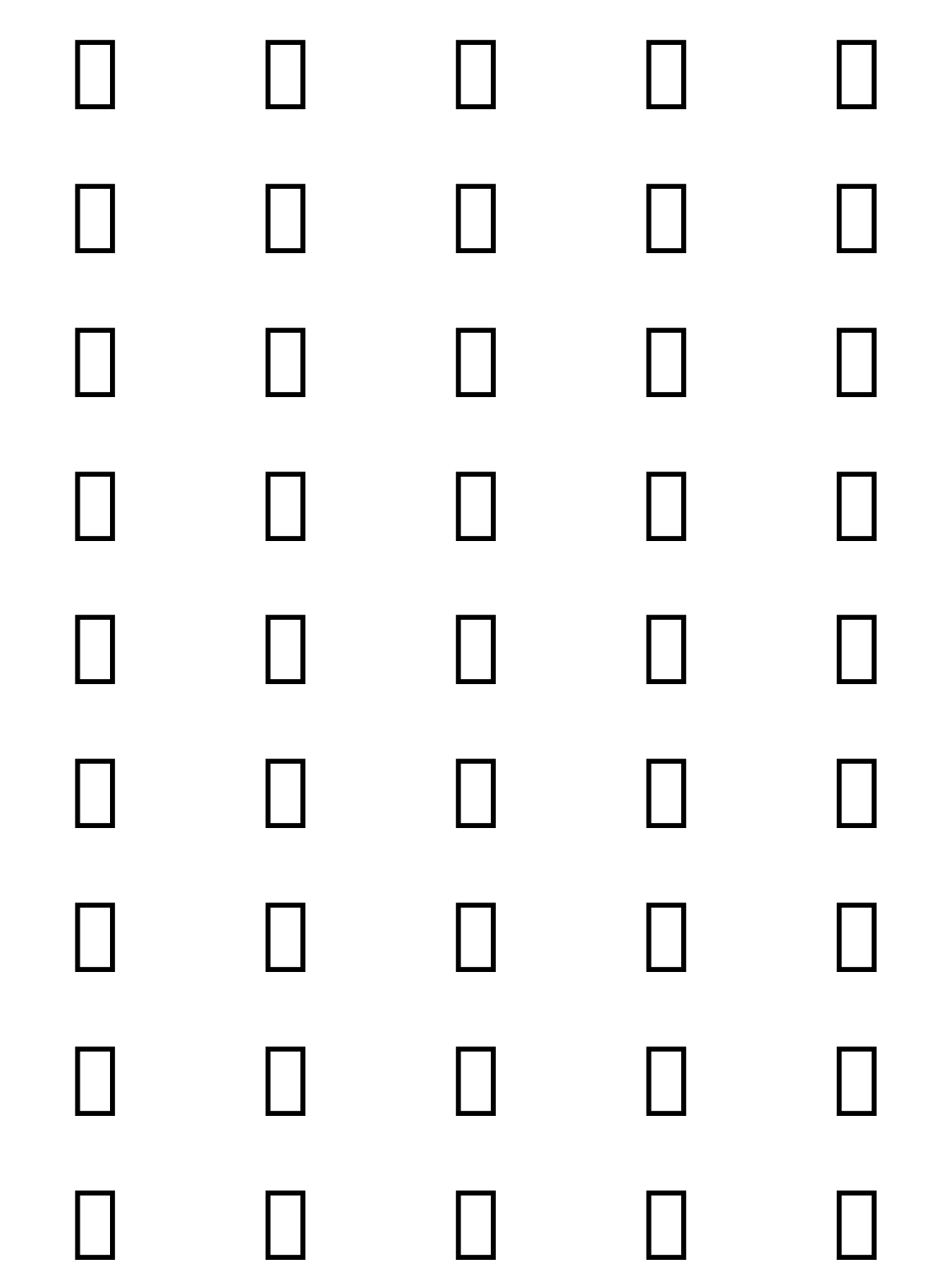

| よ | ろ | ち | む | や |
| --- | --- | --- | --- | --- |
| く | り | れ | る | て |
| は | す | お | め | ん |
| も | へ | あ | う | え |
| ゆ | ね | ほ | ひ | さ |
| き | に | け | せ | の |
| こ | ら | ま | た | い |
| な | ろ | そ | わ | つ |
| し | み | を | ふ | か |

## Slide 62
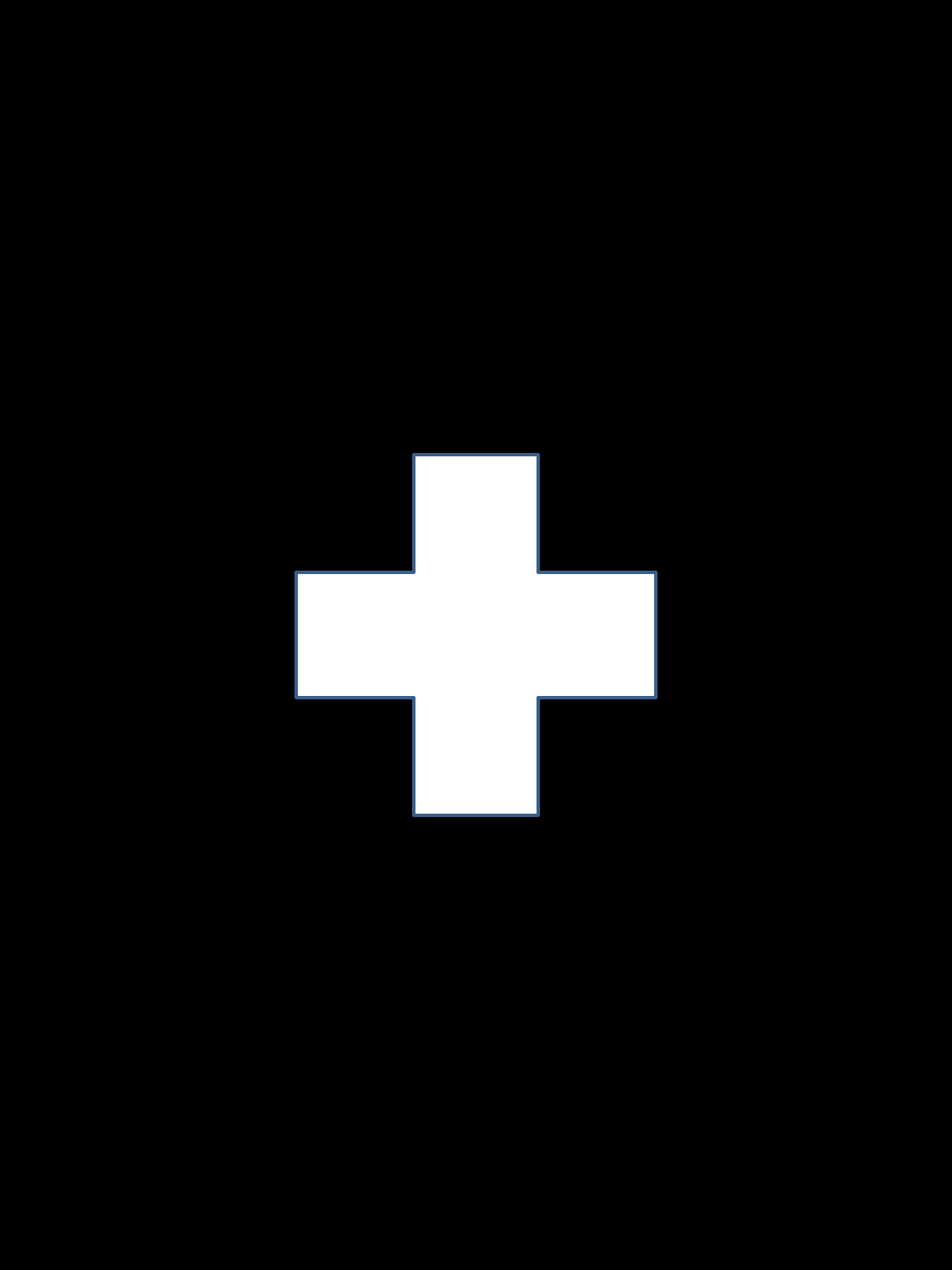

## Slide 63
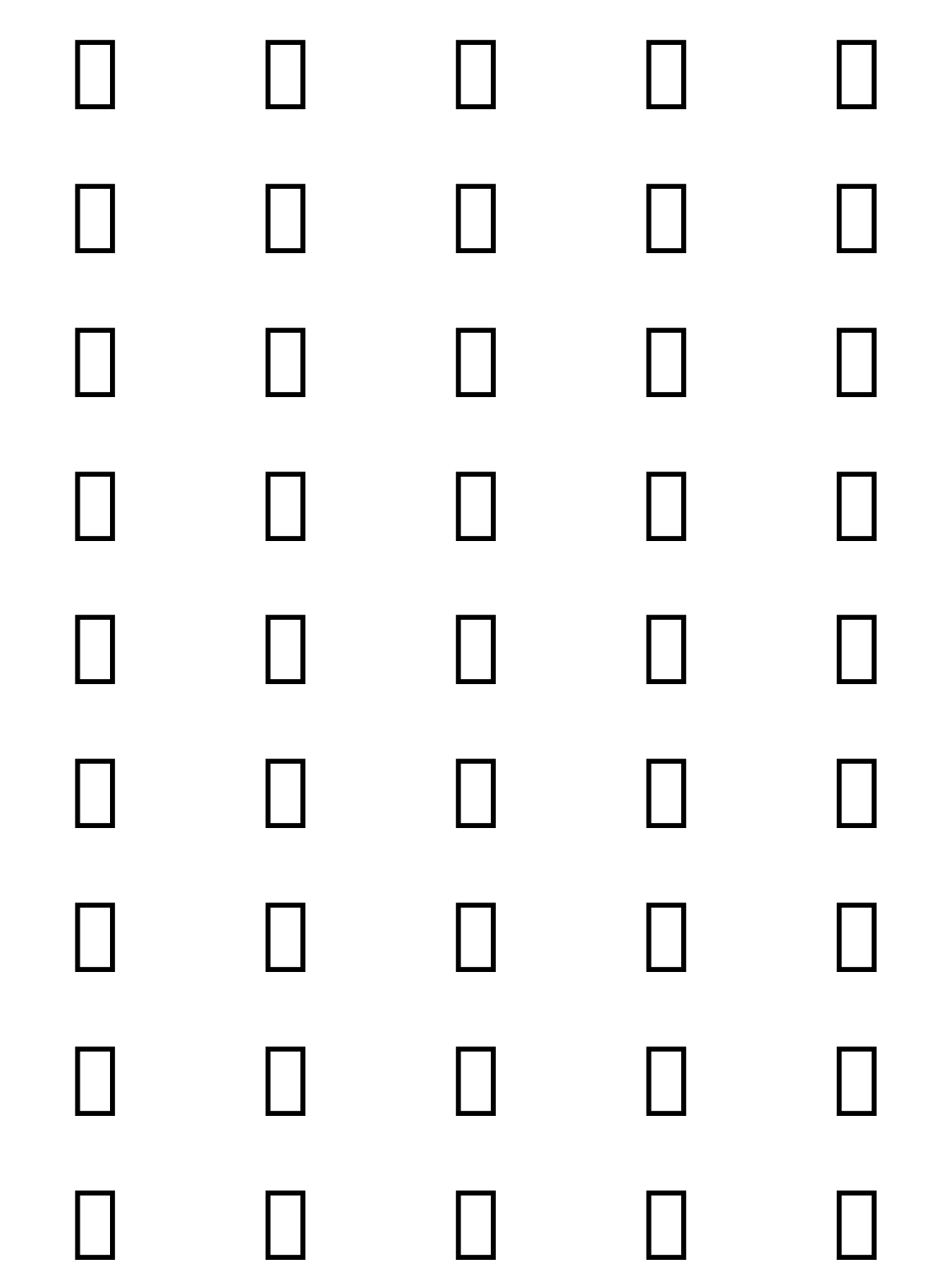

| う | い | や | く | な |
| --- | --- | --- | --- | --- |
| み | へ | は | を | ね |
| し | む | せ | ふ | ら |
| ぬ | こ | え | て | と |
| あ | わ | り | つ | た |
| す | よ | ほ | ひ | ち |
| け | わ | め | ま | ほ |
| お | に | か | き | も |
| ろ | さ | ん | る | の |
